# A Lewis acid-promoted Pinner reaction

**DOI:** 10.3762/bjoc.9.179

**Published:** 2013-08-02

**Authors:** Dominik Pfaff, Gregor Nemecek, Joachim Podlech

**Affiliations:** 1 Institut für Organische Chemie, Karlsruher Institut für Technologie (KIT), Fritz-Haber-Weg 6, 76131 Karlsruhe, Germany

**Keywords:** carbonitriles, carboxylic esters, Lewis acids, Pinner reaction, Ritter reaction

## Abstract

Carbonitriles and alcohols react in a Lewis acid-promoted Pinner reaction to carboxylic esters. Best results are obtained with two equivalents of trimethylsilyl triflate as Lewis acid. Good yields are achieved with primary alcohols and aliphatic or benzylic carbonitriles, but the straightforward synthesis of acrylates and benzoates starting with acrylonitrile and benzonitrile, respectively, is similarly possible. Phenols are not acylated under these reaction conditions. The method has been used for the first total synthesis of the natural product monaspilosin. In the reaction of benzyl alcohols variable amounts of amides are formed in a Ritter-type side reaction.

## Introduction

**Scheme 1:**
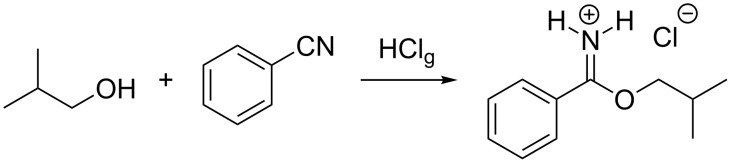
Imidate hydrochloride synthesis discovered by Pinner and Klein [1–2].

In 1877 Pinner and Klein discovered the proton-induced imidate syntheses [1–2]. They passed anhydrous gaseous hydrogen chloride through a mixture of isobutyl alcohol and benzonitrile. A crystalline product precipitated, which they identified as an imidate hydrochloride (Scheme 1). Best results in the Pinner reaction are obtained with primary or secondary alcohols and aliphatic or aromatic nitriles.

**Scheme 2:**
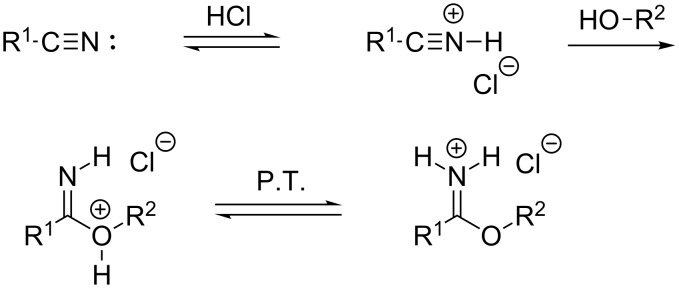
Mechanism of the Pinner reaction.

A plausible mechanism (Scheme 2) starts with a protonation of the nitrile by the strong acid hydrogen chloride leading to a highly activated nitrilium cation, which can be attacked by the alcohol component. Proton transfer (P.T.) yields the imidate hydrochloride [3].

**Scheme 3:**
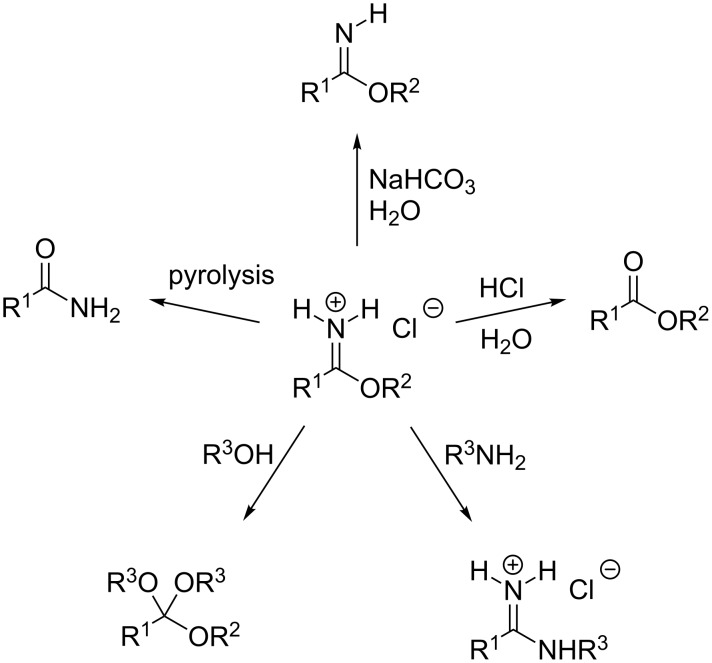
Transformations of imidate hydrochlorides.

Various transformations are possible with the imidate hydrochlorides: Hydrolysis at low pH leads to carboxylic esters, where basic hydrolysis yields imidates. Reaction with amines furnishes amidinium compounds and the reaction with alcohols gives rise to ortho esters. A less frequently used pyrolysis leads to carboxamides (Scheme 3) [3–5].

The harsh reaction conditions preclude a broad application of the Pinner reaction. The high toxicity and the laborious handling of gaseous hydrogen chloride are further drawbacks of this reaction. Nevertheless, milder protocols have developed over the decades: Luo and Jeevanandam used trimethylsilyl chloride (TMSCl) and ethanol for an in situ generation of hydrogen chloride [6]. Watanabe et al. reported on a Pinner reaction with a 4 N hydrogen chloride solution in cyclopentyl methyl ether (CPME) [7]. An ionic liquid based on a sulfonic acid was used by Jiang et al. [8], where this method has only been applied to aliphatic nitriles. A transition metal-catalyzed Pinner reaction using dihydridotetrakis(triphenylphosphano)ruthenium ([RuH_2_(PPh_3_)_4_]) as catalyst has been applied to aliphatic nitriles and alcohols and was similarly used for intramolecular reactions [9]. Schaefer et al. reported a base-catalyzed Pinner reaction, which gave only poor yields because of the setting of an equilibrium [10].

While developing a total synthesis of altenuic acid II [11], we observed the reaction of an aliphatic hydroxy group with acetonitrile in the presence of two equivalents of hafnium triflate [Hf(OTf)_4_] yielding the respective acetate. A detailed investigation on this reaction is reported in this article [12].

## Results and Discussion

**Scheme 4:**
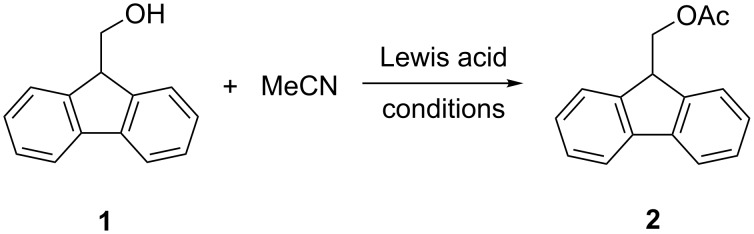
Reaction used for optimizations.

The Lewis acid-mediated Pinner reaction of aliphatic alcohols with nitriles was first observed by us, when hafnium triflate was present in the reaction mixture. We first aimed to replace this rather expensive Lewis acid with a more favorable substitute. As a test reaction for optimization we used the acylation of 9*H*-fluoren-9-ylmethanol (**1**) with acetonitrile as the nitrile component and solvent (Scheme 4). This substrate and the respective ester **2** are simply detected by thin-layer chromatography (TLC) and their molecular weights prevent losses during evaporation procedures.

A 72% yield was achieved, when two equivalents of hafnium(IV) triflate were used and when the nitrile was used as the solvent (Table 1, entry 1). Catalytic amounts of this Lewis acid led to unsatisfactory yields, when the reaction was performed in acetonitrile or in mixtures of acetonitrile with water (Table 1, entries 2 and 3). The 3% yield in acetonitrile/water (10:1) suggests that water is detrimental in the Pinner reaction. Among various tested Lewis acids, the best results were obtained with aluminium tribromide at 50 °C (65%, Table 1, entry 5) and with trimethylsilyl triflate at room temperature (83%, Table 1, entry 7). The less expensive trimethylsilyl chloride turned out to be an unsuitable alternative (Table 1, entry 8). The yield could not be improved, when two equivalents of aluminium bromide were used together with catalytic amounts of hafnium triflate (Table 1, entry 6).

**Table 1:** Selection of optimization experiments.

#	Lewis acid (equiv)	Conditions	Yield (s. m.)^a^ [%]

1	Hf(OTf)_4_ (2.0)	MeCN, rt, 48 h	72 (15)
2	Hf(OTf)_4_ (0.2)	MeCN, rt, 65 h	25 (69)
3	Hf(OTf)_4_ (0.2)	MeCN/H_2_O 10:1, rt, 65 h	3 (81)
4	AlBr_3_ (2.0)	MeCN, rt, 65 h	50 (39)
5	AlBr_3_ (2.0)	MeCN, 50 °C, 90 h	65 (20)
6	AlBr_3_ (2.0), Hf(OTf)_4_ (0.1)	MeCN, rt, 65 h	64 (24)
**7**	**TMSOTf (2.0)**	**MeCN, rt, 65 h**	**83 (9)**
8	TMSCl (2.0)	MeCN, rt, 65 h	33 (59)
9	TMSOTf (2.0)	MeCN, Et_3_N^b^, rt, 65 h	80 (10)

^a^Yields of recovered starting material (s. m.) given in parentheses. ^b^Et_3_N (1 equiv) was added.

**Scheme 5:**
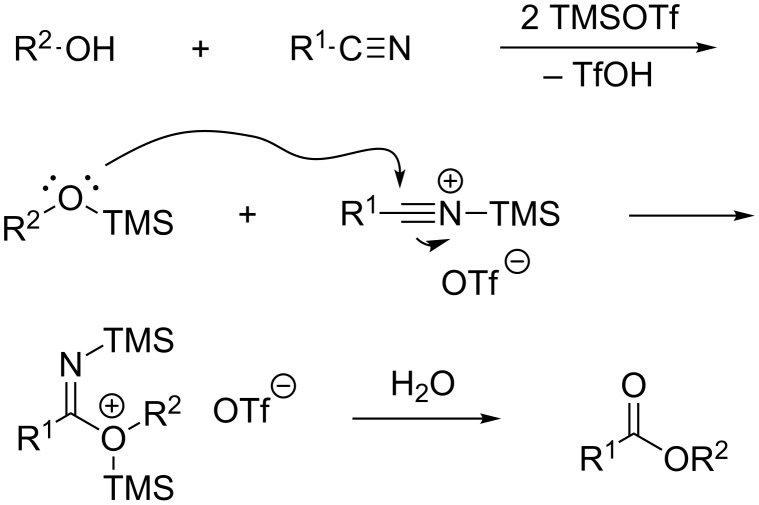
Plausible mechanism of the Lewis acid-promoted Pinner reaction.

Since two equivalents of a Lewis acid are necessary for optimum results, we presume activation of both the alcohol and the nitrile. A plausible mechanism includes formation of a silyl ether and an *N*-nitrilium cation. The former should be more nucleophilic than an alcohol and the latter should be an efficient electrophile (Scheme 5). Reaction of silyl ether and nitrilium cation leads to a cationic *N*,*O*-bis(trimethylsilyl)imino ester, which is hydrolyzed to a carboxylic ester. Formation of the Brønsted acid trifluoromethanesulfonic acid is to be expected under these reaction conditions, but seems to have no influence on the reaction outcome. A similar reaction with the addition of one equivalent of triethylamine led to a virtually identical yield (Table 1, entry 9).

With the optimized reaction conditions we tested a selection of nitriles and alcohols. All reactions required the application of the nitrile as solvent. Low yields were observed, when equimolar amounts of the nitrile and the alcohol were used in methylene chloride as inert solvent. The tested nitriles are acetonitrile, benzyl cyanide, benzonitrile, and acrylonitrile (Table 2).

**Table 2:** Variation of nitriles and alcohols.^a^

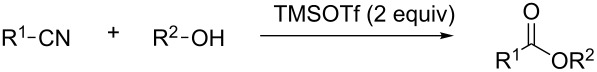					

#		R^1^ = Me	R^1^ = Bn	R^1^ = Ph	R^1^ = Vinyl
		
	R^2^−OH	Product, Yield [%]			

1	Fluorenylmethanol (**1**)	**2**, 83	**3**, 86	**4**, 44	**5**, 52 (67^b^)
2	Me(CH_2_)_9_OH (**6**)	**7**, 80	**8**, 85	**9**, 23	**10**, 29 (40^b^)
3	Cl(CH_2_)_6_OH (**11**)	**12**, 84	**13**, 90	**14**, 27	**15**, 38 (16^b^)
4	Et[O(CH_2_)_2_]_2_OH (**16**)	**17**, 75	**18**, 85	**19**, 26	**20**, 23 (19^b^)
5	*p*-NO_2_C_6_H_4_CH_2_OH (**21**)	**22**, 90	**23**, 78	**24**, 39	**25**, 85
6	*p*-HO_2_CC_6_H_4_CH_2_OH (**26**)	**27**, 88	**28**, 87	**29**, 31	**30**, 64
7	HO(CH_2_)_6_OH (**31**)	**32**, 32 (**33**, 19^c^)	**34**, 46 (**35**, 37^c^)	—	—
8	EtO_2_C(CH_2_)_5_OH (**36**)	**37**, 21	**38**, 16	**39**, 10	**40**, 7
9	*Z*-NH(CH_2_)_4_OH (**41**)	**42**, 13	**43**, 13	0	**44**, 14
10	cyHexOH (**45**)	**46**, 15	**47**, 25	—	—
11	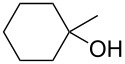 (**48**)	0	0	—	—
12	PhOH (**49**)	0	—	—	—
13	*p*-NO_2_C_6_H_4_OH (**50**)	0	—	—	—
14	3,4,5-Trimethoxyphenol (**51**)	0	—	—	—
15	*p*-MeOC_6_H_4_CH_2_OH (**52**)	0	0	0	0

^a^Alcohol (1 equiv), TMSOTf (2 equiv) dissolved in the nitrile (4 mL/mmol alcohol), rt, 65 h. ^b^TMSOTf (2 equiv) and nitrobenzene (1 equiv) were added. ^c^TMSOTf (4 equiv) was added. Yield of the monoacylated by-products **33** and **35**, respectively, in parentheses.

Best yields were obtained in the reactions of primary alcohols with acetonitrile and benzyl cyanide (Table 2, entries 1–4). Somewhat lower yields were observed with acrylonitrile; nevertheless, reported methods for the preparation of acrylate **5** lead to similar, and in some cases even lower yields [13–14]. A standard protocol for the synthesis of 9*H*-fluoren-9-ylmethyl acrylate starting with the moisture-sensitive acryloyl chloride under an inert atmosphere was reported to yield only 33% [15]. The herein reported Lewis acid promoted Pinner reaction furnished 52% of this substrate, where a less-sensitive substrate could be applied without the necessity of an inert atmosphere. The comparatively low yields in the reactions with benzonitrile are most probably caused by the lower electrophilicity of the benzonitrilium ion. The positive charge is significantly stabilized by the +M effect of the π system.

The high yields observed in the reactions of *para*-nitrobenzyl alcohol (**21**) and especially in its reaction with acrylonitrile brought us to the speculation that the nitro group has a special beneficial effect on this reaction, possibly as radical scavenger. It turned out that the addition of nitrobenzene [16] in the reactions with acrylonitrile led to increased yields in some cases. Other radical scavengers like hydroquinone had a similar effect.

Reaction of hexane-1,6-diol (**31**) gives moderate yields of the diacylated products, where significant amounts of the monoesters were isolated (Table 2, entry 7). Alcohols with further functional groups were similarly tested, but poor yields were observed, when ester or carbamate groups were present in the substrates (Table 2, entries 8 and 9). The Lewis acid trimethylsilyl triflate possibly cleaves the benzyloxycarbonyl (Z) group, since the similar trimethylsilyl iodide (TMSI) is known to cleave Z protecting groups [17]. Poor yields are obtained in the Lewis acid mediated reaction of nitriles with secondary alcohols such as cyclohexanol (Table 2, entry 10). No product at all was obtained, when the tertiary alcohol 1-methylcyclohexanol (**48**) was exposed to these conditions (Table 2, entry 11).

**Scheme 6:**
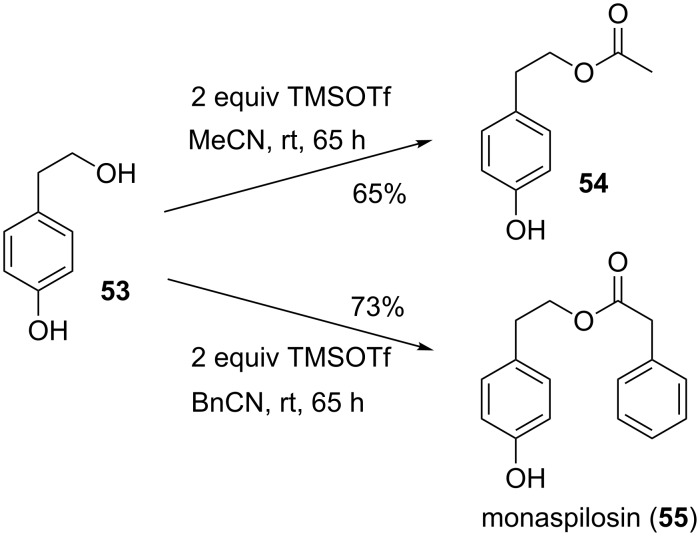
Synthesis of monaspilosin.

The Lewis acid promoted Pinner reaction is highly chemoselective; phenols were not acylated by these conditions and were re-isolated with high yields (Table 2, entries 12–14). In this context we tested 4-(2-hydroxyethyl)phenol (**53**) containing an aliphatic and a phenolic hydroxy function in the reaction with acetonitrile and benzyl cyanide, respectively (Scheme 6). The respective esters **54** and **55** were obtained with good yields: no esterification of the phenolic hydroxy group was observed. The reaction of 4-(2-hydroxyethyl)phenol (**53**) with benzyl cyanide yielded monaspilosin (**55**), an aromatic ester isolated from the mould fungus Monascus pilosus by Cheng et al. [18]. This compound was reported to have radical scavenger properties. The first total synthesis of this natural product was herewith achieved with 73% yield in only one step.

**Scheme 7:**
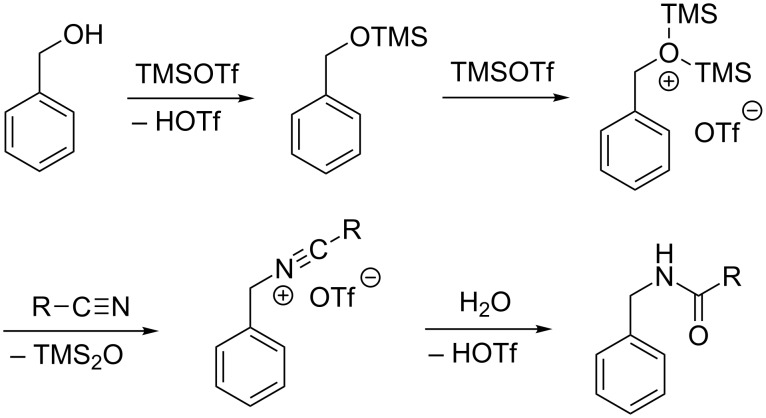
Proposed mechanism of the trimethylsilyl triflate-promoted Ritter reaction.

Good yields were observed, when benzyl alcohols with electron withdrawing (−M) substituents such as 4-nitrobenzyl alcohol (**21**, Table 2, entry 5) and 4-hydroxymethylbenzoic acid (**26**, Table 2, entry 6) were reacted with benzyl cyanide or acetonitrile and even with acrylonitrile. A protection of the carboxylic acid turned out to be not necessary. In contrast, a 4-methoxy-substituted benzyl alcohol **52**, i.e., an electron-rich benzyl alcohol, furnished no carboxylic ester at all (Table 2, entry 15). Rather poor yields of the respective carboxylic esters were achieved, when unsubstituted benzyl alcohol (**56**) or 4-fluorobenzyl alcohol (**64**) were reacted with one of the carbonitriles (Table 3). Instead we isolated significant amounts of carboxamides. These amides result from a Ritter-type reaction [19–21], where a carbenium ion (or a substrate with significant positive partial charge) reacts at the nitrogen atom of a nitrile. This transformation is a competition to the Pinner reaction, when benzyl alcohols are used. A possible mechanism of this reaction is given in Scheme 7. Double silylation leads to the formation of a good leaving group and the highly electrophilic benzylic carbon is attacked by the nitrile yielding a nitrilium cation. The reaction is finalized by hydrolysis furnishing the carboxamide. Apparently the double silylation is not possible with electron-deficient substrates.

**Table 3:** Carboxamide formation in a Pinner-type reaction.^a^

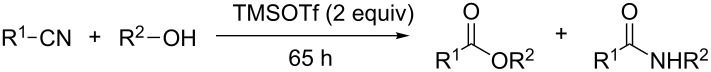				

#	R^1^	R^2^	Yield [%] (Product)	
	
			Ester	Amide

1	Me	Bn (**56**)	41 (**57**)	18 (**58**)
2	Bn	Bn (**56**)	18 (**59**)	59 (**60**)
3	Ph	Bn (**56**)	0	66 (**61**)
4	Vinyl	Bn (**56**)	4 (**62**)	90 (**63**)
5	Me	*p*-FC_6_H_4_CH_2_ (**64**)	29 (**65**)	63 (**66**)
6	Bn	*p*-FC_6_H_4_CH_2_ (**64**)	0	79 (**67**)
7	Ph	*p*-FC_6_H_4_CH_2_ (**64**)	0	70 (**68**)
8	Vinyl	*p*-FC_6_H_4_CH_2_ (**64**)	0	89 (**69**)

^a^Alcohol (1 equiv), TMSOTf (2 equiv) dissolved in the nitrile (4 mL/mmol alcohol), rt, 65 h.

## Conclusion

The Lewis acid-promoted Pinner reaction is a mild and chemoselective alternative for the synthesis of carboxylic esters starting with alcohols and nitriles. The esterification of primary aliphatic alcohols is possible even in the presence of unprotected carboxylic acids or phenolic hydroxy groups.

## Supporting Information

File 1 Experimental section and NMR spectra of all synthesized compounds.
