# Supplementary material for: A Lewis acid-promoted Pinner reaction
Source: Beilstein J Org Chem. 2013 Aug 2;9:1572–7. doi: 10.3762/bjoc.9.179 (PMC3740506; doi:10.3762/bjoc.9.179)

Supporting Information

for

A Lewis acid-promoted Pinner reaction

Dominik Pfaff, Gregor Nemecek and Joachim Podlech*

Address: Institut für Organische Chemie, Karlsruher Institut für Technologie (KIT), Fritz-Haber-Weg 6, 76131 Karlsruhe, Germany

Email: Joachim Podlech - joachim.podlech@kit.edu

* Corresponding author

Experimental section and NMR spectra of all synthesized compounds.

Content:

A. Experimental Section

B. ^{1}H and ^{13}C NMR spectra of:

1. (9*H*-Fluoren-9-yl)methyl acetate (**2**)
2. (9*H*-Fluoren-9-yl)methyl phenylacetate (**3**)
3. (9*H*-Fluoren-9-yl)methyl benzoate (**4**)
4. (9*H*-Fluoren-9-yl)methyl acrylate (**5**)
5. 1-Decyl acetate (**7**)
6. 1-Decyl phenylacetate (**8**)
7. 1-Decyl benzoate (**9**)
8. 1-Decyl acrylate (**10**)
9. 6-Chlorohexyl acetate (**12**)
10. 6-Chlorohexyl phenylacetate (**13**)
11. 6-Chlorohexyl benzoate (**14**)
12. 6-Chlorohexyl acrylate (**15**)
13. 2-(2-Ethoxyethoxy)ethyl acetate (**17**)
14. 2-(2-Ethoxyethoxy)ethyl phenylacetate (**18**)
15. 2-(2-Ethoxyethoxy)ethyl benzoate (**19**)
16. 2-(2-Ethoxyethoxy)ethyl acrylate (**20**)
17. 4-Nitrobenzyl acetate (**22**)
18. 4-Nitrobenzyl phenylacetate (**23**)
19. 4-Nitrobenzyl benzoate (**24**)
20. 4-Nitrobenzyl acrylate (**25**)
21. 4-(Acetoxymethyl)benzoic acid (**27**)
22. 4-[(Phenylacetoxy)methyl]benzoic acid (**28**)
23. 4-(Benzoyloxymethyl)benzoic acid (**29**)
24. 4-(Acryloyloxymethyl)benzoic acid (**30**)
25. Hexane-1,6-diyl diacetate (**32**)
26. 6-Hydroxyhexyl acetate (**33**)
27. Hexane-1,6-diyl bis(phenylacetate) (**34**)
28. 6-Hydroxyhexyl phenylacetate (**35**)
29. Ethyl 6-acetoxyhexanoate (**37**)
30. Ethyl 6-(phenylacetoxy)hexanoate (**38**)
31. 6-Ethoxy-6-oxohexyl benzoate (**39**)
32. Ethyl 6-(acryloyloxy)hexanoate (**40**)
33. 4-(Benzyloxycarbonylamino)butyl acetate (**42**)
34. 4-(Benzyloxycarbonylamino)butyl phenylacetate (**43**)
35. 4-(Benzyloxycarbonylamino)butyl acrylate (**44**)
36. Cyclohexyl acetate (**46**)
37. Cyclohexyl phenylacetate (**47**)
38. 2-(4-Hydroxyphenyl)ethyl acetate (**54**)
39. 2-(4-Hydroxyphenyl)ethyl phenylacetate, pmonaspilosin (**55**)
40. Benzyl acetate (**57**)
41. *N*-Benzylacetamide (58)
42. Benzyl phenylacetate (**59**)
43. *N*-Benzyl-phenylacetamide (**60**)
44. *N*-Benzylbenzamide (**61**)
45. Benzyl acrylate (**62**)

46 *N*-Benzylacrylamide (**63**)

47. 4-Fluorobenzyl acetate (**65**)

48 *N*-4-(Fluorobenzyl)acetamide (**66**)

49. *N*-(4-Fluorobenzyl)phenylacetamide (**67**)
50. *N*-(4-Fluorobenzyl)benzamide (**68**)
51. *N*-(4-Fluorobenzyl)acrylamide (**69**)

A. Experimental Section

General. Flash column chromatography was carried out using Merck SiO_2 60 (230–400 mesh),[1] and thin layer chromatography (TLC) was carried out using commercially available Merck F254 precoated sheets. ^{1}H and ^{13}C NMR spectra were recorded on a Bruker AVANCE 300 or on an AM-400 spectrometer. Chemical shifts are given in ppm and were referenced using residual signals of the solvent as internal standard (^{1}H: $CHCl_3$, 7.26, acetone, 2.05; ^{13}C: $CDCl_3$, 77.16). IR spectra were recorded on a Bruker IFS-88 spectrometer. Mass spectra were recorded on a Finnigan MAT-90 mass spectrometer.

General procedure (GP 1) for reactions with acetonitrile or acrylonitrile.[2] TMSOTf (336 mg, 1.51 mmol) is added to a solution of the alcohol (0.755 mmol) in the respective nitrile (3 mL) and the mixture is stirred at rt for 65 h. H_2O (25 mL) and brine (25 mL) are added, and the mixture is extracted with EtOAc (3 × 30 mL). The combined organic layers are dried (Na_2SO_4) and concentrated. The crude product is purified by flash column chromatography (silica gel).

General procedure (GP 2) for reactions with acrylonitrile in the presence of nitrobenzene. TMSOTf (336 mg, 1.51 mmol) is added to a solution of the alcohol (0.755 mmol) and nitrobenzene (93 mg, 0.755 mmol) in the respective nitrile (3 mL) and the mixture is stirred at rt for 65 h. H_2O (25 mL) and brine (25 mL) are added, and the mixture is extracted with EtOAc (3 × 30 mL). The combined organic layers are dried (Na_2SO_4) and concentrated. The crude product is purified by flash column chromatography (silica gel).

General procedure (GP 3) for reactions with benzyl cyanide or benzonitrile.[2] TMSOTf (336 mg, 1.51 mmol) is added to a solution of the alcohol (0.755 mmol) in the respective nitrile (3 mL) and the mixture is stirred at rt for 65 h. H_2O (25 mL) and brine (25 mL) are added, and the mixture is extracted with EtOAc (3 × 30 mL). The combined organic layers are dried (Na_2SO_4) and concentrated, and excess nitrile is distilled off at reduced pressure (90 °C, 0.1 mbar). The crude product is purified by flash column chromatography (silica gel).

(9*H*-Fluoren-9-yl)methyl acetate (2). 9*H*-Fluoren-9-ylmethanol (**1**) and MeCN were reacted according to GP 1. Chromatography (hexanes/EtOAc, 9:1) afforded **2** (152 mg, 83%) as a yellow solid. R_f = 0.53 (hexanes/EtOAc, 3:1). ^{1}H NMR (300 MHz, $CDCl_3$) δ 2.16 (s, 3H, Me), 4.22 (t, ^{3}J = 7.2 Hz, 1H, 9'-H), 4.37 (d, ^{3}J = 7.2 Hz, 2H, CH_2), 7.33 (t, ^{3}J = 7.4 Hz, 2H, 2'-H, 7'-H), 7.42 (t, ^{3}J = 7.4 Hz, 2H, 3'-H,

---
1 Still, W. C.; Kahn, M.; Mitra, A. *J. Org. Chem.*, **1978**, *43*, 2923–2925.
2 Pfaff, D.; Nemecek, G.; Podlech, J. *Helv. Chim. Acta*, **2012**, *95*, 1851−1856.

6’-H), 7.61 (d, ^3J = 7.4 Hz, 2H, 1’-H, 8’-H), 7.78 (d, ^3J = 7.4 Hz, 2H, 4’-H, 5’-H). The spectrum is in full agreement with published data.[3]

(9*H*-Fluoren-9-yl)methyl phenylacetate (3). 9*H*-Fluoren-9-ylmethanol (**1**) and BnCN were reacted according to GP 3. Chromatography (hexanes/EtOAc, 9:1) afforded **3** (207 mg, 86%) as a yellow oil. R_f = 0.59 (hexanes/EtOAc, 3:1). IR (KBr): $\tilde{\nu}$ (cm^{-1}) = 3452 (w), 3064 (m), 3032 (m), 2949 (m), 2891 (w), 1732 (s, C=O), 1451 (s), 1255 (s), 1148 (s), 1007 (m), 758 (s), 740 (s), 543 (m), 426 (m). ^{1}H NMR (400 MHz, $CDCl_3$) δ 3.71 (s, 2H, *CH_2*Ph), 4.18 (t, ^3J = 7.2 Hz, 1H, 9’-H), 4.39 (d, ^3J = 7.2 Hz, 2H, OCH_2), 7.22–7.42 (m, 11H, H_{ar}), 7.75 (d, ^3J = 7.5 Hz, 4’-H, 5’-H). ^{13}C NMR (100 MHz, $CDCl_3$) δ 41.8 (t), 46.8 (d), 66.9 (t), 120.1 (2 d), 125.2 (d), 127.2 (2 d), 127.3 (2 d), 127.9 (2 d), 128.8 (2 d), 129.5 (2 d), 134.0 (s), 141.4 (2 s), 143.8 (2 s), 171.6 (s). MS (EI, 90 °C): *m/z* (%) = 314 (10) [M^+], 179 (16), 178 (100), 165 (19), 91 (19). HRMS ($^{12}C_{22}{}^{1}H_{18}{}^{16}O_2$, EI): calcd. 314.1307 amu; found 314.1309 amu. Anal. calcd for $C_{22}H_{18}O_2$ (314.13): C 84.05, H 5.77; found C 83.64, H 5.64.

(9*H*-Fluoren-9-yl)methyl benzoate (4). 9*H*-Fluoren-9-ylmethanol (**1**) and benzonitrile were reacted according to GP 3. Chromatography (hexanes/EtOAc, 9:1) afforded **4** (100 mg, 44%) as a yellow solid. R_f = 0.59 (hexanes/EtOAc, 3:1). ^{1}H NMR (300 MHz, $CDCl_3$) δ 4.40 (t, ^3J = 7.2 Hz, 1H, 9’-H), 4.62 (d, ^3J = 7.2 Hz, 2H, CH_2), 7.33 (t, ^3J = 7.4 Hz, 2H, 2’-H, 7’-H), 7.43 (t, ^3J = 7.4 Hz, 2H, 3’-H, 6’-H), 7.50 (t, ^3J = 7.5 Hz, 2H, Ph), 7.61 (t, ^3J = 7.4 Hz, 1H, Ph), 7.67 (d, ^3J = 7.4 Hz, 2H, 1’-H, 8’-H), 7.80 (d, ^3J = 7.4 Hz, 2H, 4’-H, 5’-H), 8.11 (d, ^3J = 7.8 Hz, 2H, Ph). The spectrum is in full agreement with published data.[4]

(9*H*-Fluoren-9-yl)methyl acrylate (5). 9*H*-Fluoren-9-ylmethanol (**1**) and acrylonitrile were reacted according to GP 1. Chromatography (hexanes/EtOAc, 9:1) afforded **5** (100 mg, 52%) as a yellow oil. A similar reaction performed according to GP 2 yielded **5** (127 mg, 67%). R_f = 0.56 (hexanes/EtOAc, 3:1). ^{1}H NMR (300 MHz, $CDCl_3$) δ 4.28 (t, ^3J = 7.3 Hz, 1H, 9’-H), 4.46 (d, ^3J = 7.3 Hz, 2H, CH_2), 5.91 (d, ^3J = 10.3 Hz, 1H, 3-H_{trans}), 6.23 (dd, ^3J = 17.3 Hz, ^3J = 10.3 Hz, 1H, 2-H), 6.48 (d, ^3J = 17.3 Hz, 1H, 3-H_{cis}), 7.33 (t, ^3J = 7.4 Hz, 2H, 2’-H, 7’-H), 7.42 (t, ^3J = 7.4 Hz, 2H, 3’-H, 6’-H), 7.61 (d, ^3J = 7.4 Hz, 2H, 1’-H, 8’-H), 7.78 (d, ^3J = 7.4 Hz, 2H, 4’-H, 5’-H). The spectrum is in full agreement with published data.[5]

1-Decyl acetate (7). 1-Decanol (**6**) and MeCN were reacted according to GP 1. Chromatography (hexanes/EtOAc, 9:1) afforded **7** (121 mg, 80%) as a colourless oil. R_f = 0.63 (hexanes/EtOAc, 3:1).

---

3 Débieux, J.-L.; Cosandey, A.; Helgen, C.; Bochet, C. G. *Eur. J. Org. Chem.*, **2007**, 2073–2077.
4 More O'Ferrall, R. A.; Larkin, F.; Walsh, P. *J. Chem. Soc., Perkin Trans. 2*, **1982**, 1573–1579.
5 Schild, H. G.; Tirrell, D. A. *Macromolecules*, **1992**, *25*, 4553–4558.

^{1}H NMR (300 MHz, $CDCl_3$) δ 0.88 (t, ^3J = 6.6 Hz, 3H, 10'-H_3), 1.20–1.36 [m, 14H, Me(CH_2)$_7$], 1.61 (tt, ^3J = 6.8 Hz, ^3J = 6.4 Hz, 2H, 2'-H_2), 2.04 (s, 3H, OAc), 4.05 (t, ^3J = 6.8 Hz, 2H, 1'-H_2). The spectrum is in full agreement with published data.[6]

1-Decyl phenylacetate (8). 1-Decanol (**6**) and BnCN were reacted according to GP 3. Chromatography (hexanes/EtOAc, 9:1) afforded **8** (177 mg, 85%) as a yellowish oil. R_f = 0.69 (hexanes/EtOAc 3:1). ^{1}H NMR (300 MHz, $CDCl_3$) δ 0.89 (t, ^3J = 6.7 Hz, 3H, Me), 1.21–1.35 [m, 14H, Me(CH_2)$_7$], 1.55–1.65 (m, 2H, 2'-H_2), 3.61 (s, 2H, CH_2Ph), 4.08 (t, ^3J = 6.7 Hz, 2H, 1'-H_2), 7.22–7.38 (m, 5H, Ph). The spectrum is in full agreement with published data.[7]

1-Decyl benzoate (9). 1-Decanol (**6**) and PhCN were reacted according to GP 3. Chromatography (hexanes/EtOAc, 19:1) afforded **9** (45 mg, 23%) as a yellowish oil. R_f = 0.69 (hexanes/EtOAc, 3:1). ^{1}H NMR (300 MHz, $CDCl_3$) δ 0.88 (t, ^3J = 6.4 Hz, 3H, Me), 1.20−1.45 [m, 14H, Me(CH_2)$_7$], 1.77 (tt, ^3J = 7.1 Hz, ^3J = 6.5 Hz, 2H, 2'-H_2), 4.31 (t, ^3J = 6.5 Hz, 2H, 1'-H_2), 7.44 (t, ^3J = 7.5 Hz, 2H, Ph), 7.55 (t, ^3J = 7.2 Hz, 1H, Ph), 8.05 (d, ^3J = 7.3 Hz, 2H, Ph). The spectrum is in full agreement with published data.[8]

1-Decyl acrylate (10). 1-Decanol (**6**) and acrylonitrile were reacted according to GP 1. Chromatography (hexanes/EtOAc, 9:1) afforded **10** (47 mg, 29%) as a colourless oil. A similar reaction performed according to GP 2 yielded **10** (64 mg, 40%). R_f = 0.69 (hexanes/EtOAc, 3:1). ^{1}H NMR (300 MHz, $CDCl_3$) δ 0.88 (t, ^3J = 6.6 Hz, 3H, Me), 1.23–1.40 [m, 14H, H_3C(CH_2)$_7$], 1.66 (tt, ^3J = 7.1 Hz, ^3J = 6.7 Hz, 2H, 2'-H_2), 4.15 (t, ^3J = 6.7 Hz, 2H, 1'-H_2), 5.81 (dd, ^2J = 1.6 Hz, ^3J = 10.4 Hz, 1H, 3-H_{trans}), 6.12 (dd, ^3J = 10.4 Hz, ^3J = 17.3 Hz, 1H, 2'-H_2), 6.40 (dd, ^2J = 1.6 Hz, ^3J = 17.3 Hz, 1H, 3-H_{cis}). The spectrum is in full agreement with published data.[9]

6-Chlorohexyl acetate (12). 6-Chlorohexanol (**11**) and MeCN were reacted according to GP 1. Chromatography (hexanes/EtOAc, 9:1) afforded **12** (114 mg, 84%) as a colourless oil. R_f = 0.56 (hexanes/EtOAc, 3:1). ^{1}H NMR (300 MHz, $CDCl_3$) δ 1.31–1.53 (m, 4H, 3'-H_2, 4'-H_2), 1.64 (tt, ^3J = 6.8 Hz, ^3J = 7.2 Hz, 2H, 2'-H_2), 1.78 (tt, ^3J = 6.7 Hz, ^3J = 7.3 Hz, 2H, 5'-H_2), 2.05 (s, 3H, Me), 3.53 (t, ^3J = 6.7 Hz, 2H, 6'-H_2), 4.06 (t, ^3J = 6.7 Hz, 2H, 1'-H_2). The spectrum is in full agreement with published data.[10]

---

6 Magens, S.; Plietker, B. *J. Org. Chem.*, **2010**, *75*, 3715–3721.
7 Velusamy, S.; Borpuzari, S.; Punniyamurthy, T. *Tetrahedron*, **2005**, *61*, 2011–2015.
8 Behloul, C.; Guijarro, D.; Yus, M. *Synthesis*, **2006**, 309–314.
9 Ryu, J.-H.; Roy, R.; Ventura, J. *Langmuir*, **2010**, *26*, 7086–7092.
10 Doláková, P.; Dračínský, M.; Fanfrlík, J.; Holý, A. *Eur. J. Org. Chem.*, **2009**, 1082–1092.

6-Chlorohexyl phenylacetate (13). 6-Chlorohexanol (**11**) and BnCN were reacted according to GP 3. Chromatography (hexanes/EtOAc, 9:1) afforded **13** (174 mg, 90%) as a yellowish oil. R_f = 0.59 (hexanes/EtOAc, 3:1). ^{1}H NMR (300 MHz, $CDCl_3$) δ 1.23–1.49 (m, 4H, 3'-H_2, 4'-H_2), 1.63 (tt, ^3J = 6.8 Hz, ^3J = 7.3 Hz, 2H, 5'-H_2), 1.75 (tt, ^3J = 6.8 Hz, ^3J =7.3 Hz, 2H, 2'-H_2), 3.51 (t, ^3J = 6.7 Hz, 2H, 6'-H_2), 3.62 (s, 2H, CH_2Ph), 4.09 (t, ^3J = 6.6 Hz, 2H, 1'-H_2), 7.23–7.40 (m, 5H, Ph). The spectrum is in full agreement with published data.[11]

6-Chlorohexyl benzoate (14). 6-Chlorohexanol (**11**) and PhCN were reacted according to GP 3. Chromatography (hexanes/EtOAc, 9:1) afforded **14** (50 mg, 27%) as a yellowish oil. R_f = 0.62 (hexanes/EtOAc, 3:1). ^{1}H NMR (300 MHz, $CDCl_3$) δ 1.42–1.60 (m, 4H, 3'-H_2, 4'-H_2), 1.73–1.88 (m, 4H, 2'-H_2, 5'-H_2), 3.55 (t, ^3J = 6.6 Hz, 2H, 6'-H_2), 4.33 (t, ^3J = 6.6 Hz, 2H, 1'-H_2), 7.44 (t, ^3J = 7.5 Hz, 2H, Ph), 7.56 (t, ^3J = 7.4 Hz, 1H, Ph), 8.04 (d, ^3J = 7.8 Hz, 2H, Ph). The spectrum is in full agreement with published data.[12]

6-Chlorohexyl acrylate (15). 6-Chlorohexanol (**11**) and acrylonitrile were reacted according to GP 1. Chromatography (hexanes/EtOAc, 9:1) afforded **15** (55 mg, 38%) as a colourless oil. A similar reaction performed according to GP 2 yielded **15** (23 mg, 16%). R_f = 0.64 (hexanes/EtOAc, 3:1). ^{1}H NMR (300 MHz, $CDCl_3$) δ 1.33–1.55 (m, 4H, 3'-H_2, 4'-H_2), 1.69 (tt, ^3J = 6.7 Hz, ^3J = 7.2 Hz, 2H, 5'-H_2), 1.79 (tt, ^3J = 6.6 Hz, ^3J = 7.2 Hz, 2H, 2'-H_2), 3.54 (t, ^3J = 6.6 Hz, 2H, 6'-H_2), 4.16 (t, ^3J = 6.6 Hz, 2H, 1'-H_2), 5.82 (dd, ^2J = 1.1 Hz, ^3J = 10.4 Hz, 1H, 3-H_{trans}), 6.12 (dd, ^3J = 10.4 Hz, ^3J = 17.3 Hz, 1H, 2-H), 6.40 (dd, ^3J = 1.1 Hz, ^3J = 17.3 Hz, 1H, 3-H_{cis}). The spectrum is in full agreement with published data.[13]

2-(2-Ethoxyethoxy)ethyl acetate (17). 2-(2-Ethoxyethoxy)ethanol (**16**) and MeCN were reacted according to GP 1. Chromatography (hexanes/EtOAc, 4:1) afforded **17** (101 mg, 75%) as a colourless oil. R_f = 0.16 (hexanes/EtOAc 3:1). ^{1}H NMR (300 MHz, $CDCl_3$) δ 1.21 (t, ^3J = 7.0 Hz, 3H, OCH_2CH_3), 2.07 (s, 3H, OAc), 3.53 (q, ^3J = 7.0 Hz, 2H, OCH_2CH_3), 3.56–3.67 (m, 4H, $EtOCH_2CH_2O$), 3.70 (t, ^3J = 4.8 Hz, 2H, OCH_2CH_2OAc), 4.23 (t, ^3J = 4.8 Hz, 2H, OCH_2CH_2OAc). The spectrum is in full agreement with published data.[14]

---

[11] Bron, J; Sterk, G., J.; Timmerman, H.; Veerman, M. E. J.; Van Der Werf, J. F. *Substituted ethanolamine esters*, WO9408945 (A1), 28.04.1994.

[12] Molander, G. A.; Cavalcanti, L. N. *J. Org. Chem*., **2011**, *76*, 7195–7203.

[13] Bergbreiter, D. E.; Chance, B. S. *Macromolecules*, **2007**, *40*, 5337–5343.

[14] Oriyama, T.; Kimura, M.; Oda, M.; Koga, G. *Synlett*, **1993**, 437–440.

2-(2-Ethoxyethoxy)ethyl phenylacetate (18). 2-(2-Ethoxyethoxy)ethanol (**16**) and BnCN were reacted according to GP 3. Chromatography (hexanes/EtOAc, 4:1) afforded **18** (162 mg, 85%) as a colourless oil. R_f = 0.20 (hexanes/EtOAc, 3:1). IR (KBr): $\tilde{\nu}$ (cm^{-1}) = 3450 (w), 2974 (m), 2870 (m), 1737 (m, C=O), 1454 (w), 1253 (m), 1114 (m), 1043 (w), 697 (w). ^1H NMR (400 MHz, $CDCl_3$) δ 1.21 (t, ^3J = 7.0 Hz, 3H, Me), 3.52 (q, ^3J = 7.0 Hz, 2H, CH_2Me), 3.53–3.62 (m, 4H, EtOCH_2CH_2O), 3.65 (s, 2H, CH_2Ph), 3.69 (t, ^3J = 4.8 Hz, 2H, OCH_2CH$_2$OAc), 4.26 (t, ^3J = 4.8 Hz, 2H, OCH$_2$$CH_2$OAc), 7.23–7.35 (m, 5H, Ph). ^{13}C NMR (100 MHz, $CDCl_3$) δ 15.3 (q), 41.4 (t), 64.2 (t), 66.8 (t), 69.2 (t), 69.9 (t), 70.8 (t), 127.2 (d), 128.7 (2 d), 129.4 (2 d), 134.1 (s), 171.7 (s). MS (EI, 20 °C): *m/z* (%) = 252 (36) [M^+], 163 (34), 119 (15), 118 (59), 92 (10), 91 (100), 73 (14), 72 (35), 59 (22), 45 (26). HRMS ($^{12}C_{14}{}^{1}H_{20}{}^{16}O_4$, EI): calcd. 252.1362 amu; found 252.1364 amu. Anal. calcd for $C_{14}H_{20}O_4$ (252.14): C 66.65, H 7.99; found C 66.79, H 7.89.

2-(2-Ethoxyethoxy)ethyl benzoate (19). 2-(2-Ethoxyethoxy)ethanol (**16**) and PhCN were reacted according to GP 3. Chromatography (hexanes/EtOAc, 4:1) afforded **19** (47 mg, 26%) as a colourless oil. R_f = 0.23 (hexane/EtOAc, 3:1). ^1H NMR (300 MHz, $CDCl_3$) δ 1.20 (t, ^3J = 7.0 Hz, 3H, Me), 3.53 (q, ^3J = 7.0 Hz, 2H, CH_2Me), 3.58–3.73 (m, 4H, EtOCH_2CH_2O), 3.85 (t, ^3J = 4.8 Hz, 2H, OCH_2CH$_2$OAc), 4.49 (t, ^3J = 4.8 Hz, 2H, OCH$_2$$CH_2$OAc), 7.43 (t, ^3J = 7.5 Hz, 2H, Ph), 7.56 (t, ^3J = 7.4 Hz, 1H, Ph), 8.06 (d, ^3J = 7.7 Hz, 2H, Ph). The spectrum is in full agreement with published data.[15]

2-(2-Ethoxyethoxy)ethyl acrylate (20). 2-(2-Ethoxyethoxy)ethanol (**16**) and acrylonitrile were reacted according to GP 1. Chromatography (hexanes/EtOAc, 4:1) afforded **20** (32 mg, 23%) as a colourless oil. A similar reaction performed according to GP 2 yielded **20** (27 mg, 19%). R_f = 0.27 (hexanes/EtOAc, 3:1). ^1H NMR (300 MHz, $CDCl_3$) δ 1.21 (t, ^3J = 7.0 Hz, 3H, Me), 3.53 (q, ^3J = 7.0 Hz, 2H, CH_2Me), 3.57–3.69 (m, 4H, EtOCH_2CH_2O), 3.75 (t, ^3J = 4.8 Hz, 2H, OCH_2CH$_2$OAc), 4.32 (t, ^3J = 4.8 Hz, 2H, OCH$_2$$CH_2$OAc), 5.83 (dd, ^2J = 1.3 Hz, ^3J = 10.4, 1H, 3-H_{trans}), 6.15 (dd, ^3J = 10.4 Hz, ^3J = 17.3, 1H, 2-H), 6.43 (dd, ^2J = 1.3 Hz, ^3J = 17.3 Hz, 1H, 3-H_{cis}). The spectrum is in full agreement with published data.[16]

4-Nitrobenzyl acetate (22). 4-Nitrobenzyl alcohol (**21**) and MeCN were reacted according to GP 1. Chromatography (hexanes/EtOAc, 4:1) afforded **22** (133 mg, 90%) as a yellowish solid. R_f = 0.36 (hexanes/EtOAc, 3:1). ^1H NMR (300 MHz, $CDCl_3$) δ 2.15 (s, 3H, Me), 5.20 (s, 2H, CH_2), 7.52 (d, ^3J = 8.8 Hz, 2H, Ar), 8.22 (d, ^3J = 8.8 Hz, 2H, Ar). The spectrum is in full agreement with published data.[17]

---

[15] Conn, R. C.; Collett, A. R.; Lazzell, C. L. *J. Am. Chem. Soc.*, **1932**, *54*, 4370–4372.
[16] Philippon, A.; Degueil-Castaing, M.; Beckwith, A. L. J.; Maillard, B., *J. Org. Chem.*, **1998,** *63*, 6814–6819.
[17] Barbero, M.; Bazzi, S.; Cadamuro, S.; Dughera, S. *Eur. J. Org. Chem.*, **2009**, 430–436.

4-Nitrobenzyl phenylacetate (23). 4-Nitrobenzyl alcohol (**21**) and BnCN were reacted according to GP 3. Chromatography (hexanes/EtOAc, 9:1) afforded **23** (160 mg, 78%) as a white solid. R_f = 0.39 (hexanes/EtOAc, 3:1). ^{1}H NMR (300 MHz, $CDCl_3$) δ 3.72 (s, 2H, PhCH_2), 5.22 (s, 2H, OCH_2), 7.24–7.38 (m, 5H, Ph), 7.42 (d, ^3J = 8.7 Hz, 2H, Ar), 8.18 (d, ^3J = 8.7 Hz, 2H, Ar). The spectrum is in full agreement with published data.[18]

4-Nitrobenzyl benzoate (24). 4-Nitrobenzyl alcohol (**21**) and PhCN were reacted according to GP 3. Chromatography (hexanes/EtOAc, 9:1) afforded **24** (77 mg, 39%) as a white solid. R_f = 0.39 (hexanes/EtOAc, 3:1). ^{1}H NMR (300 MHz, $CDCl_3$) δ 5.46 (s, 2H, CH_2), 7.47 (t, ^3J = 7.6 Hz, 2H, Ph), 7.60 (t, ^3J = 7.0 Hz, 1H, Ph), 7.61 (d, ^3J = 8.6 Hz, 2H, Ar), 8.09 (d, ^3J = 7.9 Hz, 2H, Ph), 8.25 (d, ^3J = 8.6 Hz, 2H, Ar). The spectrum is in full agreement with published data.[19]

4-Nitrobenzyl acrylate (25). 4-Nitrobenzyl alcohol (**21**) and acrylonitrile were reacted according to GP 1. Chromatography (hexanes/EtOAc, 9:1) afforded **25** (133 mg, 85%) as a yellowish solid. R_f = 0.36 (hexanes/EtOAc, 3:1). ^{1}H NMR (300 MHz, $CDCl_3$) δ 5.30 (s, 2H, CH_2Ar), 5.92 (d, ^3J = 10.4 Hz, 1H, 3-H_{trans}), 6.20 (dd, ^3J = 10.4 Hz, ^3J = 17.3 Hz, 1H, 2-H), 6.49 (d, ^3J = 17.3 Hz, 1H, 3-H_{cis}), 7.54 (d, ^3J = 8.6 Hz, 2H, Ar), 8.23 (d, ^3J = 8.6 Hz, 2H, Ar). The spectrum is in full agreement with published data.[20]

4-(Acetoxymethyl)benzoic acid (27). 4-(Hydroxymethyl)benzoic acid (**26**) and MeCN were reacted according to GP 1. Chromatography (CH_2Cl_2/MeOH/AcOH, 100:10:1) afforded **27** (130 mg, 88%) as a white solid. R_f = 0.31 (MeOH/CH_2Cl_2, 19:1). ^{1}H NMR (300 MHz, $CDCl_3$) δ 2.15 (s, 3H, Me), 5.18 (s, 2H, CH_2), 7.46 (d, ^3J = 8.2 Hz, 2H, Ar), 8.12 (d, ^3J = 8.2 Hz, 2H, Ar), 11.0 (bs, 1H, CO_2H). The spectrum is in full agreement with published data.[21]

4-[(Phenylacetoxy)methyl]benzoic acid (28). 4-(Hydroxymethyl)benzoic acid (**26**) and BnCN were reacted according to GP 3. Chromatography (CH_2Cl_2/MeOH/AcOH, 100:10:1) afforded **28** (179 mg, 87%) as a white solid. R_f = 0.29 (MeOH/CH_2Cl_2, 19:1). IR (ATR): $\tilde{\nu}$ (cm^{-1}) = 2943 (m), 2557 (m), 1719 (s, C=O), 1682 (s, C=O), 1613 (m), 1427 (m), 1291 (s), 1266 (s), 1144 (s), 1127 (s), 1029 (m), 750 (s), 718 (s), 692 (s). ^{1}H NMR (400 MHz, $CDCl_3$) δ 3.71 (s, 2H, PhCH_2), 5.21 (s, 2H, OCH_2), 7.27–7.37 (m, 5H, Ph), 7.38 (d, ^3J = 8.4 Hz, 2H, Ar), 8.08 (d, ^3J = 8.4 Hz, 2H, Ar), 11.1 (bs, 1H, COOH). ^{13}C NMR (100 MHz, $CDCl_3$) δ 41.5 (t), 65.9 (t), 127.4 (d), 127.7 (d), 128.8 (d), 129.1 (s), 129.4 (d), 130.6 (d),

[18] Merkley, N.; Warkentin, J. *Can. J. Chem*,. **2000**, *78*, 942–949.
[19] Sharghi, H; Sarvari, M. H.; Eskandari, R. *J. Chem. Res.*, **2005**, 488–491.
[20] Merski, M; Townsend, C. A. *J. Am. Chem. Soc.*, **2007**, *129*, 15750–15751.
[21] Gigante, F.; Kaiser, M.; Brun, R.; Gilbert, I. H. *Bioorg. Med. Chem.*, **2010**, *18*, 7291–7301.

133.8 (s), 142.0 (s), 171.4 (s), 171.4 (s). MS (EI, 110 °C): *m*/*z* (%) = 270 (6) [M^+], 136 (4), 135 (35), 107 (25), 92 (10), 91 (100), 90 (11), 89 (9), 79 (4), 77 (9), 65 (14). HRMS ($^{12}C_{16}{}^{1}H_{14}{}^{16}O_4$, EI): calcd. 270.0892 amu; found 270.0893 amu. $C_{16}H_{14}O_4$ (270.09): calcd. C 71.10, H 5.22; found C 70.72, H 5.02.

4-(Benzoyloxymethyl)benzoic acid (29). 4-(Hydroxymethyl)benzoic acid (**26**) and PhCN were reacted according to GP 3. Chromatography (CH_2Cl_2/MeOH/AcOH, 100:10:1) afforded **29** (60 mg, 31%) as a white solid. R_f = 0.31 (MeOH/CH_2Cl_2, 19:1) IR (ATR): $\tilde{\nu}$ (cm^{-1}) = 2955 (w), 2660 (w), 2540 (w), 1715 (w, C=O), 1677 (w, C=O), 1425 (w), 1264 (m), 1095 (w), 1069 (w), 935 (w), 756 (w), 699 (w). ^{1}H NMR (400 MHz, $CDCl_3$) δ 5.45 (s, 2H, CH_2), 7.47 (t, ^3J = 7.7 Hz, 2H, Ph), 7.55 (d, ^3J = 8.3 Hz, 2H, Ar), 7.59 (t, ^3J = 7.4 Hz, ^4J = 1.5 Hz, 1H, Ph), 8.10 (dd, ^3J = 8.4 Hz, ^4J = 1.4 Hz, 2H, Ph), 8.14 (d, ^3J = 8.3 Hz, 2H, Ar),10,8 (bs, 1H, COOH). ^{13}C NMR (100 MHz, $CDCl_3$) δ 66.0 (t), 127.8 (d), 128.6 (d), 129.2 (s), 129.9 (d), 130.7 (d), 133.4 (d), 142.2 (s), 166.4 (s), 171.9 (s), one signal (s) covered. MS (EI, 100 °C): *m*/*z* (%) = 257 (6), 256 (42) [M^+], 135 (30), 121 (10), 107 (26), 106 (13), 105 (100), 90 (7), 89 (10), 79 (9), 78 (6), 77 (52), 51 (15), 43 (13). HRMS ($^{12}C_{15}{}^{1}H_{12}{}^{16}O_4$, EI): calcd. 256.0736 amu; found 256.0734 amu.

4-(Acryloyloxymethyl)benzoic acid (30). 4-(Hydroxymethyl)benzoic acid (**26**) and acrylonitrile were reacted according to GP 1. Chromatography (CH_2Cl_2/MeOH/AcOH, 100:10:1) afforded **30** (98 mg, 64%) as a white solid. R_f = 0.27 (MeOH/CH_2Cl_2, 19:1). ^{1}H NMR (400 MHz, $CDCl_3$) δ 5.28 (s, 2H, OCH_2), 5.90 (d, ^3J = 10.4 Hz, 1H, 3-H_{cis}), 6.20 (dd, ^3J = 10.4 Hz, ^3J = 17.3 Hz, 1H, 2-H), 6.49 (d, ^3J = 17.3 Hz, 1H, 3-H_{trans}), 7.48 (d, ^3J = 7.8 Hz, 2H, Ar), 8.12 (d, ^3J = 7.8 Hz, 2H, Ar), 11.4 (bs, 1H, COOH). ^{13}C NMR (100 MHz, $CDCl_3$) δ 65.6 (t), 127.9 (d), 128.1 (d), 129.2 (s), 130.7 (d), 131.8 (t), 142.1 (s), 166.0 (s), 171.6 (s).

Hexane-1,6-diyl diacetate (32) and 6-hydroxyhexyl acetate (33). 1,6-Hexanediol (**31**) and MeCN were reacted according to GP 1 with 4 equivalents of TMSOTf. Chromatography (hexanes/EtOAc, 9:1) afforded **32** (48 mg, 32%) as a colourless oil. R_f = 0.45 (hexanes/EtOAc, 3:1). ^{1}H NMR (300 MHz, $CDCl_3$) δ 1.33–1.42 (m, 4H, 3'-H_2, 4'-H_2), 1.56–1.68 (m, 4H, 2'-H_2, 5'-H_2), 2.04 (s, 6H, Me), 4.05 (t, ^3J = 6.7 Hz, 4H, 1'-H_2, 6'-H_2). The spectrum is in full agreement with published data.[22] **Side product: 6-Hydroxyhexyl acetate (33).** Chromatography (hexanes/EtOAc, 9:1) afforded **33** (22 mg, 19%) as a colourless oil. R_f = 0.29 (hexanes/EtOAc, 1:1). ^{1}H NMR (300 MHz, $CDCl_3$) δ 1.36–1.45 (m, 4H, 3'-H_2, 4'-H_2), 1.53–1.71 (m, 4H, 2'-H_2, 5'-H_2), 1.90 (br s, 1H, OH), 2.05 (s, 3H, OAc), 3.65 (t, ^3J = 6.5 Hz, 2H, 6'-H_2), 4.06 (t, ^3J = 6.7 Hz, 2H, 1'-H_2). The spectrum is in full agreement with published data.[23]

---

[22] Miyamoto, K.; Tada, N.; Ochiai, M. *J. Am. Chem. Soc.*, **2007**, 2772–2773.
[23] Rawal, G. K.; Rani, S.; Kumar, A.; Vankar, Y. D. *Tetrahedron Lett.*, **2006**, *47*, 9117–9120.

Hexane-1,6-diyl bis(phenylacetate) (34) and 6-hydroxyhexyl phenylacetate (35). 1,6 Hexanediol (**31**) and BnCN were reacted according to GP 3 with 4 equivalents of TMSOTf. Chromatography (hexanes/EtOAc, 9:1) afforded **34** (122 mg, 46%) as a colourless oil. R_f = 0.45 (hexanes/EtOAc, 3:1). ^{1}H NMR (300 MHz, $CDCl_3$) δ 1.24–1.36 (m, 4H, 3'-H_2, 4'-H_2), 1.52–1.64 (m, 4H, 2'-H_2, 5'-H_2), 3.61 (s, 4H, CH_2Ph), 4.06 (t, ^3J = 6.6 Hz, 2H, 1'-H_2, 6'-H_2), 7.22–7.36 (m, 10H, Ph). The spectrum is in full agreement with published data.[24] **Side product: 6-Hydroxyhexyl phenylacetate (35):** Chromatography (hexanes/EtOAc, 9:1) afforded **35** (66 mg, 37%) as a colourless oil. R_f = 0.29 (hexanes/EtOAc, 1:1). ^{1}H NMR (300 MHz, $CDCl_3$) δ 1.29–1.40 (m, 4H, 3'-H_2, 4'-H_2), 1.49–1.58 (m, 2H, 5'-H_2), 1.63 (tt, ^3J = 6.9 Hz, ^3J = 6.8 Hz, 2H, 2'-H_2), 3.61 (s, 2H, CH_2Ph), 3.62 (t, ^3J = 6.5 Hz, 2H, 6'-H_2), 4.09 (t, ^3J = 6.6 Hz, 2H, 1'-H_2), 7.23−7.36 (m, 5H, Ph). The spectrum is in full agreement with published data.[25]

Ethyl 6-acetoxyhexanoate (37). Ethyl 6-hydroxyhexanoate (**36**) and MeCN were reacted according to GP 1. Chromatography (hexanes/EtOAc, 9:1) afforded **37** (32 mg, 21%) as a colourless oil. R_f = 0.47 (hexanes/EtOAc, 3:1). ^{1}H NMR (300 MHz, $CDCl_3$) δ 1.25 (t, ^3J = 7.1 Hz, 3H, CH_2CH_3), 1.32–1.44 (m, 2H, 4-H_2), 1.59–1.71 (m, 4H, 3-H_2, 5-H_2), 2.04 (s, 3H, OAc), 2.30 (t, ^3J = 7.5 Hz, 2H, 2-H_2), 4.05 (t, ^3J = 6.6 Hz, 2H, 6-H_2), 4.12 (q, ^3J = 7.1 Hz, 2H, CH_2CH_3). The spectrum is in full agreement with published data.[26]

Ethyl 6-(phenylacetoxy)hexanoate (38). Ethyl 6-hydroxyhexanoate (**36**) and BnCN were reacted according to GP 3. Chromatography (hexanes/EtOAc, 9:1) afforded **38** (33 mg, 16%) as a yellowish oil. R_f = 0.44 (hexanes/EtOAc, 3:1). IR (KBr): $\tilde{\nu}$ (cm^{-1}) = 3444 (w), 2940 (m), 1733 (s, C=O), 1455 (w), 1249 (m), 1110 (s), 1031 (m), 698 (w), 617 (m). ^{1}H NMR (400 MHz, $CDCl_3$) δ 1.25 (t, ^3J = 7.1 Hz, 3H, Me), 1.29–1.39 (m, 2H, 4-H_2), 1.58−1.67 (m, 4H, 3-H_2, 5-H_2), 2.27 (t, ^3J = 7.5 Hz, 2H, 2-H_2), 3.61 (s, 2H, CH_2Ph), 4.08 (t, ^3J = 6.6 Hz, 2H, 6-H_2), 4.12 (q, ^3J = 7.1 Hz, 2H, CH_2CH_3), 7.23–7.35 (m, 5H, Ph). ^{13}C NMR (100 MHz, $CDCl_3$) δ 14.4 (q), 24.7 (t), 25.6 (t), 28.4 (t), 34.3 (t), 41.6 (t), 60.4 (t), 64.8 (t), 127.2 (d), 128.7 (2 d), 129.4 (2 d), 134.3 (s), 171.8 (s), 173.7 (s). MS (EI, 40 °C): m/z (%) = 278 (15) [M^+], 118 (100), 115 (15), 91 (60), 69 (16). HRMS ($^{12}C_{16}{}^1H_{22}{}^{16}O_4$, EI): calcd. 278.1518 amu; found 278.1516. amu.

6-Ethoxy-6-oxohexyl benzoate (39). Ethyl 6-hydroxyhexanoate (**36**) and PhCN were reacted according to GP 3. Chromatography (hexanes/EtOAc, 9:1) afforded **39** (21 mg, 10%) as a yellowish oil. R_f = 0.44 (hexanes/EtOAc, 3:1). IR (KBr): $\tilde{\nu}$ (cm^{-1}) = 3440 (w), 2940 (w), 1721 (m, C=O), 1275 (m), 1113 (s),

---

24 Twibanire, J.-d'A. K.; Grindley, T. B. *Org. Lett.*, **2011**, *13*, 2988–2991.
25 Tabenkin, B.; Lehr, H.; Wayman, A. C.; Goldberg, M. W. *Arch. Biochem. Biophys.*, **1952**, *38*, 43–48.
26 Yamashita M.; Takemoto, Y.; Ihara, E.; Yasuda, H. *Macromolecules*, **1996**, *29*, 1798–1806.

713 (m), 618 (m). ^{1}H NMR (400 MHz, $CDCl_3$) δ 1.25 (t, ^3J = 7.1 Hz, 3H, CH_2*CH_3*), 1.44–1.54 (m, 2H, 4'-H_2), 1.71 (tt, ^3J = 7.5 Hz, ^3J = 7.7 Hz, 2H, 3'-H_2), 1.79 (tt, ^3J = 6.6 Hz, ^3J = 7.6 Hz, 2H, 5'-H_2), 2.33 (t, ^3J = 7.5 Hz, 2H, 2'-H_2), 4.12 (q, ^3J = 7.1 Hz, 2H, *CH_2*CH_3), 4.32 (t, ^3J = 6.6 Hz, 2H, 6'-H_2), 7.44 (t, ^3J = 7.7 Hz, 2H, Ph), 7.55 (t, ^3J = 7.4 Hz, 1H, Ph), 8.04 (d, ^3J = 7.9 Hz, 2H, Ph). ^{13}C NMR (100 MHz, $CDCl_3$) δ 14.4 (q), 24.8 (t), 25.8 (t), 28.6 (t), 34.4 (t), 60.4 (t), 64.9 (t), 128.5 (2 d), 129.7 (2 d), 130.5 (s), 133.0 (d), 166.8 (s), 173.7 (s). MS (EI, 40 °C): *m*/*z* (%) = 264 (8) [M^+], 142 (49), 105 (100), 88 (12), 77 (14). HRMS ($^{12}C_{15}{}^1H_{20}{}^{16}O_4$, EI): calcd. 264.1362 amu; found 264.1360 amu.

Ethyl 6-(acryloyloxy)hexanoate (40). Ethyl 6-hydroxyhexanoate (**36**) and acrylonitrile were reacted according to GP 1. Chromatography (hexanes/EtOAc, 9:1) afforded **40** (12 mg, 7%) as a colourless oil. R_f = 0.38 (hexanes/EtOAc, 3:1). IR (KBr): $\tilde{\nu}$ (cm^{-1}) = 3439 (w), 2942 (m), 1729 (s, C=O), 1409 (m), 1192 (m), 811 (w), 618 (w). ^{1}H NMR (400 MHz, $CDCl_3$) δ 1.25 (t, ^3J = 7.1 Hz, 3H, CH_2*CH_3*), 1.36–1.45 (m, 2H, 4-H_2), 1.61–1.73 (m, 4H, 3-H_2, 5-H_2), 2.31 (t, ^3J = 7.5 Hz, 2H, 2-H_2), 4.12 (q, ^3J = 7.1 Hz, 2H, *CH_2*CH_3), 4.15 (t, ^3J = 6.7 Hz, 2H, 6-H_2), 5.81 (dd, ^3J = 10.4 Hz, ^2J = 1.3 Hz, 1H, 3'-H_{trans}), 6.11 (dd, ^3J = 17.3 Hz, ^3J = 10.4 Hz, 1H, 2'-H_α), 6.39 (dd, ^3J = 17.3 Hz, ^2J = 1.3 Hz, 1H, 3'-H_{cis}). ^{13}C NMR (100 MHz, $CDCl_3$) δ 14.4 (q), 24.7 (t), 25.7 (t), 28.4 (t), 34.3 (t), 60.4 (t), 64.5 (t), 128.7 (d), 130.7 (t), 166.4 (s), 173.7 (s). MS (EI, 30 °C): *m*/*z* (%) = 214 (1) [M^+], 169 (21) [$M^+ - OCH_2CH_3$], 142 (58), 114 (11), 113 (13), 88 (25), 73 (14), 69 (16), 68 (26), 55 (100) [$OCHCH_2$]. HRMS ($^{12}C_{11}{}^1H_{18}{}^{16}O_4$, EI): calcd. 214.1205 amu; found 214.1208 amu.

4-(Benzyloxycarbonylamino)butyl acetate (42). Benzyl 4-hydroxybutylcarbamate (**41**) and MeCN were reacted according to GP 1. Chromatography (hexanes/EtOAc, 2:1) afforded **42** (27 mg, 13%) as a colourless oil. R_f = 0.55 (hexanes/EtOAc, 1:2). ^{1}H NMR (300 MHz, $CDCl_3$) δ 1.50–1.73 (m, 4H, 2'-H_2, 3'-H_2), 2.04 (s, 3H, OAc), 3.23 (q, ^3J = 6.4 Hz, 2H, 1'-H_2), 4.07 (t, ^3J = 6.1 Hz, 2H, 4'-H_2), 4.79 (s, 1H, NH), 5.10 (s, 2H, *CH_2*Ph), 7.28–7.39 (m, 5H, Ph). The spectrum is in full agreement with published data.[27]

4-(Benzyloxycarbonylamino)butyl phenylacetate (43). Benzyl 4-hydroxybutylcarbamate (**41**) and BnCN were reacted according to GP 3. Chromatography (hexanes/EtOAc, 3:1) afforded **43** (34 mg, 13%) as a yellowish oil. R_f = 0.71 (hexanes/EtOAc, 1:2). IR (KBr): $\tilde{\nu}$ (cm^{-1}) = 3347 (m), 2951 (m), 1727 (s, C=O), 1530 (m), 1455 (m), 1253 (s), 1141 (m), 1027 (m), 727 (m), 697 (m). ^{1}H NMR (400 MHz, $CDCl_3$) δ 1.51 (tt, ^3J = 7.0 Hz, ^3J = 7.5 Hz, 2H, 2'-H_2), 1.64 (tt, ^3J = 7.4 Hz, ^3J = 6.5 Hz, 2H, 3'-H_2), 3.18 (q, ^3J = 6.6 Hz, 2H, 1'-H_2), 3.61 (s, 2H, C*CH_2*Ph), 4.10 (t, ^3J = 6.4 Hz, 2H, 4'-H_2), 4.72 (s,

[27] Kometani, M.; Ihara, K.; Kimura, R.; Kinoshita, H. *Bull., Chem. Soc. Jpn*. **2009**, *82*, 364–380.

1H, NH), 5.10 (s, 2H, O*CH*$_2$Ph), 7.23–7.40 (m, 10H, Ph). ^{13}C NMR (100 MHz, $CDCl_3$) δ 25.9 (t), 26.7 (t), 40.7 (t), 41.6 (t), 64.5 (t), 66.8 (t), 127.2 (2 d), 128.3 (2 d), 128.7 (2 d), 128.7 (2 d), 129.4 (2 d), 134.2 (s), 136.7 (s), 156.5 (s), 171.7 (s). MS (EI, 80 °C): *m*/*z* (%) = 341 (5) [M^+], 180 (12), 118 (11), 108 (64), 107 (23), 92 (15), 91 (100). HRMS ($^{12}C_{20}{}^{1}H_{23}{}^{14}N^{16}O_4$, EI): calcd. 341.1627 amu; found 341.1625 amu.

4-(Benzyloxycarbonylamino)butyl acrylate (44). Benzyl 4-hydroxybutylcarbamate (**41**) and acrylonitrile were reacted according to GP 1. Chromatography (hexanes/EtOAc, 1:1) afforded **44** (30 mg, 14%) as a yellowish oil. R_f = 0.62 (hexanes/EtOAc, 1:2). IR (KBr): $\tilde{\nu}$ (cm^{-1}) = 3346 (m), 2954 (m), 1723 (m, C=O), 1532 (m), 1454 (m), 1410 (m), 1256 (m), 1194 (m), 1135 (m), 1062 (w), 1027 (w), 985 (w), 812 (w), 739 (w), 698 (w). ^{1}H NMR (400 MHz, $CDCl_3$) δ 1.52–1.76 (m, 4H, 2'-H_2, 3'-H_2), 3.24 (q, ^3J = 6.6 Hz, 2H, 1'-H_2), 4.17 (t, ^3J = 6.3 Hz, 2H, 4'-H_2), 4.81 (s, 1H, NH), 5.09 (s, 2H, *CH*$_2$Ph), 5.82 (dd, ^3J = 10.4 Hz, ^2J = 1.4 Hz, 1H, 3-H_{trans}), 6.11 (dd, ^3J = 17.3 Hz, ^3J = 10.4 Hz, 1H, 2-H), 6.40 (dd, ^3J = 17.3 Hz, ^2J = 1.4 Hz, 1H, 3-H_{cis}), 7.28–7.40 (m, 5H, Ph). ^{13}C NMR (100 MHz, $CDCl_3$) δ 26.1 (t), 26.8 (t), 40.8 (t), 64.2 (t), 66.8 (t), 128.0 (2 d), 128.3 (d), 128.6 (d), 128.7 (2 d), 130.9 (t), 136.7 (s), 156.5 (s), 166.3 (s). MS (EI, 50 °C): *m*/*z* (%) = 277 (1) [M^+], 205 (51), 160 (10), 98 (14), 92 (12), 91 (100), 65 (9). HRMS ($^{12}C_{15}{}^{1}H_{19}{}^{14}N^{16}O_4$, EI): calcd. 277.1314 amu; found 277.1317 amu.

Cyclohexyl acetate (46). Cyclohexanol (**45**) and MeCN were reacted according to GP 1. Chromatography (hexanes/EtOAc, 9:1) afforded **46** (16 mg, 15%) as a colourless oil. R_f = 0.50 (hexanes/EtOAc, 3:1). ^{1}H NMR (300 MHz, $CDCl_3$) δ 1.19–1.42 (m, 5H, 3'-H_2, 4'-$H_a$$H_b$, 5'-$H_2$), 1.48–1.58 (m, 1H, 4'-$H_a$$H_b$), 1.65–1.76 (m, 2H, 2'-$H_2$ and/or 6'-H_2), 1.79–1.90 (m, 2H, 2'-H_2 and/or 6'-H_2), 2.02 (s, 3H, Me), 4.72 (tt, ^3J = 4.2 Hz, ^3J = 9.1 Hz, 1H, 1'-H). The spectrum is in full agreement with published data.[28]

Cyclohexyl phenylacetate (47). Cyclohexanol (**45**) and BnCN were reacted according to GP 3. Chromatography (hexanes/EtOAc, 9:1) afforded **47** (42 mg, 25%) as a colourless oil. R_f = 0.59 (hexanes/EtOAc, 3:1). ^{1}H NMR (300 MHz, $CDCl_3$) δ 1.19–1.56 (m, 6H, 3'-H_2, 4'-H_2, 5'-H_2), 1.61–1.71 (m, 2H, 2'-H_2 and/or 6'-H_2), 1.76–1.88 (m, 2H, 2'-H_2 and/or 6'-H_2), 3.60 (s, 2H, 2-H_2), 4.77 (tt, ^3J = 3.7 Hz, ^3J = 8.8 Hz, 1H, 1'-H), 7.22–7.36 (m, 5H, Ph). The spectrum is in full agreement with published data.[29]

2-(4-Hydroxyphenyl)ethyl acetate (54). 4-(2-Hydroxyethyl)phenol (**53**) and MeCN were reacted according to GP 3. Chromatography (hexanes/EtOAc, 4:1) afforded **54** (89 mg, 65%) as a yellowish oil.

---

[28] Débieux, J.-L.; Cosandey, A.; Helgen, C.; Bochet, C. G. *Eur. J. Org. Chem.*, **2007**, 2073–2077.
[29] Yang, C.-G.; He, C. *J. Am. Chem. Soc.*, **2005**, *127*, 6966–6967.

R_f = 0.24 (hexanes/EtOAc, 3:1). ^{1}H NMR (300 MHz, $CDCl_3$) δ 2.04 (s, 3H, Me), 2.86 (t, ^3J = 7.1 Hz, 2H, 2'-H_2), 4.24 (t, ^3J = 7.1 Hz, 2H, 1'-H_2), 6.77 (d, ^3J = 8.4 Hz, 2H, Ar), 7.08 (d, ^3J = 8.4 Hz, 2H, Ar). The spectrum is in full agreement with published data.[30]

2-(4-Hydroxyphenyl)ethyl phenylacetate, monaspilosin (**55**). 4-(2-Hydroxyethyl)phenol (**53**) and BnCN were reacted according to GP 3. Chromatography (hexanes/EtOAc, 4:1) afforded **55** (142 mg, 73%) as a colourless wax. R_f = 0.24 (hexanes/EtOAc, 3:1). IR (ATR): $\tilde{\nu}$ (cm^{-1}) = 3315 (m), 2955 (w), 1690 (s, C=O), 1594 (m), 1514 (m), 1452 (m), 1221 (s), 1147 (s), 1015 (s). ^{1}H NMR (400 MHz, $CDCl_3$) δ 2.84 (t, ^3J = 7.0 Hz, 2H, 2'-H_2), 3.61 (s, 2H, PhCH_2), 4.26 (t, ^3J = 7.0 Hz, 2H, 1'-H_2), 5.21 (s, 1H, OH), 6.72 (d, ^3J = 8.5 Hz, 2H, Ar), 6.99 (d, ^3J = 8.5 Hz, 2H, Ar), 7.22−7.35 (m, 5H, Ph). ^{13}C NMR (100 MHz, $CDCl_3$) δ 34.3 (t), 41.6 (t), 65.8 (t), 115.5 (2 d), 127.2 (d), 128.7 (2 d), 129.4 (2 d), 129.9 (s), 130.2 (2 d), 134.1 (s), 154.4 (s), 171.9 (s). MS (EI, 100 °C): *m*/*z* (%) = 256 (4) [M^+], 121 (40), 120 (100), 107 (49), 91 (72), 77 (16). HRMS ($^{12}C_{16}{}^1H_{16}{}^{16}O_3$, EI): calcd. 256.1099 amu; found 256.1101 amu. $C_{16}H_{16}O_3$ (256.11): calcd. C 74.98, H 6.29; found C 74.90, H 6.28.

Benzyl acetate (57) and *N*-benzylacetamide (58). Benzyl alcohol (**56**) and MeCN were reacted according to GP 1. Chromatography (hexanes/EtOAc, 9:1) afforded **57** (47 mg, 41%) as a colourless oil. R_f = 0.50 (hexanes/EtOAc, 3:1). ^{1}H NMR (300 MHz, $CDCl_3$) δ 2.11 (s, 3H, Me), 5.11 (s, 2H, CH_2), 7.28–7.40 (m, 5H, Ph). The spectrum is in full agreement with published data.[31] **Side product: *N*-Benzylacetamide (58):** Chromatography (hexanes/EtOAc, 1:3) afforded **58** (20 mg, 18%) as a white solid. R_f = 0.21 (hexanes/EtOAc, 1:2). ^{1}H NMR (300 MHz, $CDCl_3$) δ 2.00 (s, 3H, CH_3), 4.41 (d, ^3J = 5.7 Hz, 2H, CH_2), 5.97 (s, 1H, NH), 7.23–7.40 (m, 5H, Ph). The spectrum is in full agreement with published data.[32]

Benzyl phenylacetate (59) and *N*-benzyl-phenylacetamide (60). Benzyl alcohol (**56**) and BnCN were reacted according to GP 3. Chromatography (hexanes/EtOAc, 1:2) afforded amide **60** (100 mg, 59%) as a white solid. R_f = 0.71 (hexanes/EtOAc, 1:2). ^{1}H NMR (300 MHz, $CDCl_3$) δ 3.63 (s, 2H, PhCH_2CO), 4.41 (d, ^3J = 5.8 Hz, 2H, NHCH_2Ph), 5.71 (s, 1H, NH), 7.18 (d, ^3J = 6.5 Hz, 2H, Ph), 7.21–7.40 (m, 8H, Ph). The spectrum is in full agreement with published data.[33] **Side product: Benzyl phenylacetate (59):** Chromatography (hexanes/EtOAc, 9:1) afforded **59** (30 mg, 18%) as a yellowish oil. R_f = 0.53

30 Seidel, G.; Laurich, D.; Fürstner, A. *J. Org. Chem.*, **2004**, *69*, 3950–3952.
31 Débieux, J.-L.; Cosandey, A.; Helgen, C.; Bochet, C. G. *Eur. J. Org. Chem.*, **2007**, 2073–2077.
32 Bia, N.-M.; Rena, M.-G.; Song, Q.-H. *Synth. Commun.*, **2010**, *40*, 2617–2623.
33 Chen, Z.-W.; Jiang, H.-F.; Pan, X.-Y., He, Z.-J. *Tetrahedron*, **2011**, *67*, 5920–5927.

(hexanes/EtOAc, 3:1). ^{1}H NMR (300 MHz, $CDCl_3$) δ 3.68 (s, 2H, Ph*CH₂*CO), 5.14 (s, 2H, OCH_2), 7.19–7.45 (m, 10H, Ph). The spectrum is in full agreement with published data.[34]

***N*-Benzylbenzamide** (**61**). Benzyl alcohol (**56**) and PhCN were reacted according to GP 3. Chromatography (hexanes/EtOAc, 1:2) afforded **61** (105 mg, 66%) as a white solid. R_f = 0.70 (hexanes/EtOAc, 1:2). ^{1}H NMR (300 MHz, $CDCl_3$) δ 4.66 (d, ^3J = 5.6 Hz, 2H, CH_2), 6.39 (s, 1H, NH), 7.28–7.53 (m, 8H, Ph), 7.79 (d, ^3J = 7.6 Hz, 2H, Ph). The spectrum is in full agreement with published data.[35]

Benzyl acrylate (62) and *N*-benzylacrylamide (63). Benzyl alcohol (**56**) and acrylonitrile were reacted according to GP 1. Chromatography (hexanes/EtOAc, 1:1) afforded **63** (110 mg, 90%) as a white solid. R_f = 0.40 (hexanes/EtOAc, 1:2). ^{1}H NMR (300 MHz, $CDCl_3$) δ 4.52 (d, ^3J = 5.8 Hz, 2H, NHCH_2Ph), 5.67 (dd, ^3J = 10.2 Hz, ^2J = 1.4 Hz, 1H, 3-H_{trans}), 5.93 (s, 1H, NH), 6.11 (dd, ^3J = 17.0 Hz, 10.2 Hz, 1H, 2-H), 6.33 (dd, ^3J = 17.0 Hz, ^2J = 1.4 Hz), 7.27−7.38 (m, 5H, Ph). The spectrum is in full agreement with published data.[36] **Side product: Benzyl acrylate (62):** Chromatography (hexanes/EtOAc, 1:1) afforded **62** (5 mg, 4%) as a colourless oil. R_f = 0.60 (hexanes/EtOAc, 3:1). ^{1}H NMR (300 MHz, $CDCl_3$) δ 5.20 (s, 2H, CH_2), 5.85 (dd, ^2J = 1.5 Hz, ^3J = 10.4 Hz, 1H, 3-H_{trans}), 6.17 (dd, ^3J = 10.4 Hz, ^3J = 17.3 Hz, 1H, 2-H), 6.46 (dd, ^3J = 1.5 Hz, ^3J = 17.3 Hz, 1H, 3-H_{cis}), 7.28−7.41 (m, 5H, Ph). The spectrum is in full agreement with published data.[37]

4-Fluorobenzyl acetate (65) and *N*-(4-fluorobenzyl)acetamide (66). 4-Fluorobenzyl alcohol (**64**) and MeCN were reacted according to GP 1. Chromatography (hexanes/EtOAc, 3:1) afforded **66** (80 mg, 63%) as a white solid. R_f = 0.13 (hexanes/EtOAc, 1:2). ^{1}H NMR (300 MHz, acetone-D_6) δ 1.93 (s, 3H, CH_3), 4.34 (d, ^3J = 6.0 Hz, 2H, CH_2Ar), 7.05 (t, ^3J = 8.8 Hz, 2H, Ar), 7.33 (dd, ^4J = 5.6 Hz, ^3J = 8.5 Hz, 2H, Ar), 7.64 (s, 1H, NH). The spectrum is in full agreement with published data.[38] **Side product: 4-Fluorobenzyl Acetate (65):** Chromatography (hexanes/EtOAc, 2:1) afforded **65** (37 mg, 29%) as a colourless oil. R_f = 0.44 (hexanes/EtOAc, 3:1). ^{1}H NMR (300 MHz, $CDCl_3$) δ 2.09 (s, 3H, Me), 5.07 (s, 2H, CH_2), 7.05 (t, ^3J = 8.6 Hz, 2H, Ar), 7.34 (dd, ^3J = 8.3 Hz, ^4J = 5.6 Hz, 2H, Ar). The spectrum is in full agreement with published data.[39]

[34] Ginisty, M.; Roy, M.-N.; Charette, A. B. *J. Org. Chem.*, **2008**, *73*, 2542–2547.
[35] Matsumoto, K.; Hashimoto, S.; Uchida, T.; Okamoto, T.; Otani, S. *Chem. Ber.*, **1989**, *122*, 1357–1363.
[36] Miyabe, H.; Ueda, M.; Nishimura, A.; Naito, T. *Tetrahedron* **2004**, *60*, 4227–4235.
[37] Grasa, G. A.; Güveli, T.; Singh, R.; Nolan, S. P. *J. Org. Chem.*, **2003**, *68*, 2812–2819.
[38] Shine, S. J.; Yueh, W. *J. Org. Chem.*, **1994**, *59*, 3553–3559.
[39] Kadam, S. T.; Kim, S. S. *Synthesis*, **2008**, 267–271.

***N*-(4-Fluorobenzyl)phenylacetamide (67).** 4-Fluorobenzyl alcohol (**64**) and BnCN were reacted according to GP 3. Chromatography (hexanes/EtOAc, 3:1) afforded **67** (146 mg, 79%) as a white solid. R_f = 0.18 (hexanes/EtOAc, 2:1). IR (ATR): $\tilde{\nu}$ (cm^{-1}) = 3280 (m), 3061 (w), 1641 (s, C=O), 1603 (m), 1548 (m), 1504 (m), 1491 (m), 1452 (m), 1432 (m), 1224 (m), 1028 (m), 823 (m), 752 (m), 716 (m), 694 (s), 569 (m). ^{1}H NMR (400 MHz, $CDCl_3$) δ 3.63 (s, 2H, CH_2Ph), 4.37 (d, ^3J = 5.8 Hz, 2H, CH_2Ar), 5.67 (s, 1H, NH), 6.97 (t, ^3J = 8.6 Hz, 2H, Ar), 7.15 (dd, ^4J = 5.6 Hz, ^3J = 8.1 Hz, 2H, Ar), 7.24–7.39 (m, 5H, Ph). ^{13}C NMR (75 MHz, $CDCl_3$) δ 43.0 (t), 43.9 (t), 115.6 (2 d), 127.6 (d), 129.2 (2 d), 129.3 (2 d), 129.6 (2 d), 134.1 (s), 134.8 (s), 162.2 (s), 171.0 (s). MS (EI, 80 °C): *m*/*z* (%) = 244 (12), 243 (97) [M^+], 109 (100), 92 (15), 91 (49). HRMS ($^{12}C_{15}{}^{1}H_{14}{}^{19}F^{14}N^{16}O$, EI): calcd. 243.1059 amu; found 243.1057 amu. Anal. calcd for $C_{15}H_{14}FNO$ (243.11): C 74.06, H 5.80, N 5.76; found C 74.29, H 5.78, N 5.56.

***N*-(4-Fluorobenzyl)benzamide (68).** 4-Fluorobenzyl alcohol (**64**) and PhCN were reacted according to GP 3. Chromatography (hexanes/EtOAc, 1:1) afforded **68** (122 mg, 70%) as a white solid. R_f = 0.22 (hexanes/EtOAc, 2:1). ^{1}H NMR (300 MHz, $CDCl_3$) δ 4.63 (d, ^3J = 5.7 Hz, 2H, CH_2), 6.40 (s, 1H, NH), 7.04 (t, ^3J = 8.5 Hz, 2H, Ar), 7.34 (dd, ^4J = 5.6 Hz, ^3J = 8.1 Hz, 2H, Ar), 7.39−7.55 (m, 3H, Ph), 7.79 (d, ^3J = 7.9 Hz, 2H, Ph). The spectrum is in full agreement with published data.[40]

***N*-(4-Fluorobenzyl)acrylamide (69).** 4-Fluorobenzyl alcohol (**64**) and acrylonitrile were reacted according to GP 1. Chromatography (hexanes/EtOAc, 1:2) afforded **69** (121 mg, 89%) as a white solid. R_f = 0.29 (hexanes/EtOAc, 1:1). IR (ATR): $\tilde{\nu}$ (cm^{-1}) = 3270 (w), 1654 (m, C=O), 1620 (m), 1543 (m), 1509 (m), 1213 (m), 980 (m), 949 (m), 834 (m), 698 (m), 553 (m), 486 (m). ^{1}H NMR (400 MHz, $CDCl_3$) δ 4.49 (d, ^3J = 5.8 Hz, 2H, ArCH_2), 5.69 (dd, ^3J = 10.3 Hz, ^2J = 1.3 Hz, 1H, 3-H_{trans}), 6.10 (s, 1H, NH), 6.14 (dd, ^3J = 17.0 Hz, ^3J = 10.3 Hz, 1H, 2-H), 6.33 (dd, ^3J = 17,0 Hz, ^2J = 1.3 Hz, 1H, 3-H_{cis}), 7.03 (t, ^3J = 8.6 Hz, 2H, Ar), 7.25–7.32 (m, 2H, Ar). ^{13}C NMR (400 MHz, $CDCl_3$) δ 43.1 (t), 115.7 (2 d), 127.1 (t), 129.7 (2 d), 130.7 (d), 134.0 (s), 162.3 (s), 165.6 (s). MS (EI, 60 °C): *m*/*z* (%) = 180 (14), 179 (100) [M^+], 178 (18), 136 (9), 135 (38), 124 (37), 122 (10), 109 (45), 55 (31). HRMS ($^{12}C_{10}{}^{1}H_{10}{}^{19}F^{14}N^{16}O$, EI): calcd. 179.0746 amu; found 179.0744 amu. $C_{10}H_{10}FNO$ (179.07): calcd. C 67.03, H 5.62, N 7.82; found C 66.84, H 5.58, N 7.69.

---

40 Khalafi-Nezhad, A.; Foroughi, H. O.; Doroodmand, M. M.; Panahi, F. *J. Mater. Chem.*, **2011**, *21*, 12842–12851.

B. ^{1}H and ^{13}C NMR spectra

1. (9*H*-Fluoren-9-yl)methyl acetate (2).

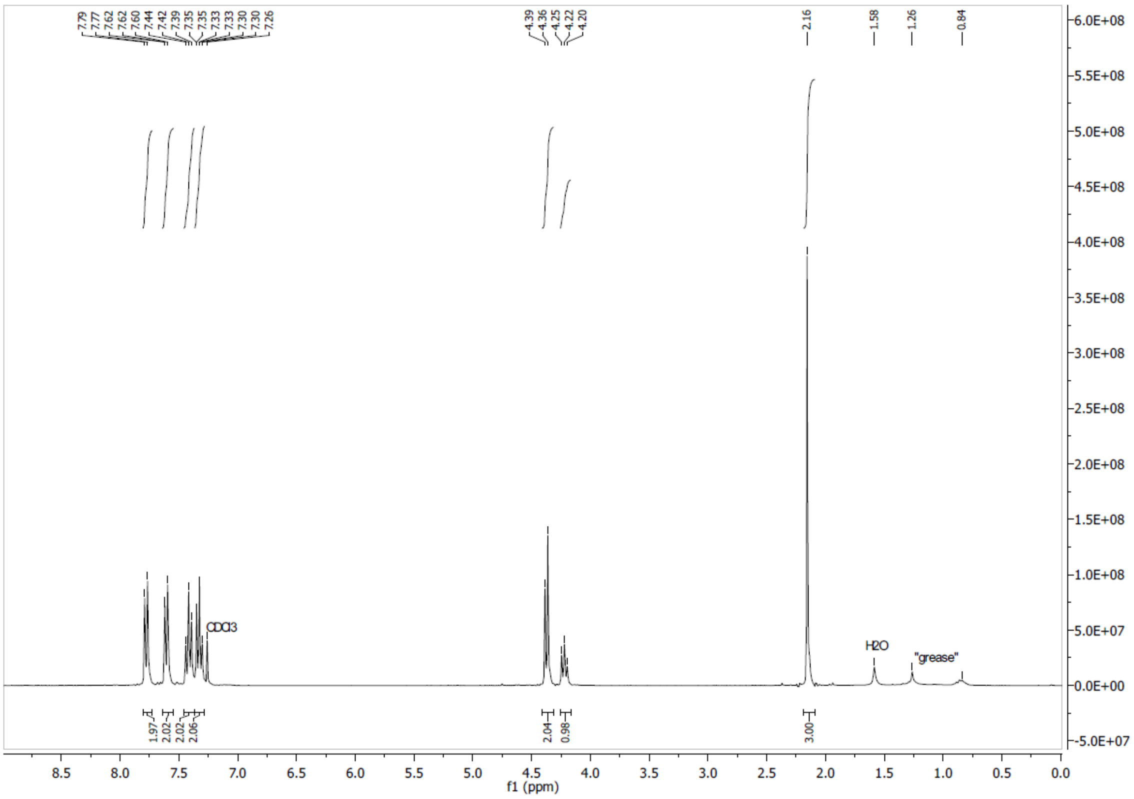

2. (9*H*-Fluoren-9-yl)methyl phenylacetate (3).

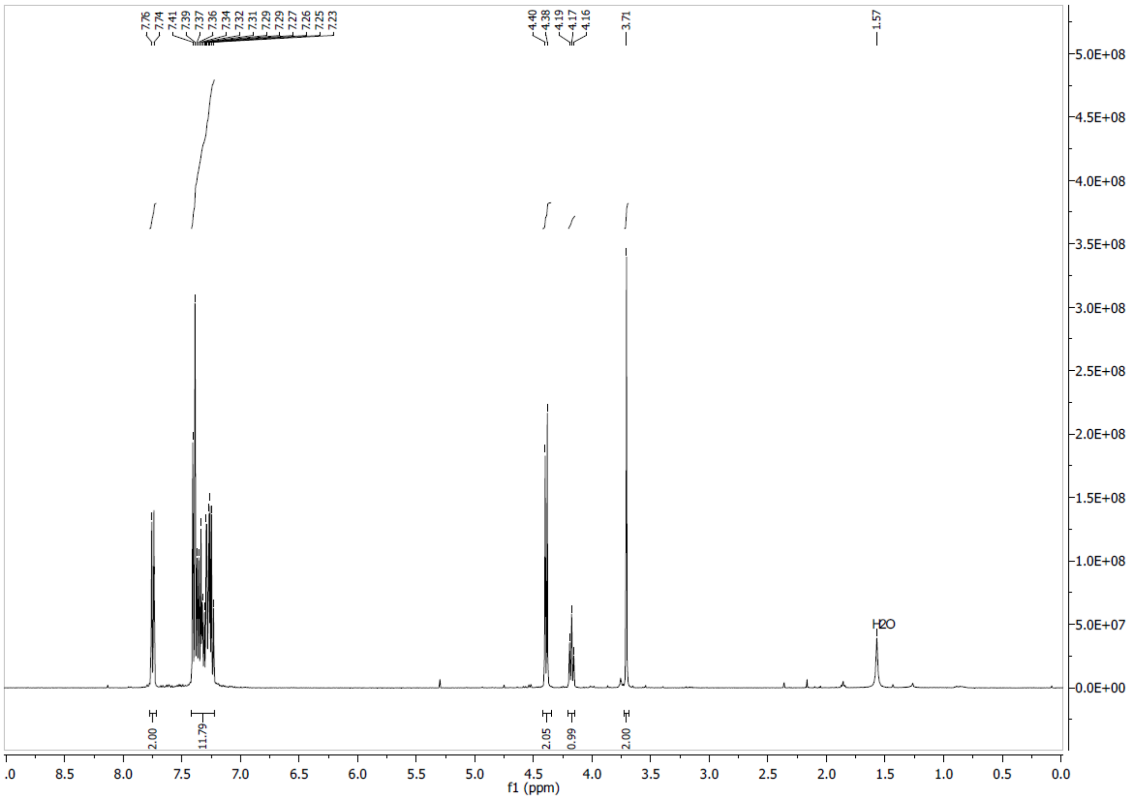

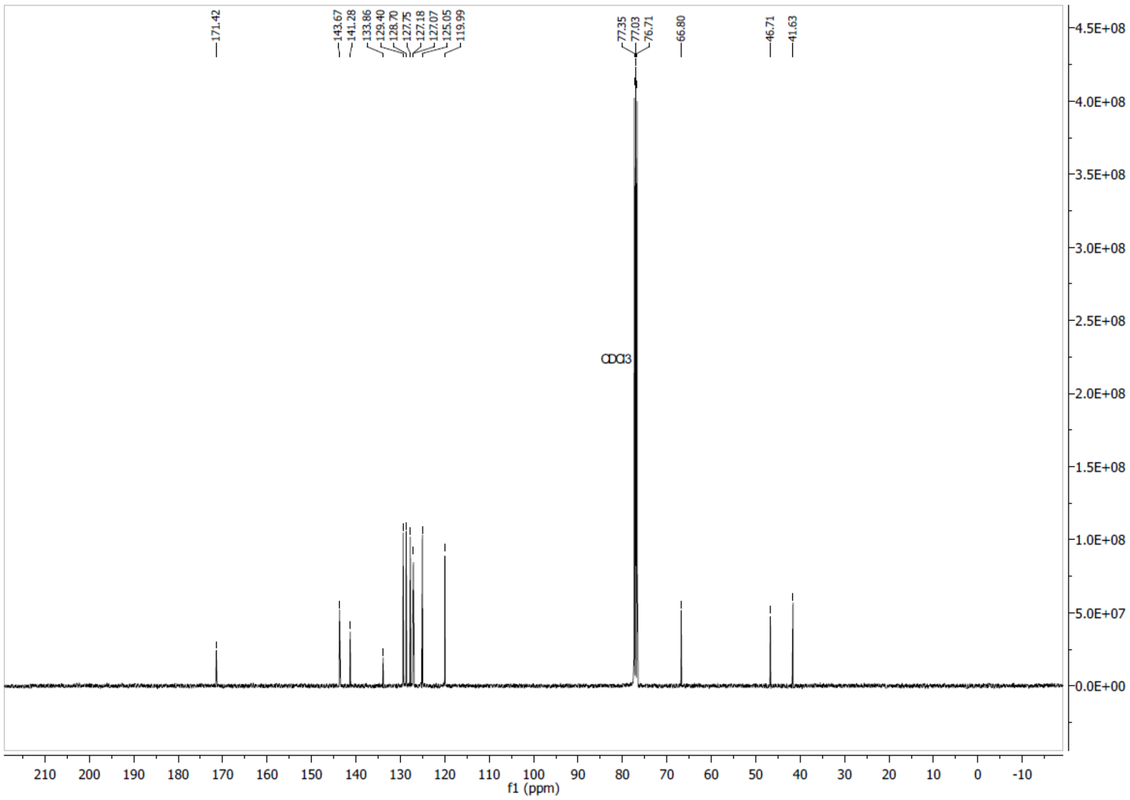

3. **(9*H*-Fluoren-9-yl)methyl benzoate (4).**

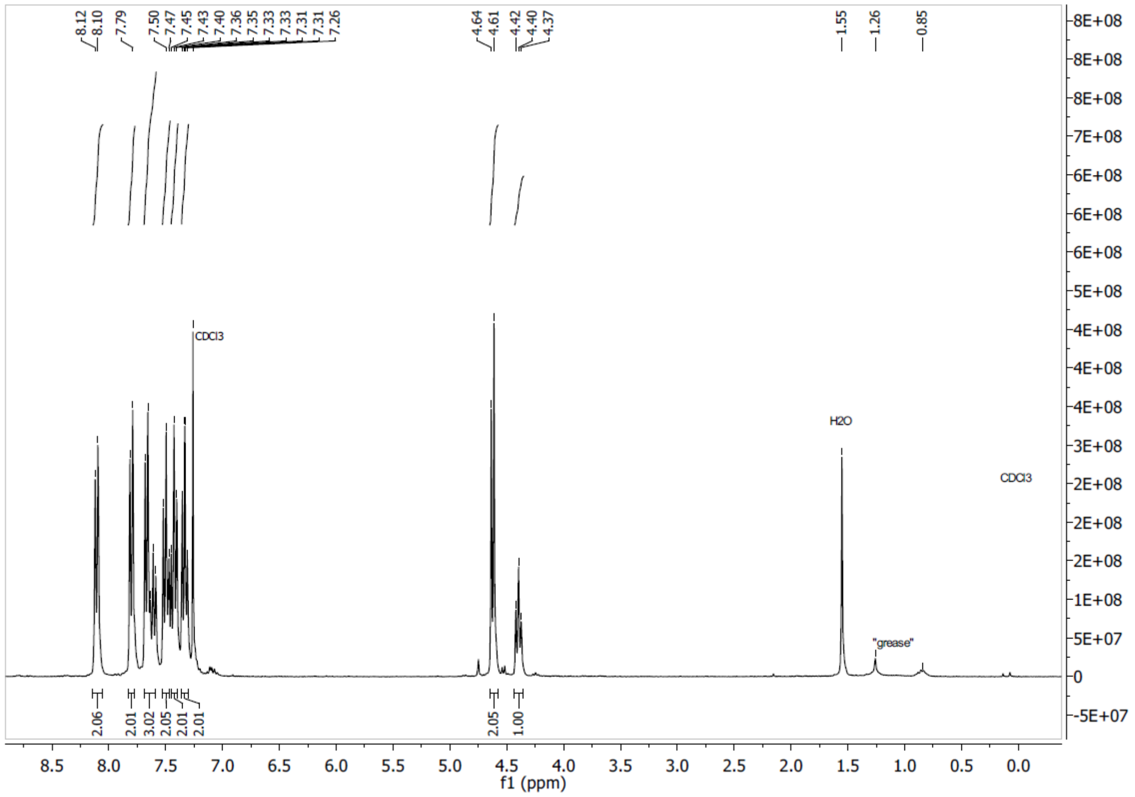

4. **(9*H*-Fluoren-9-yl)methyl acrylate (5).**

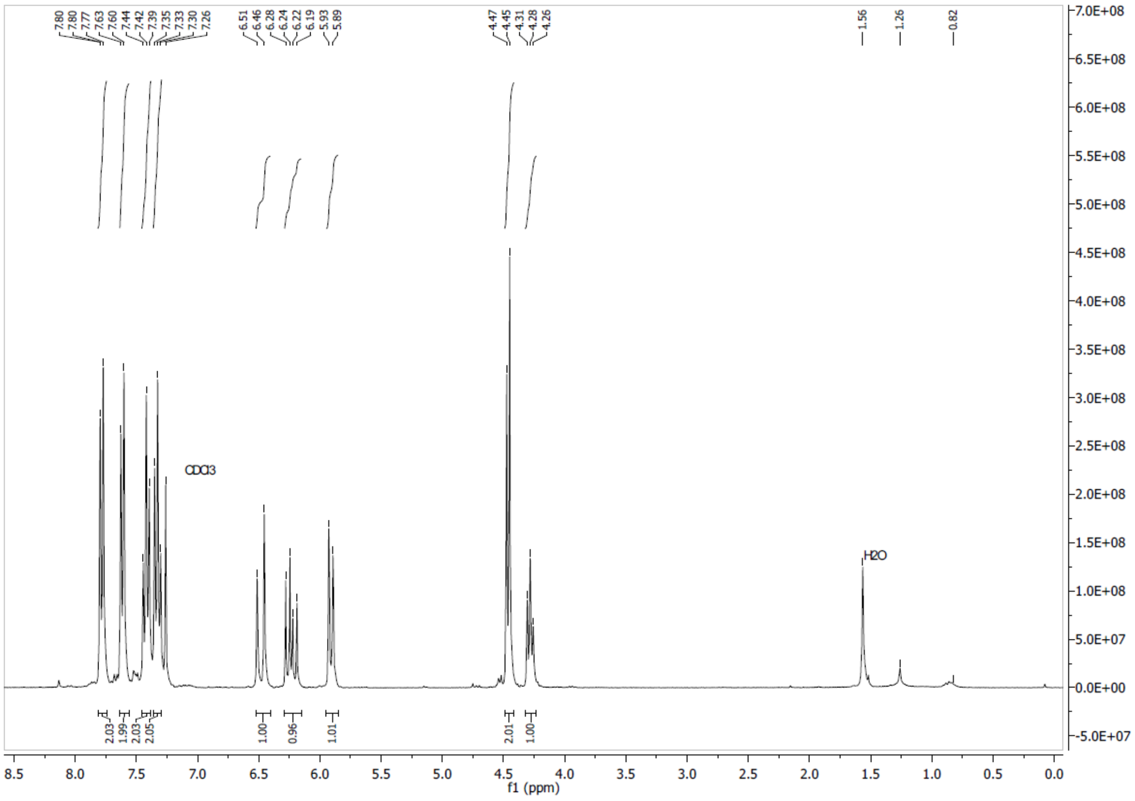

5. 1-Decyl acetate (7).

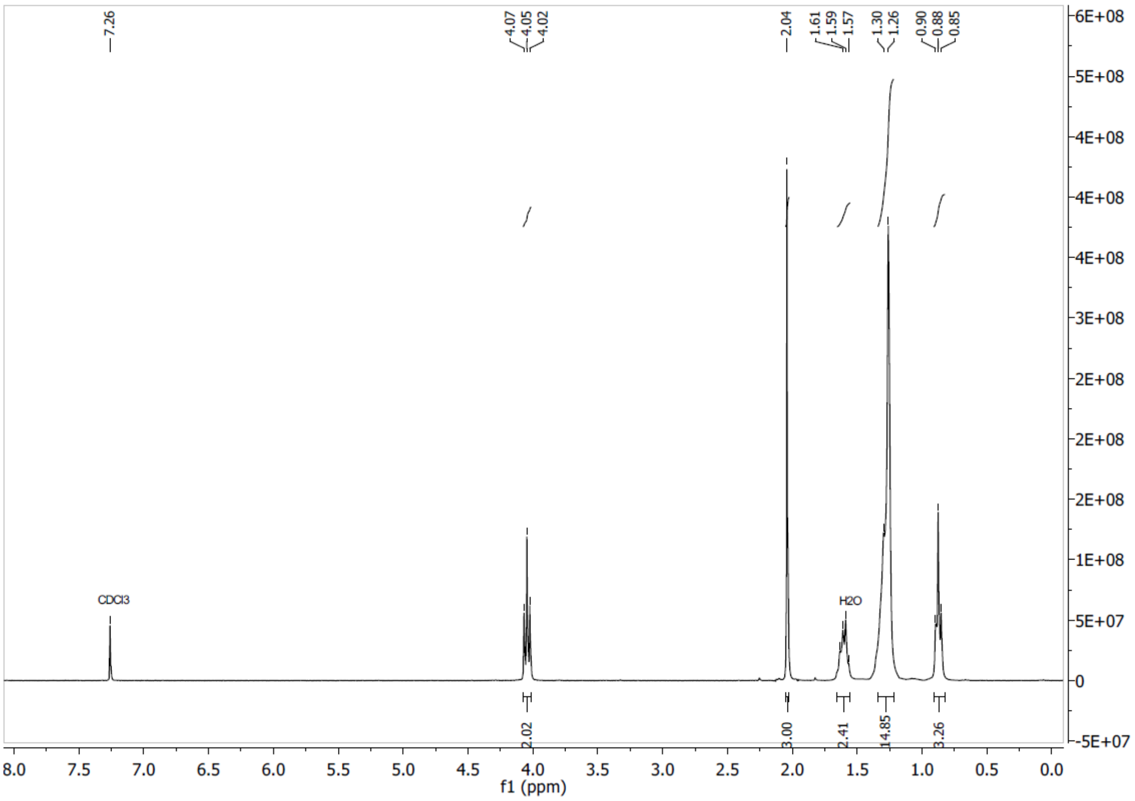

6. 1-Decyl phenylacetate (8).

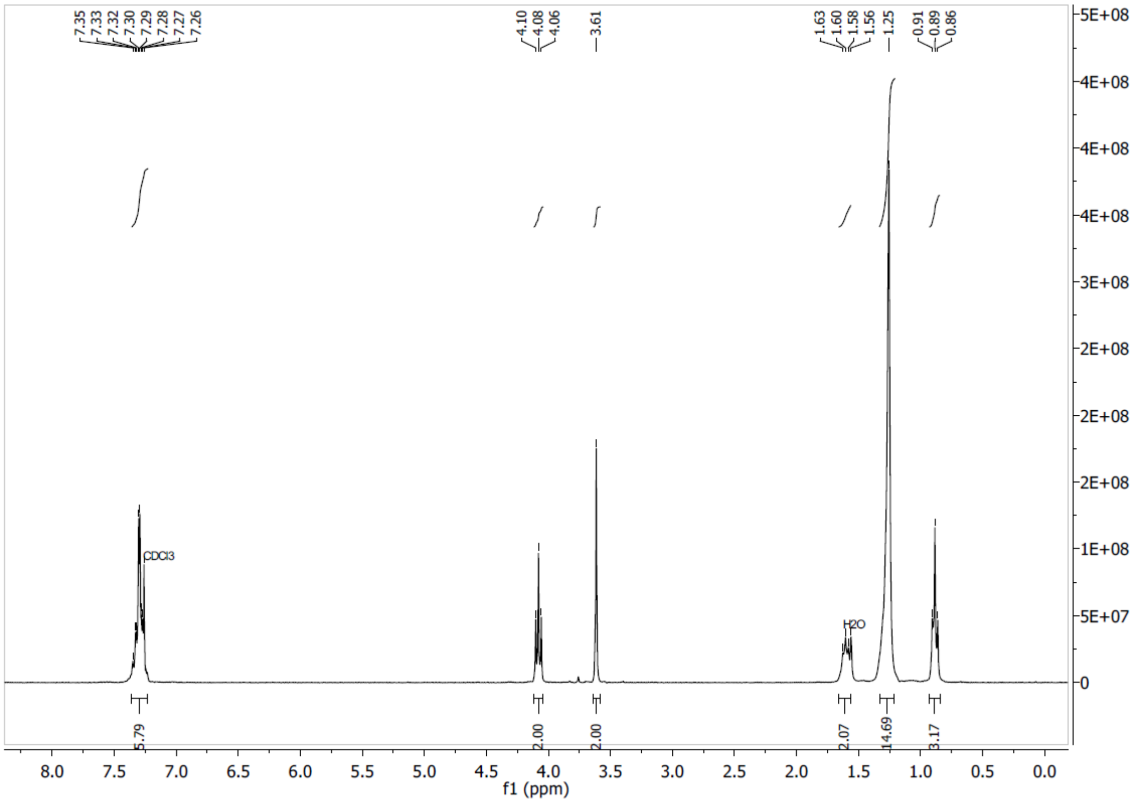

7. **1-Decyl benzoate (9).**

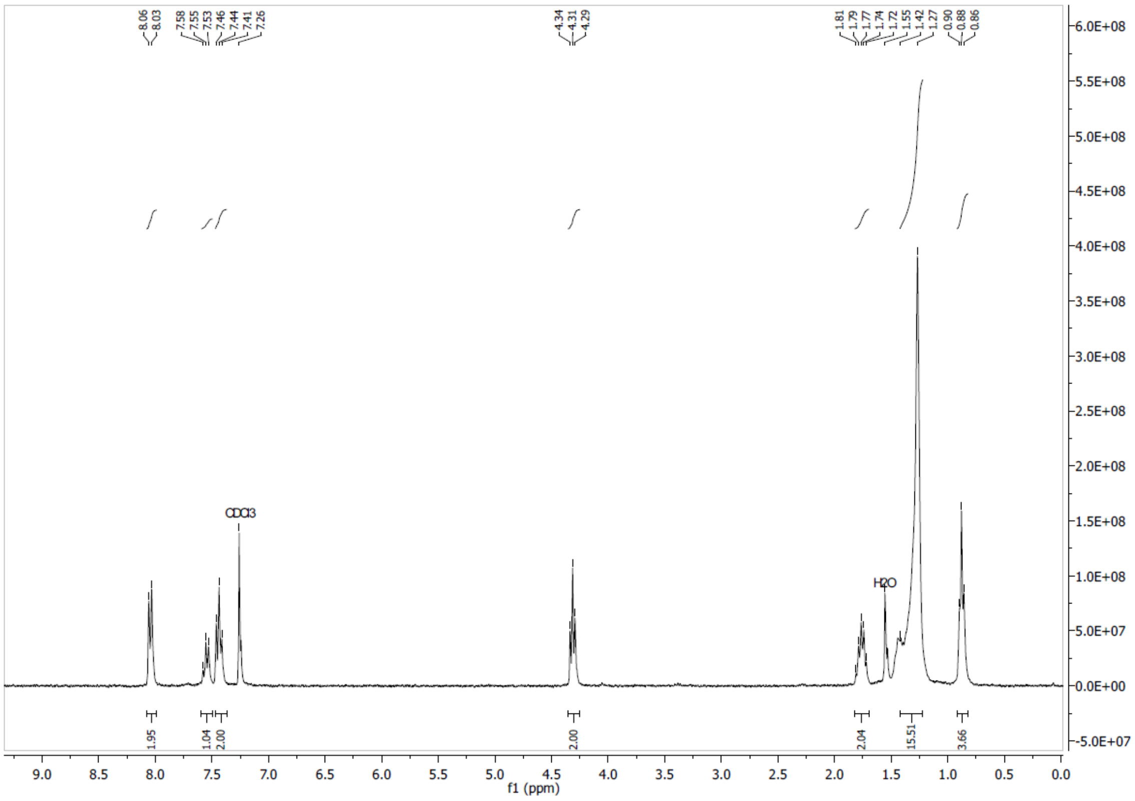

8. **1-Decyl acrylate (10).**

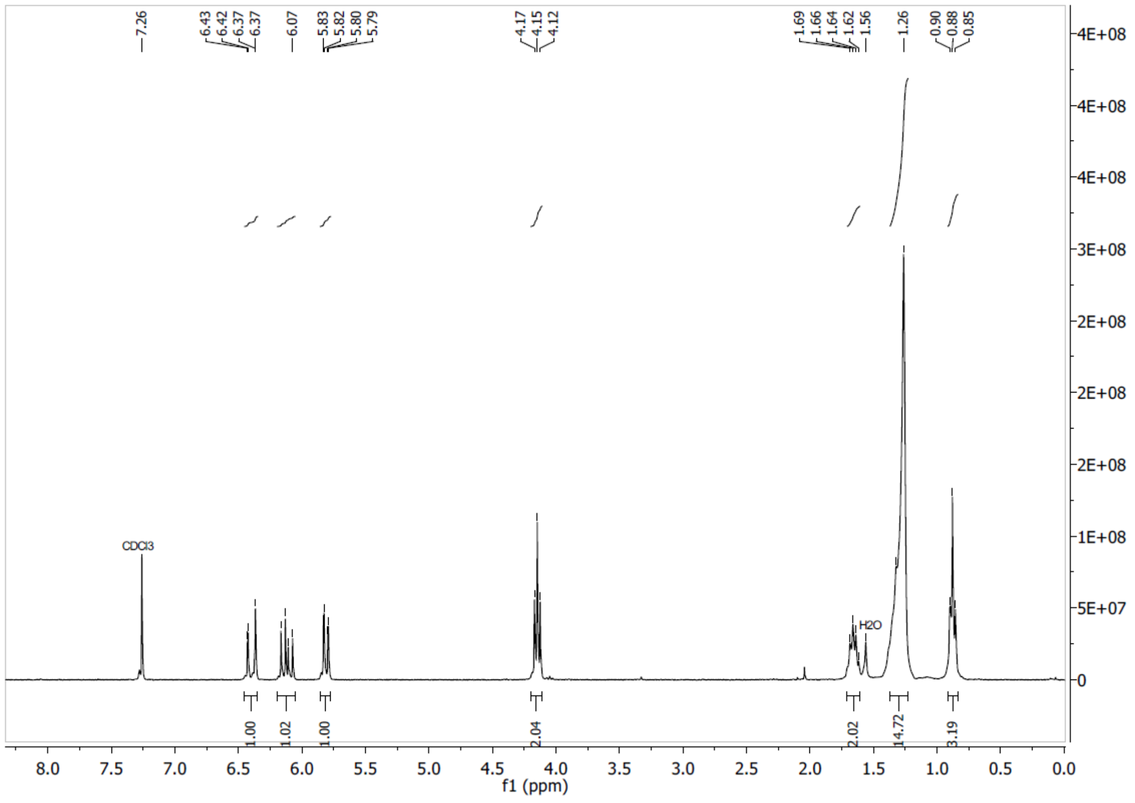

9. 6-Chlorohexyl Acetate (12).

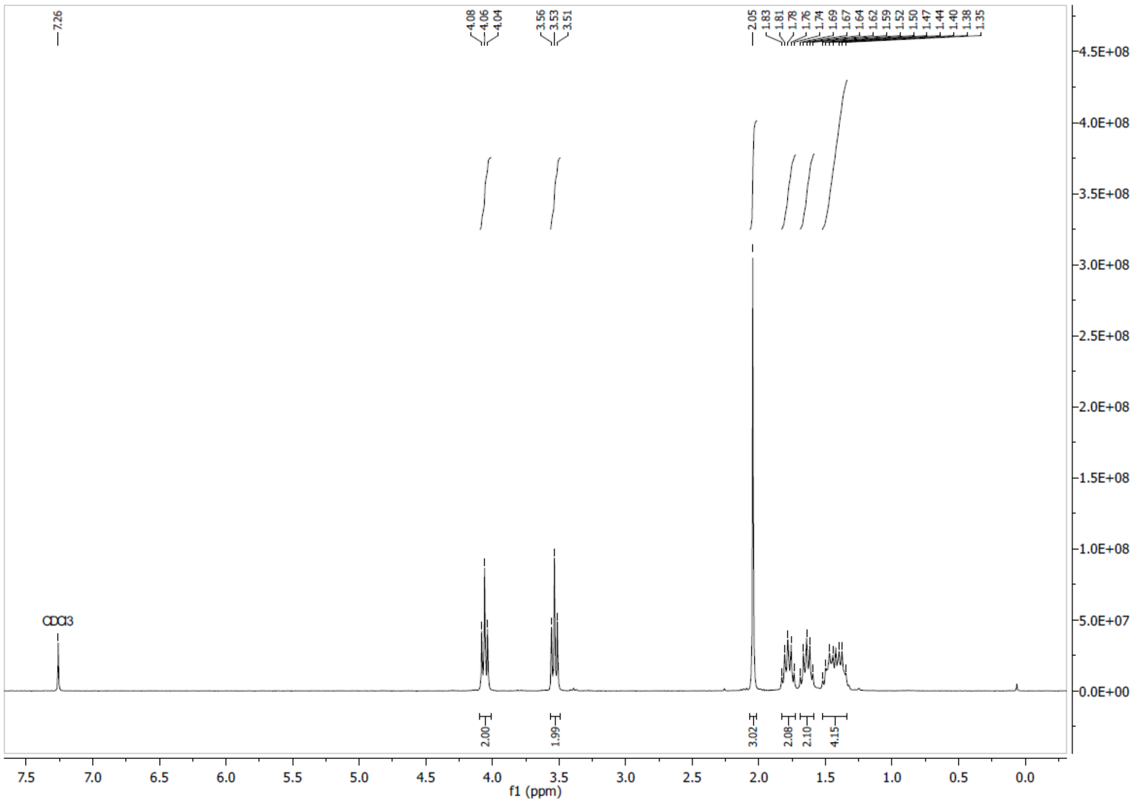

10. 6-Chlorohexyl phenylacetate (13).

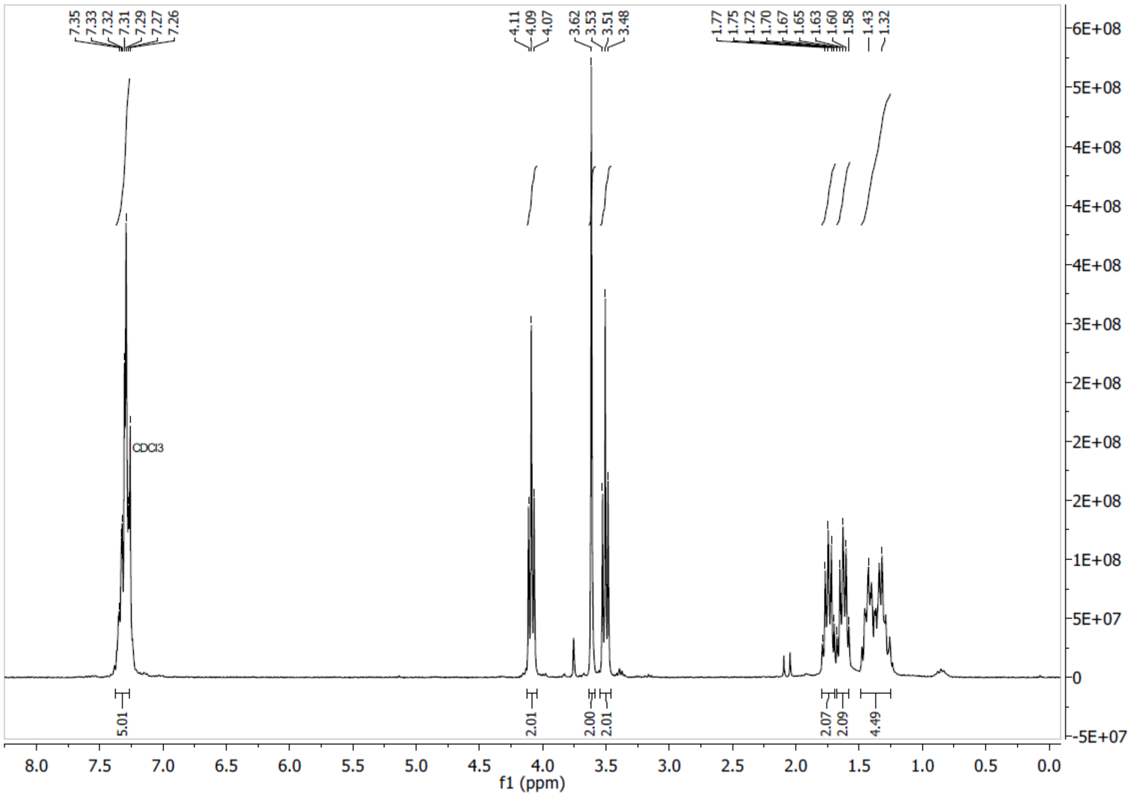

11. 6-Chlorohexyl benzoate (14).

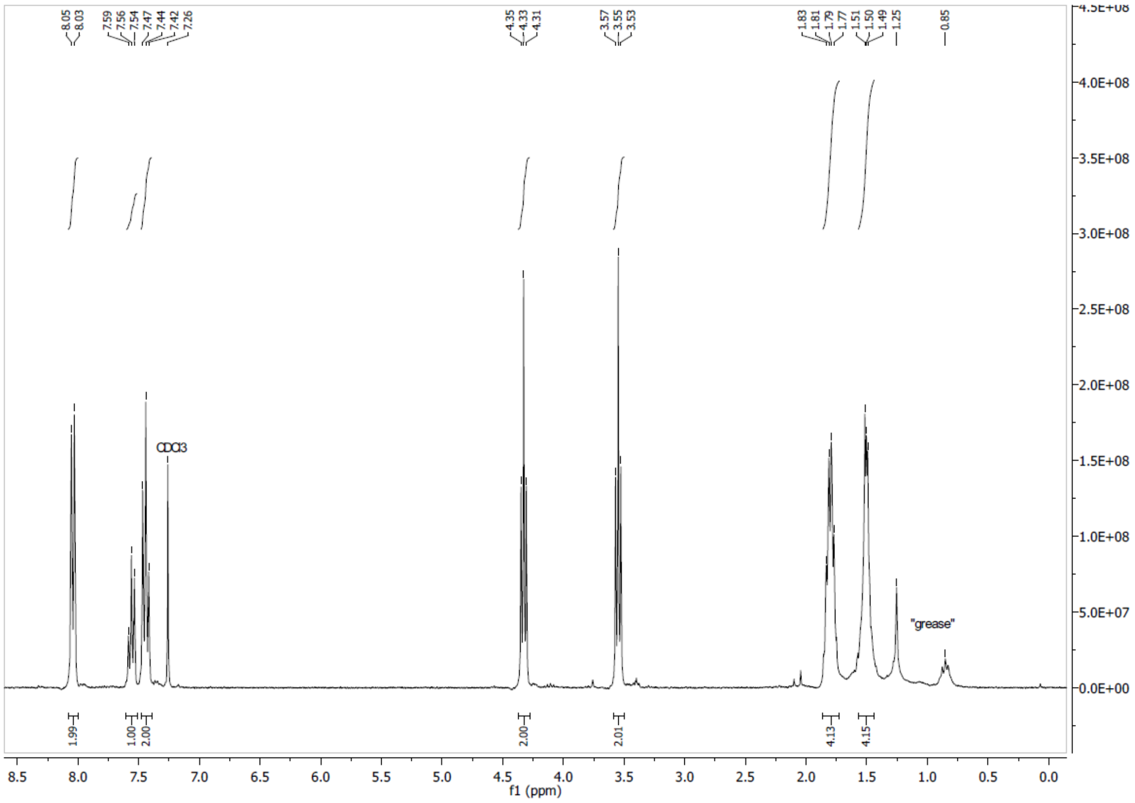

12. 6-Chlorohexyl acrylate (15).

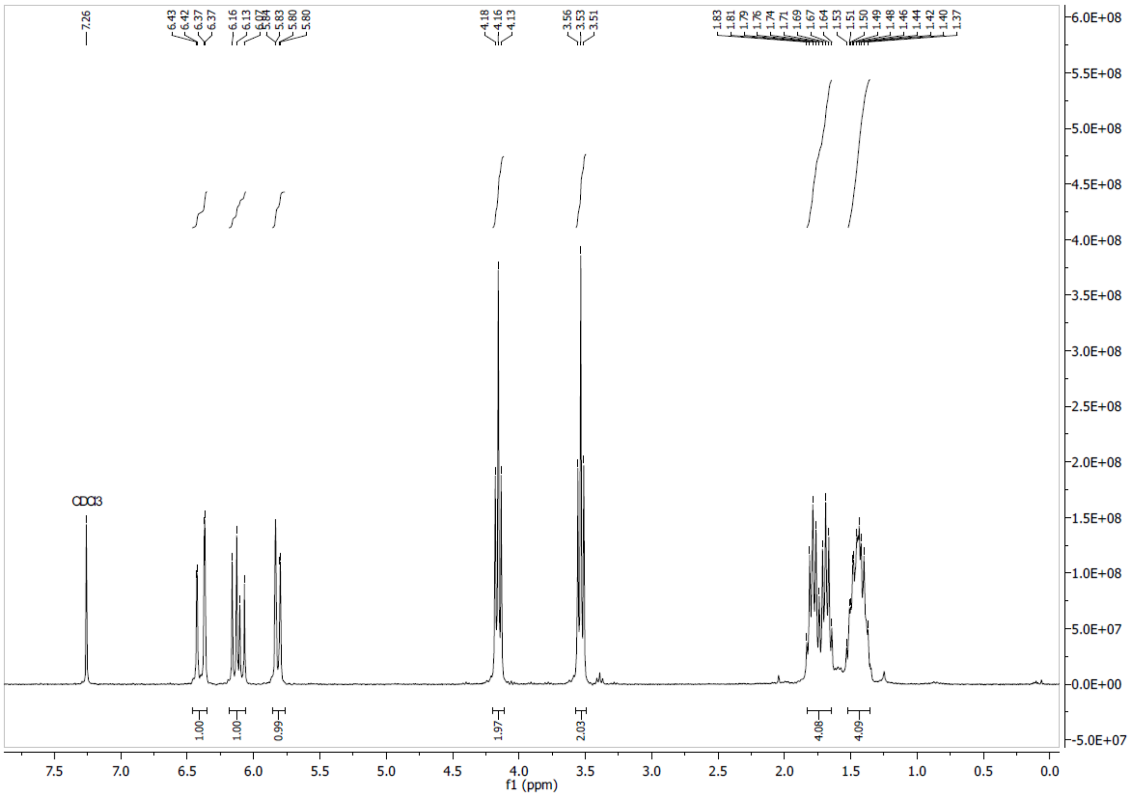

13. 2-(2-Ethoxyethoxy)ethyl acetate (17).

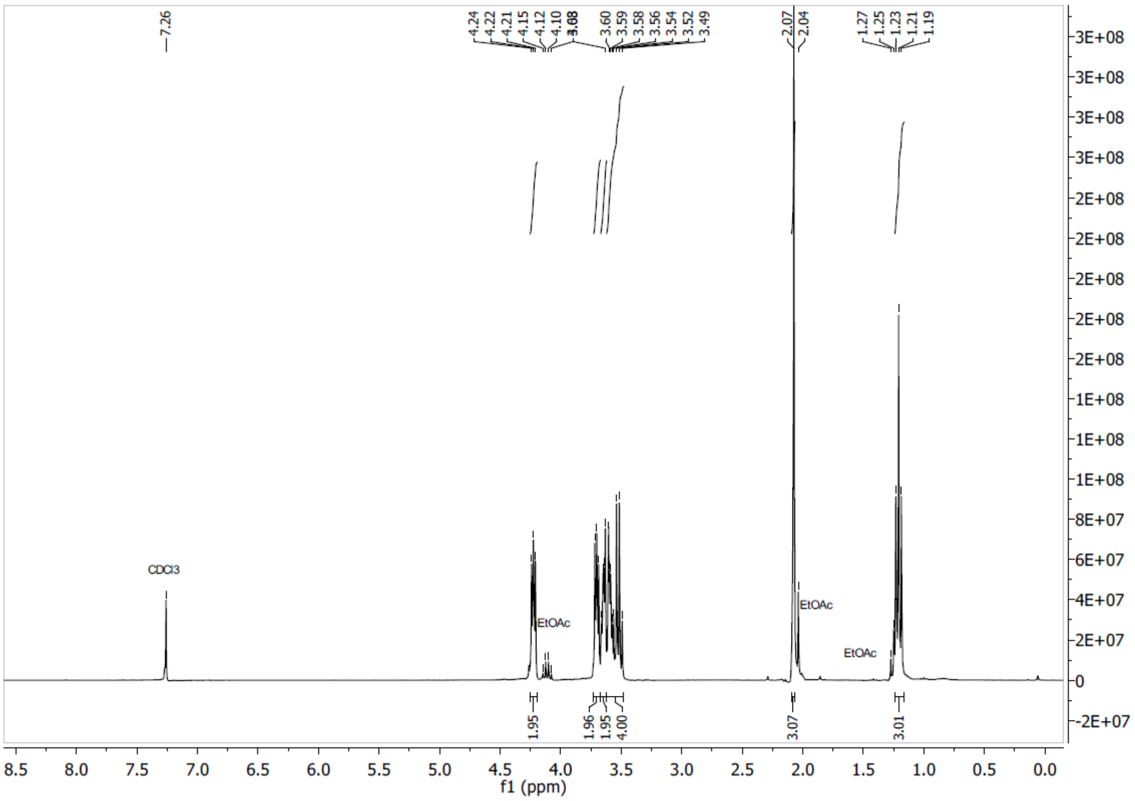

14. 2-(2-Ethoxyethoxy)ethyl phenylacetate (18).

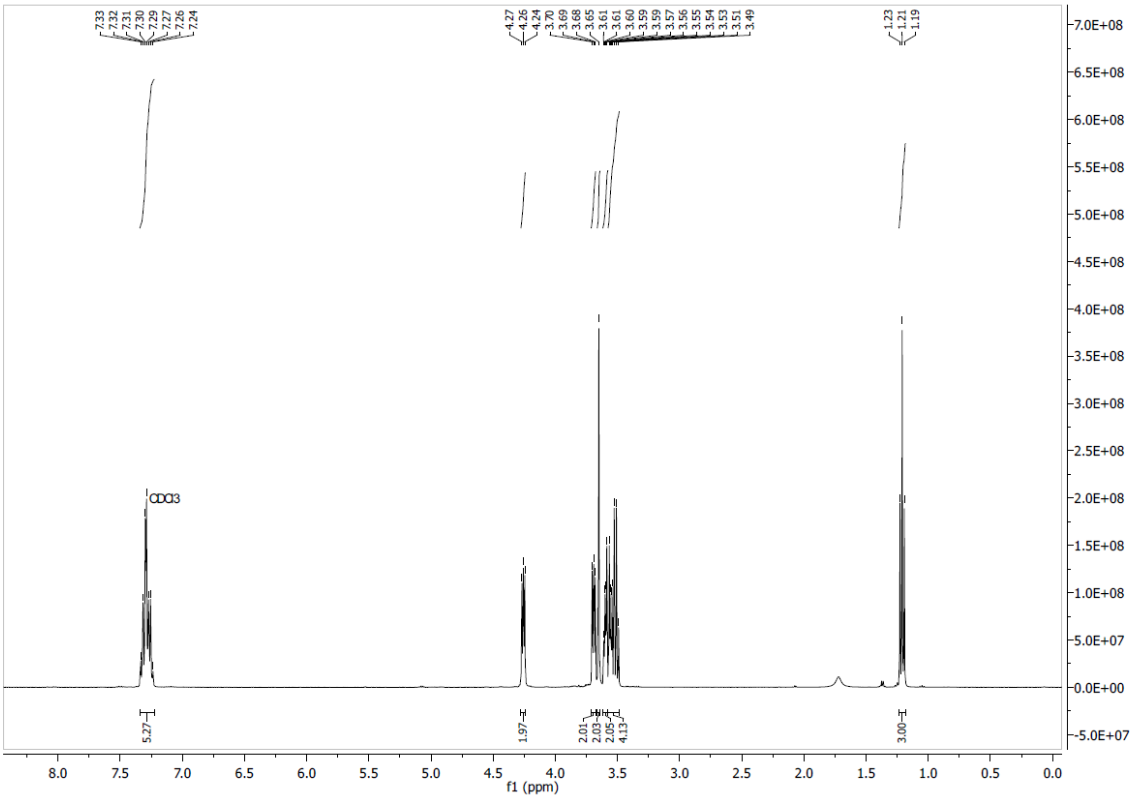

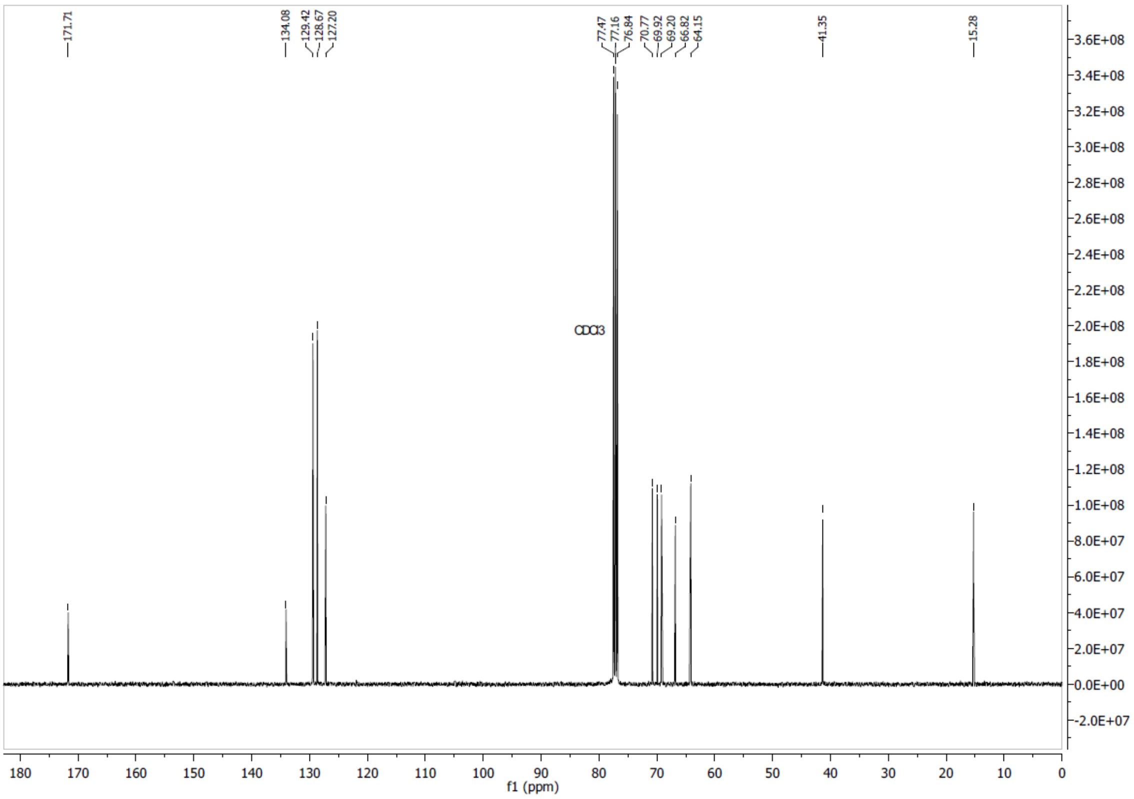

15. 2-(2-Ethoxyethoxy)ethyl benzoate (19).

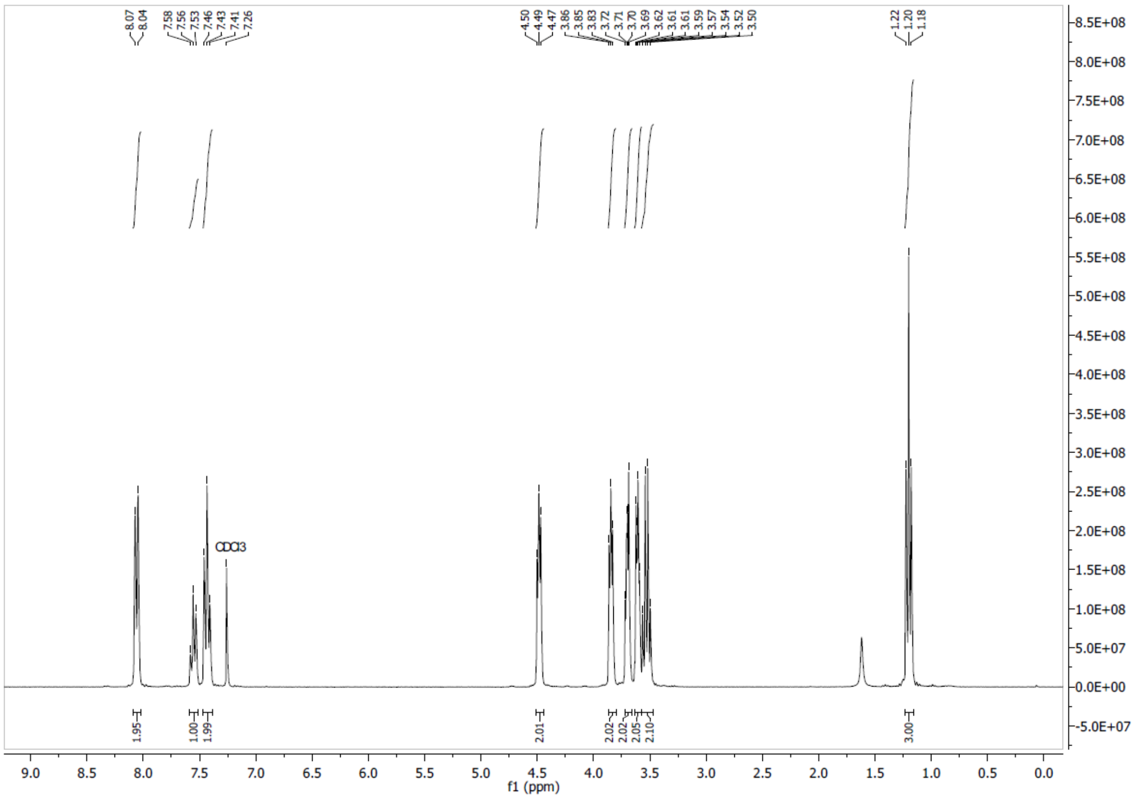

16. 2-(2-Ethoxyethoxy)ethyl acrylate (20).

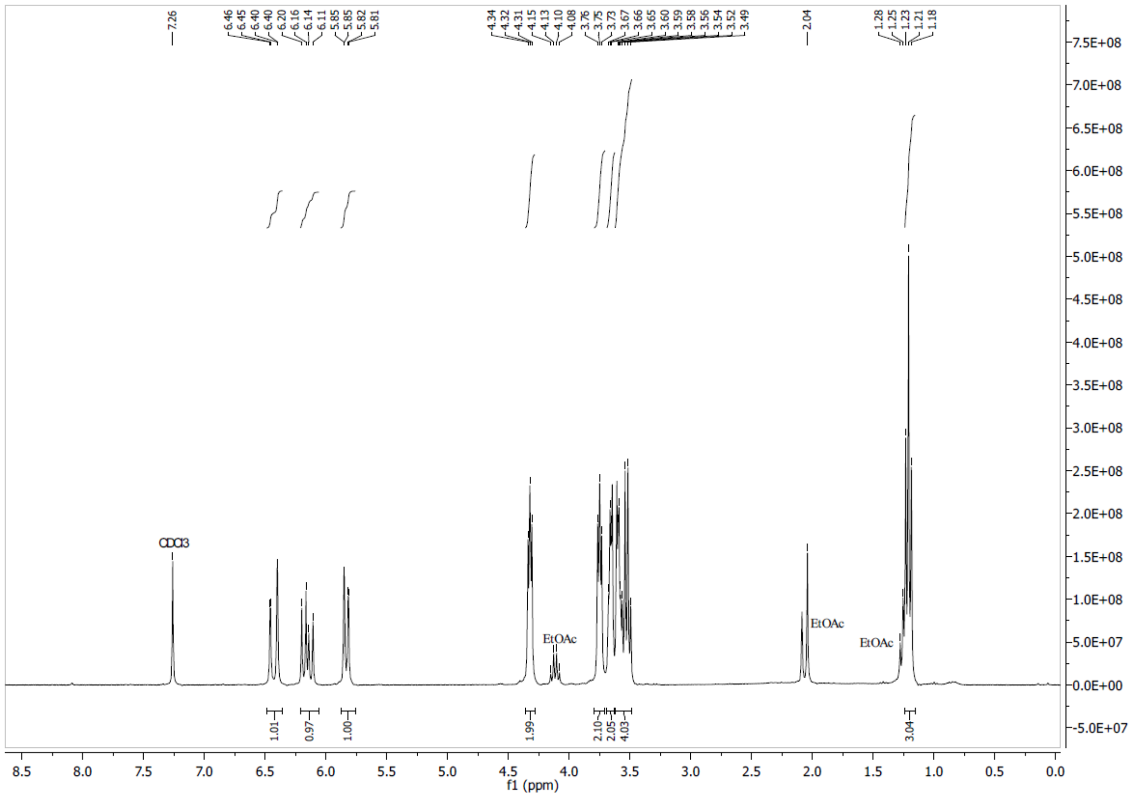

17. 4-Nitrobenzyl acetate (**22**).

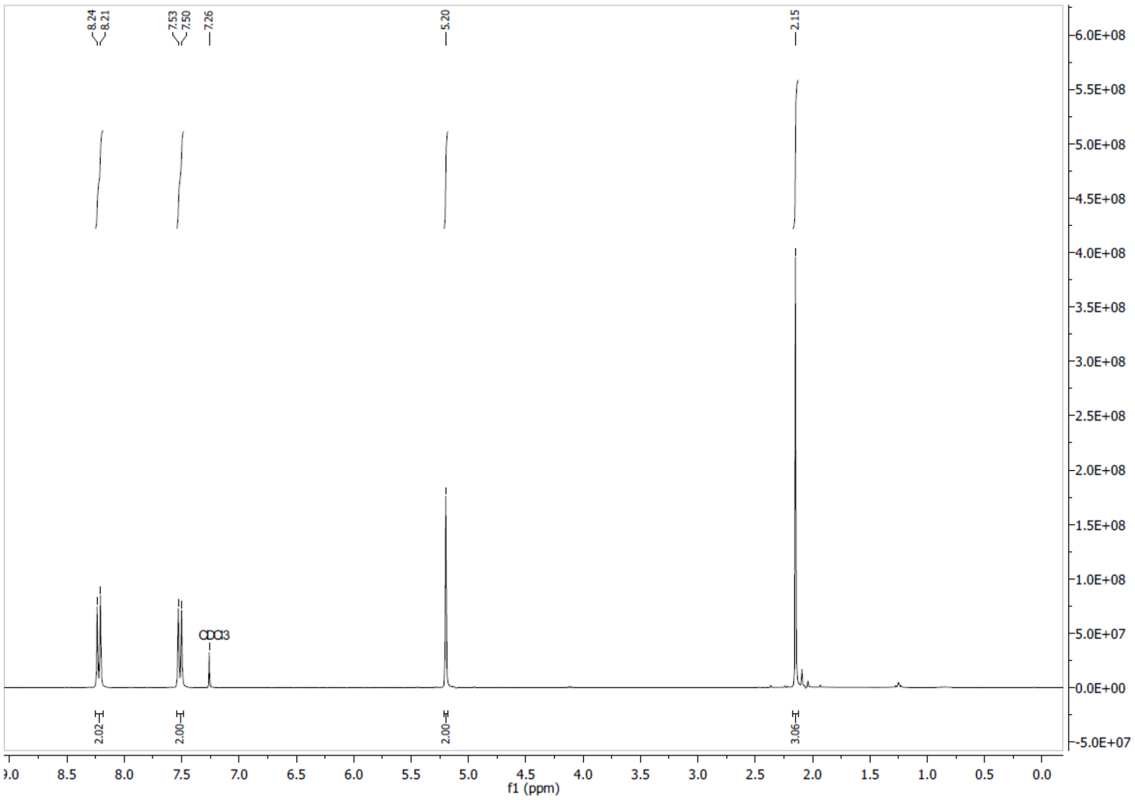

18. 4-Nitrobenzyl phenylacetate (23).

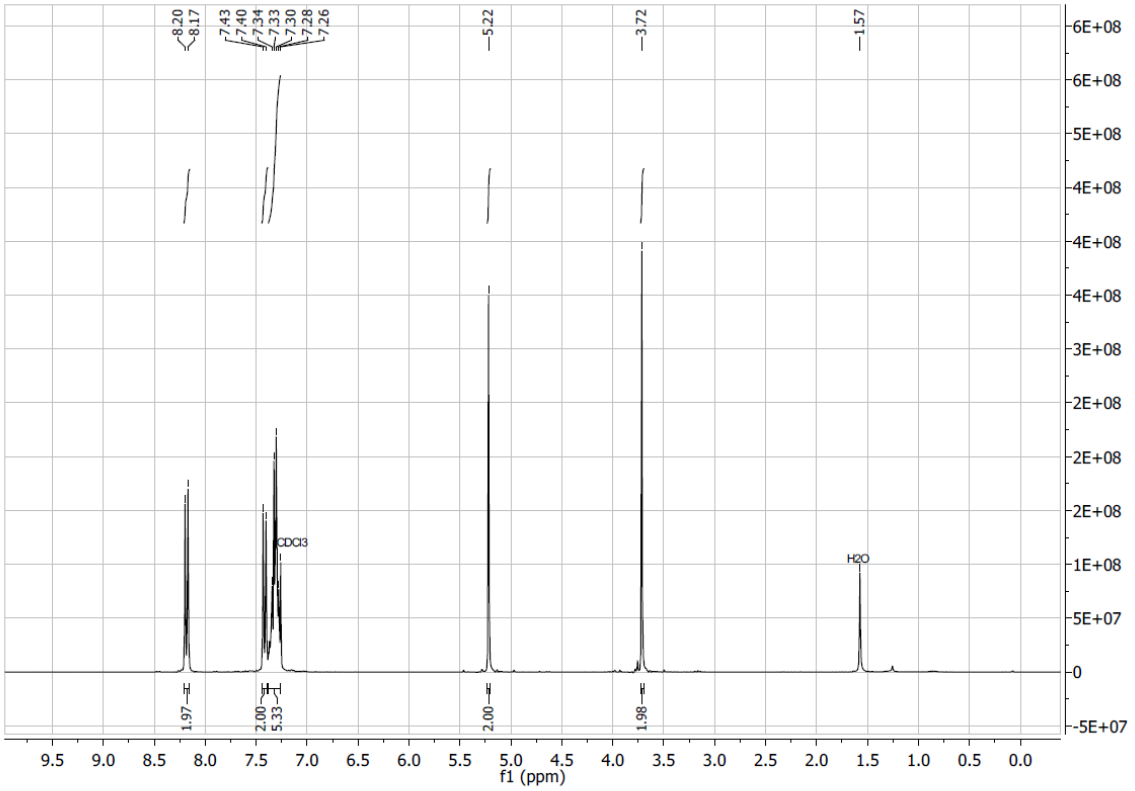

19. 4-Nitrobenzyl benzoate (24).

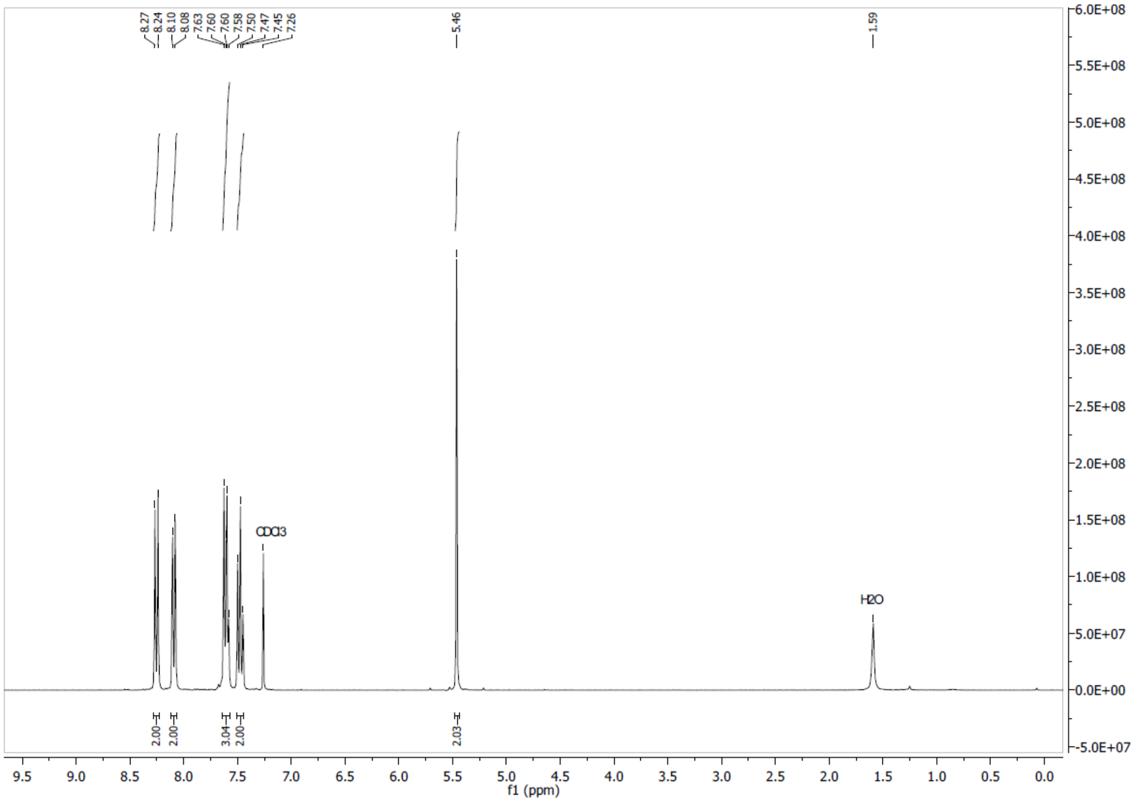

20. 4-Nitrobenzyl acrylate (25).

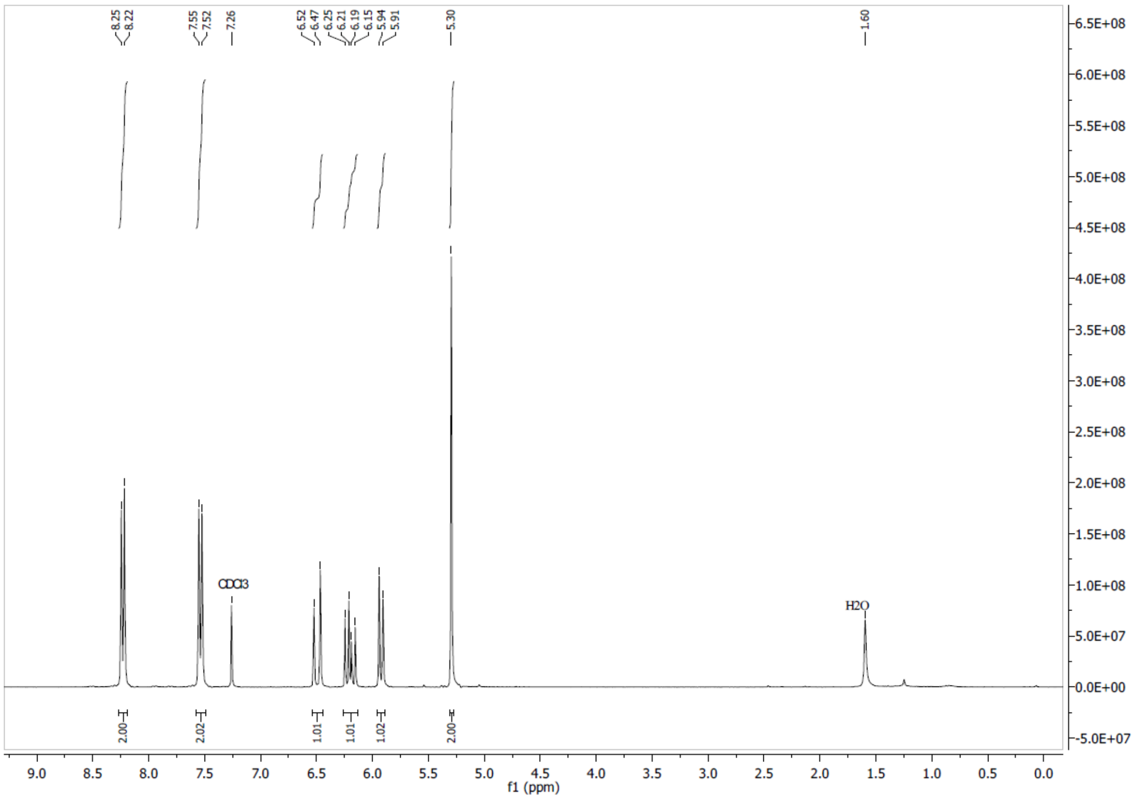

21. 4-(Acetoxymethyl)benzoic acid (27).

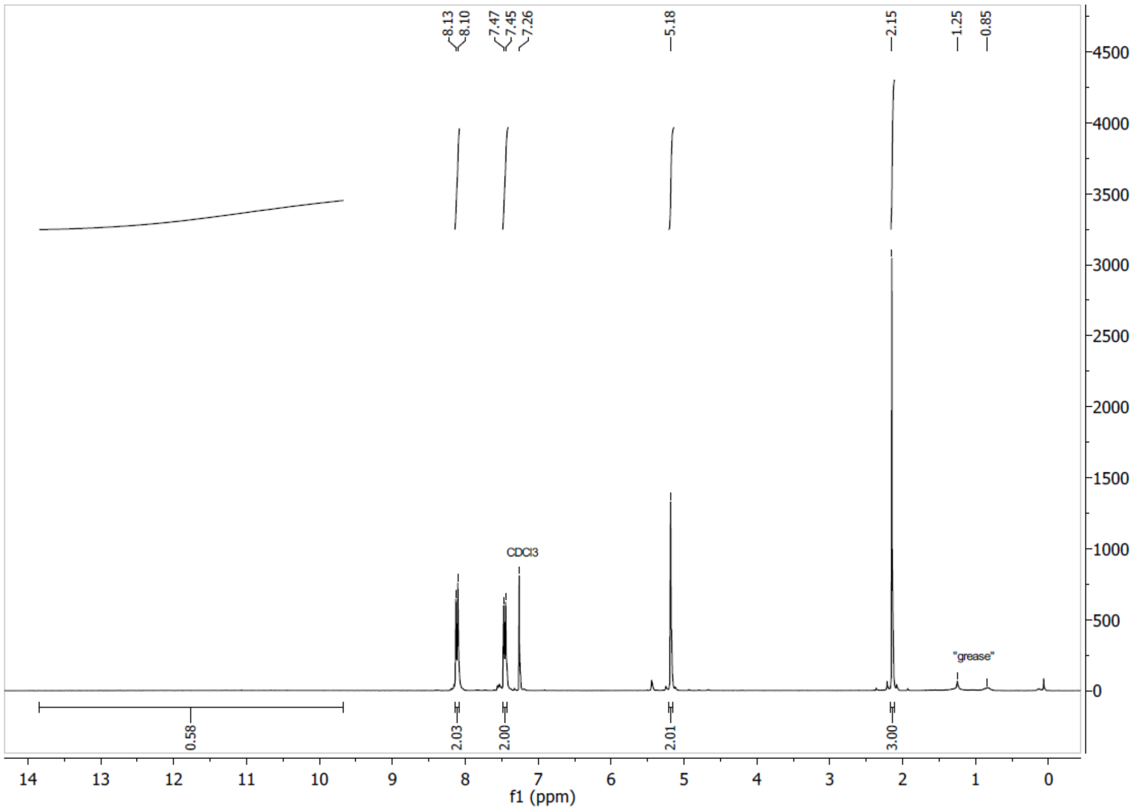

22. 4-[(Phenylacetoxy)methyl]benzoic acid (28).

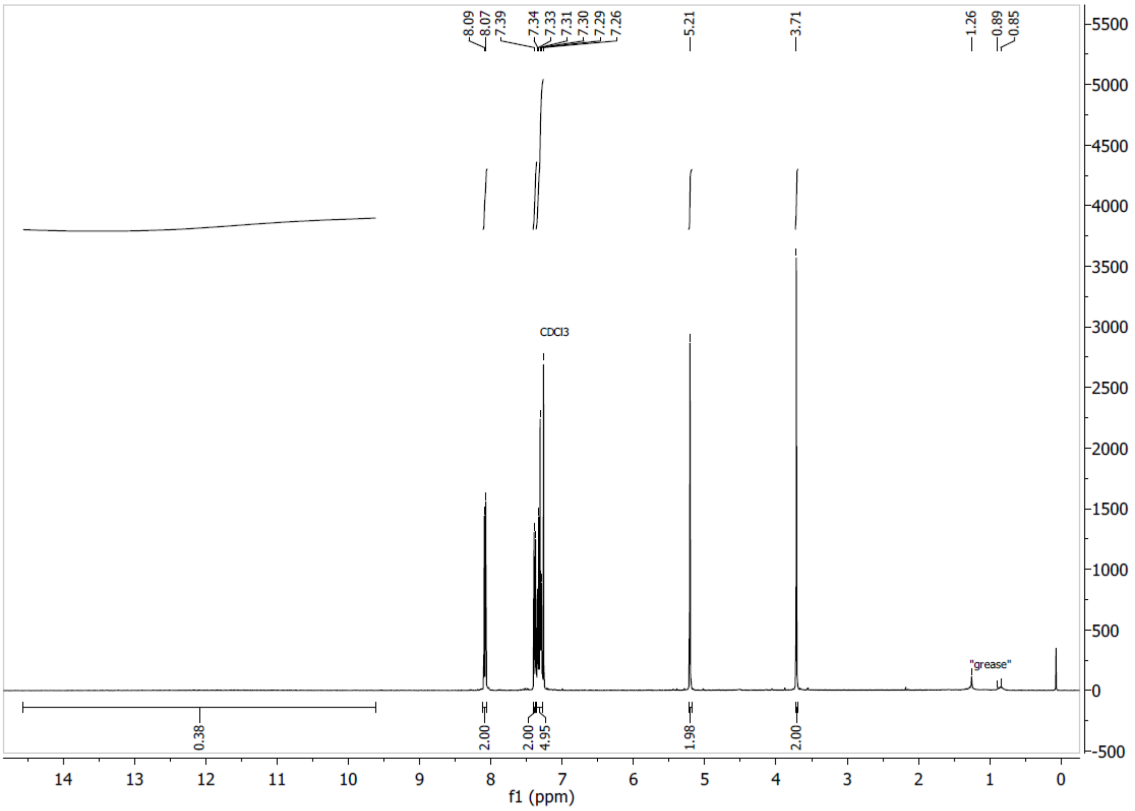

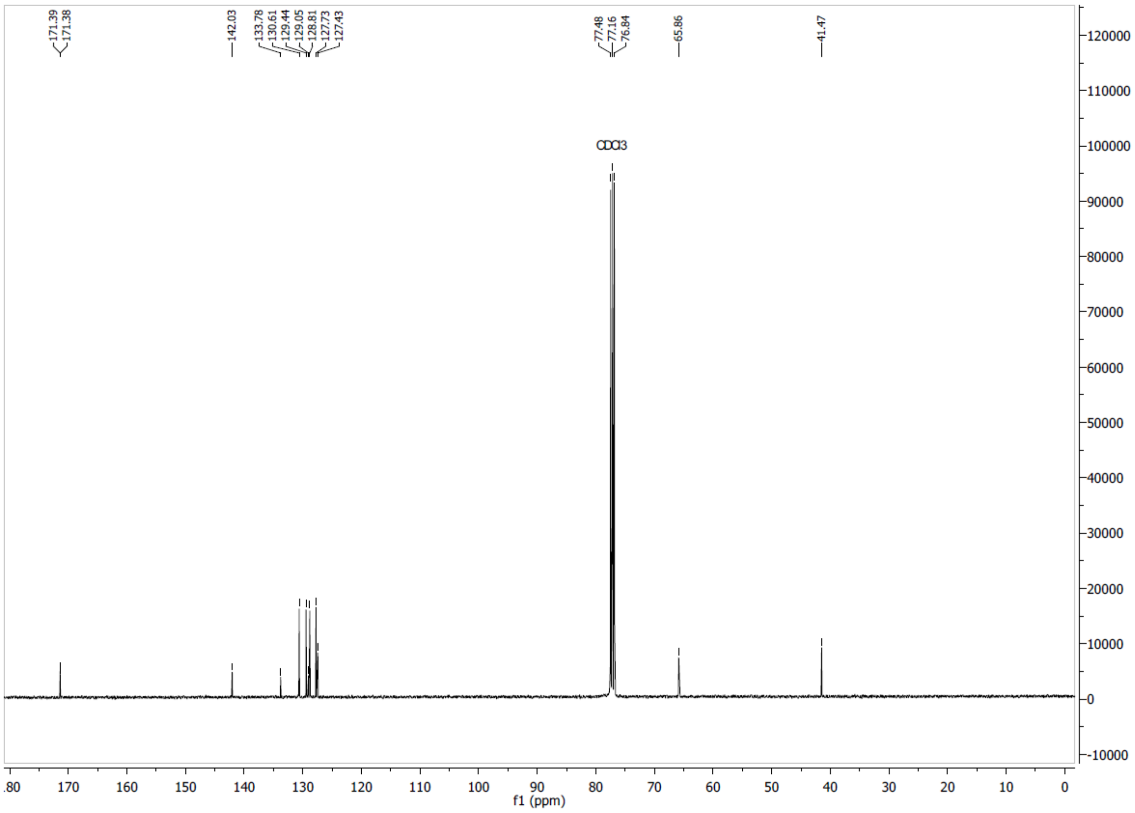

23. 4-(Benzoyloxymethyl)benzoic acid (29).

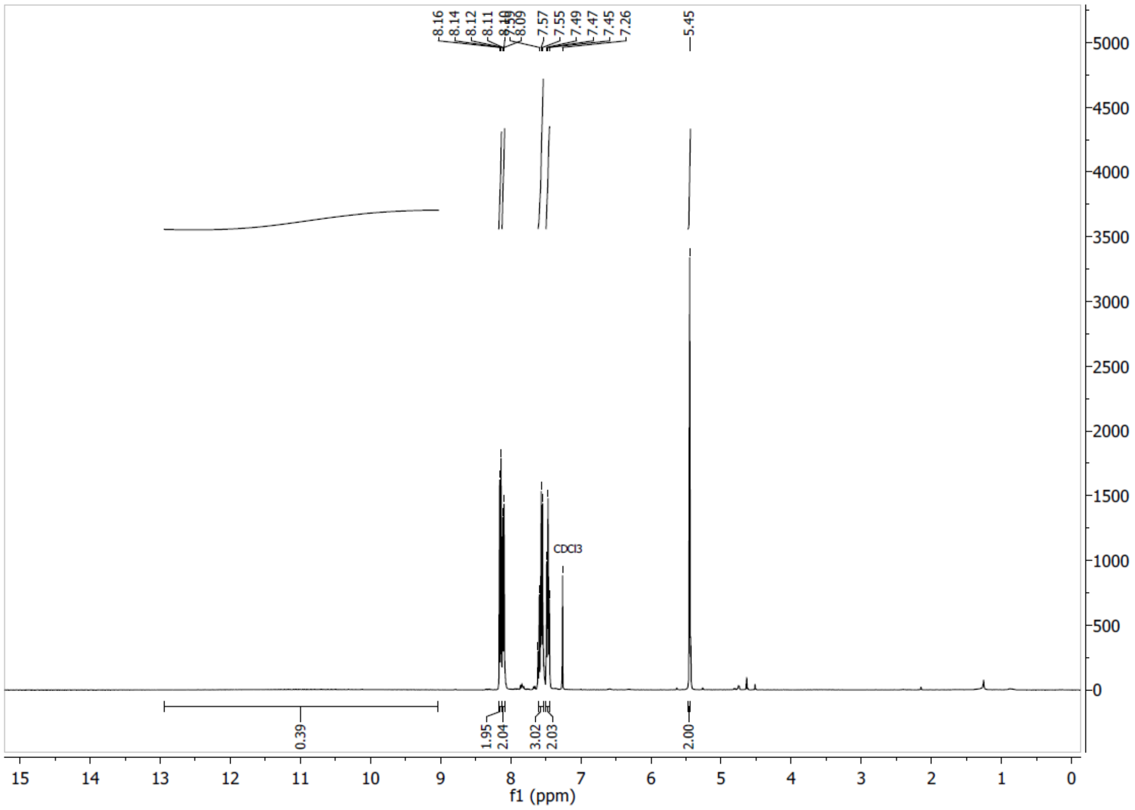

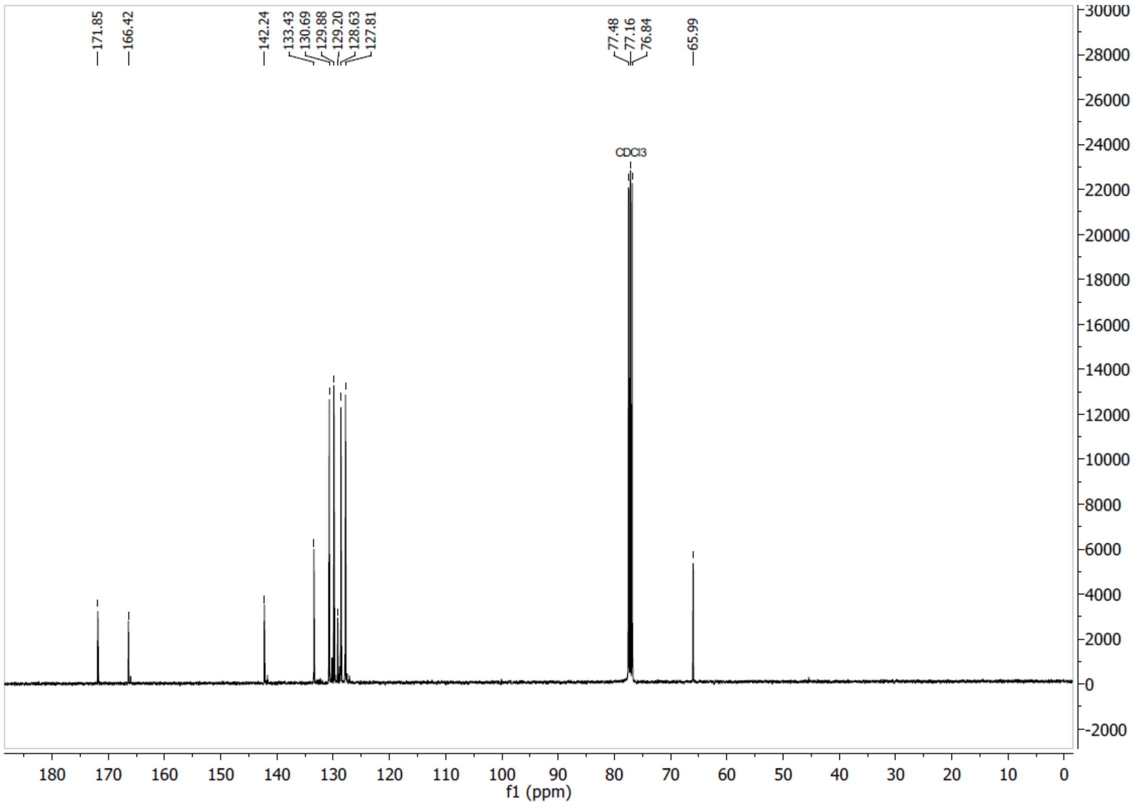

24. 4-(Acryloyloxymethyl)benzoic acid (30).

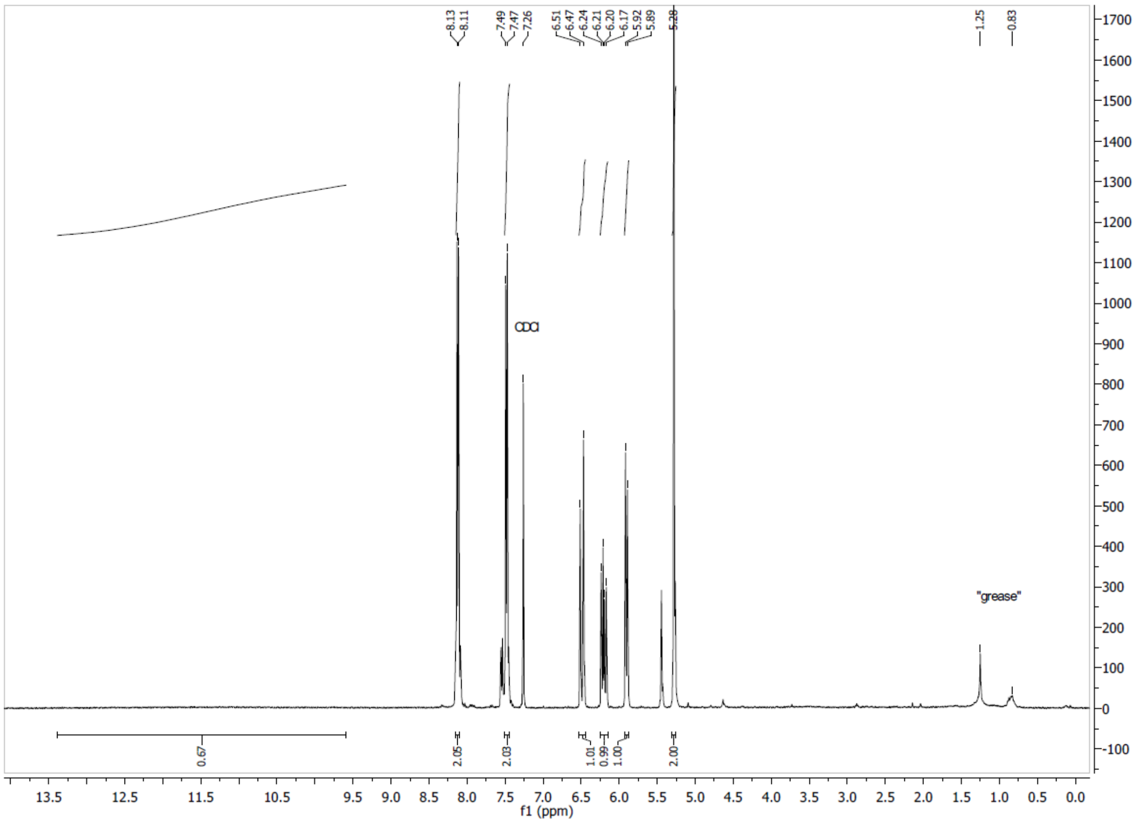

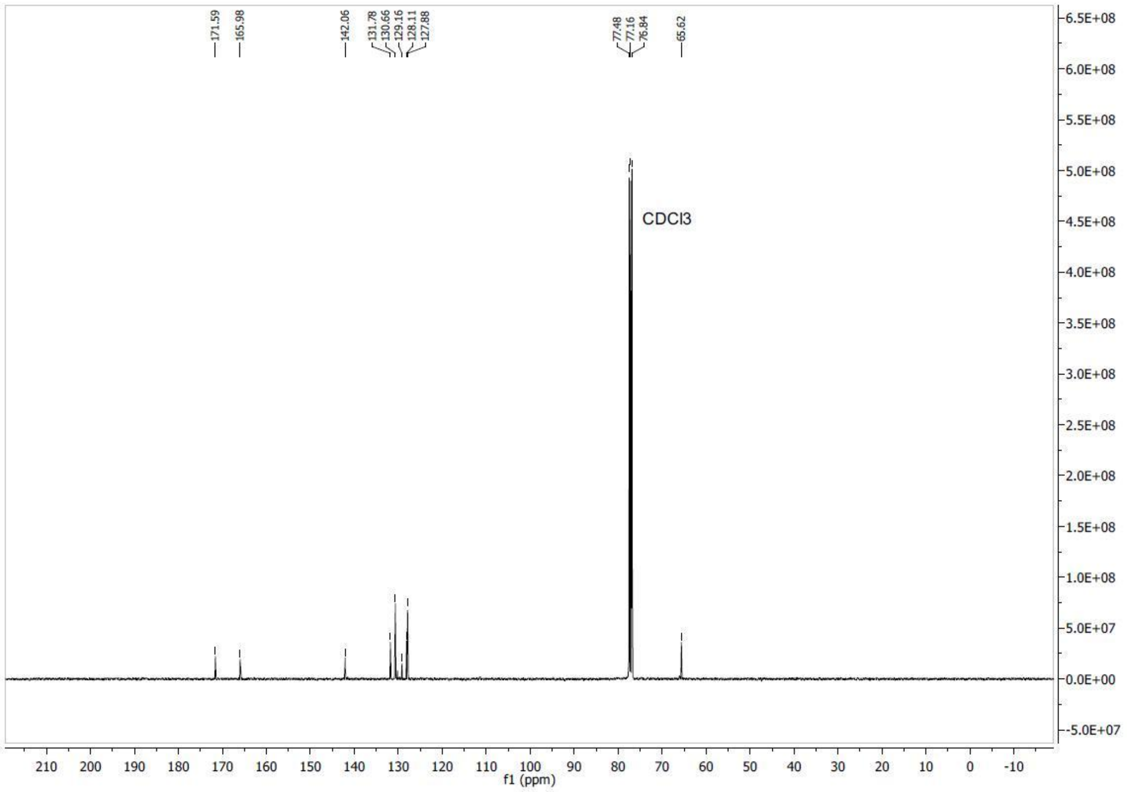

25. **Hexane-1,6-diyl diacetate (32).**

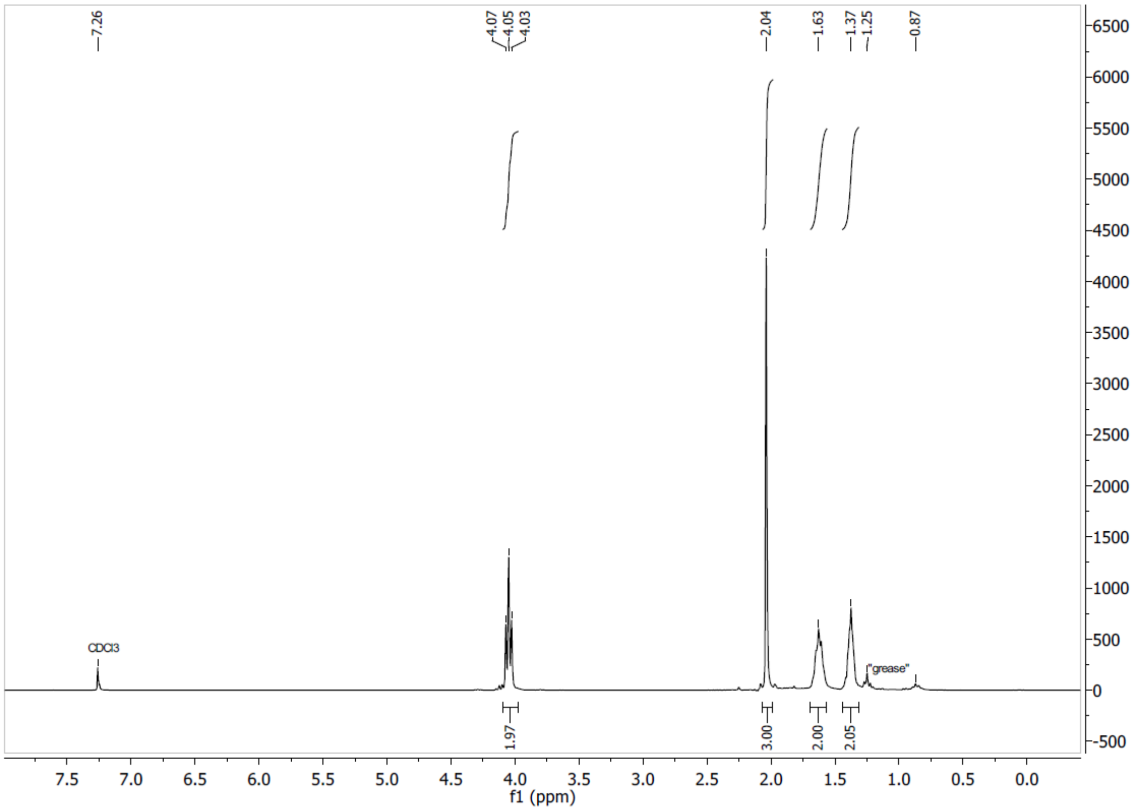

26. **6-Hydroxyhexyl acetate (33).**

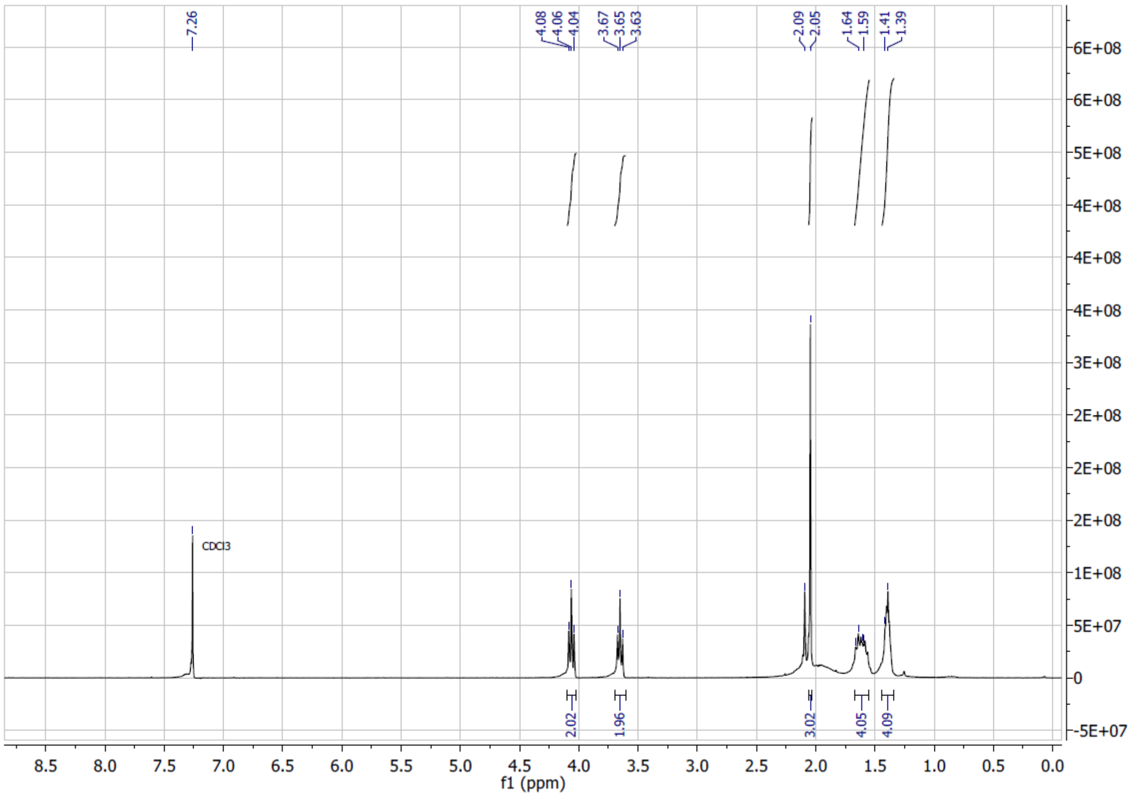

27. Hexane-1,6-diyl bis(phenylacetate) (34).

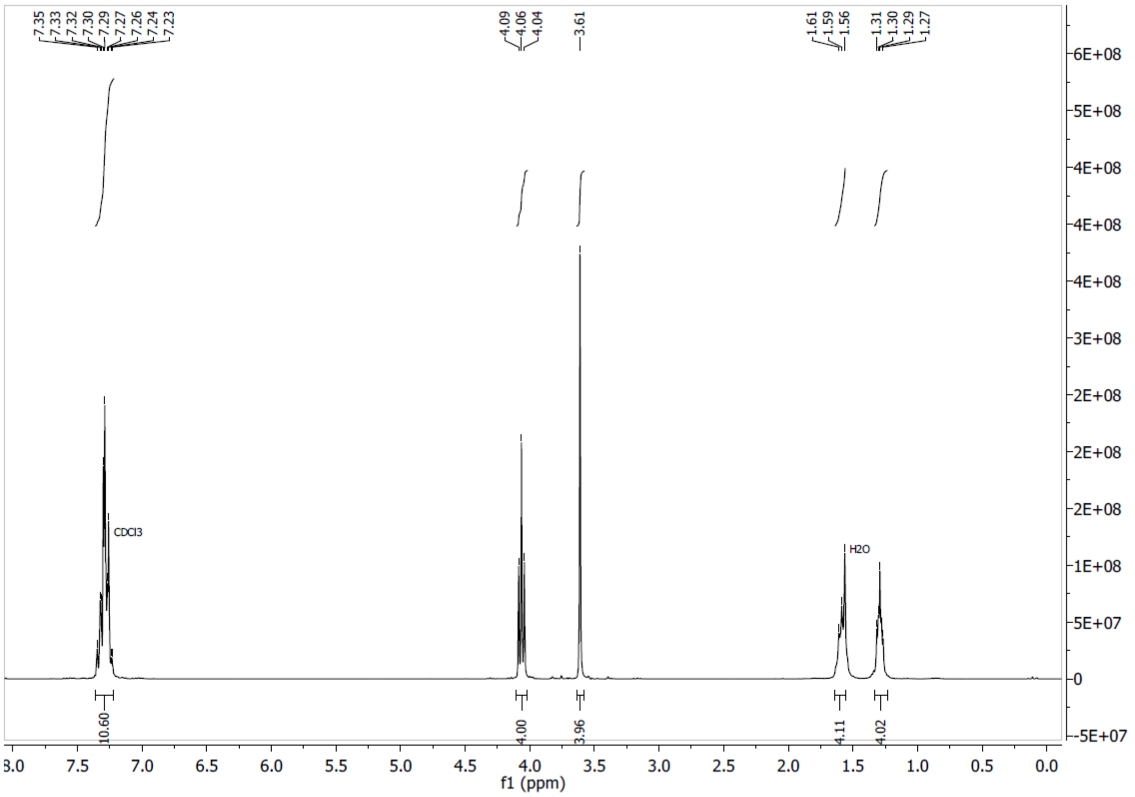

28. 6-Hydroxyhexyl phenylacetate (35).

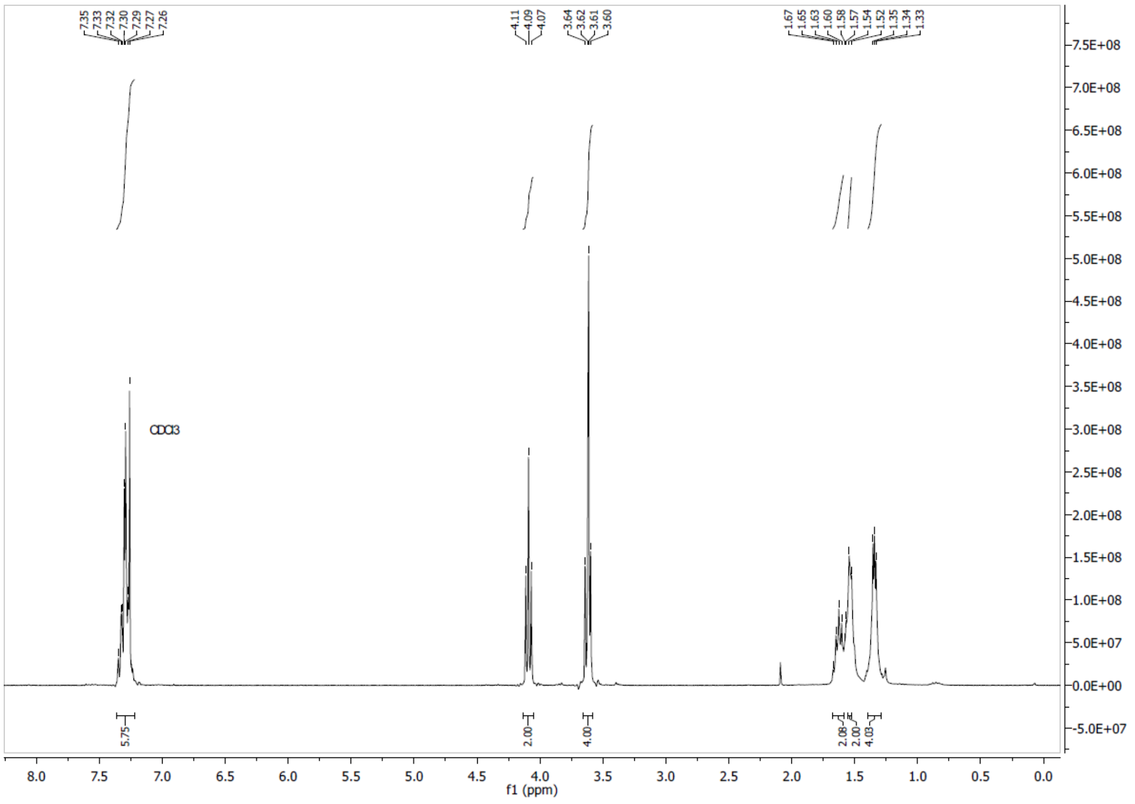

29. Ethyl 6-acetoxyhexanoate (37).

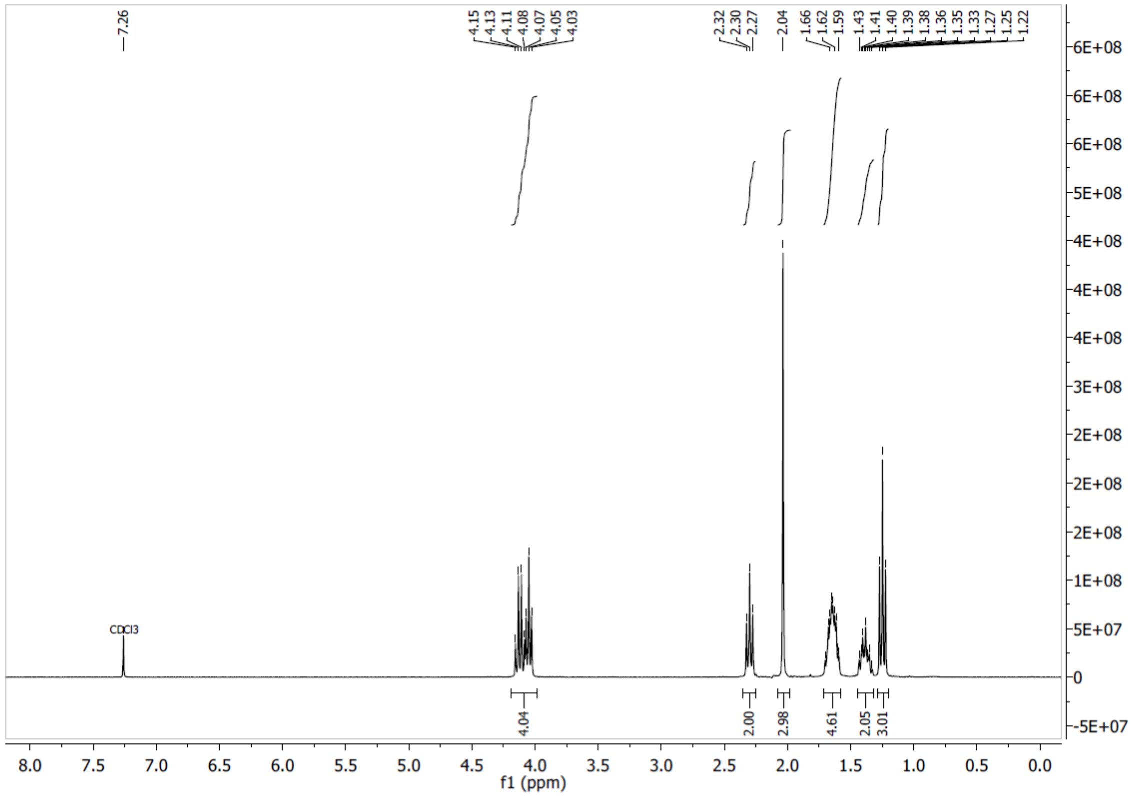

30. Ethyl 6-(phenylacetoxy)hexanoate (38).

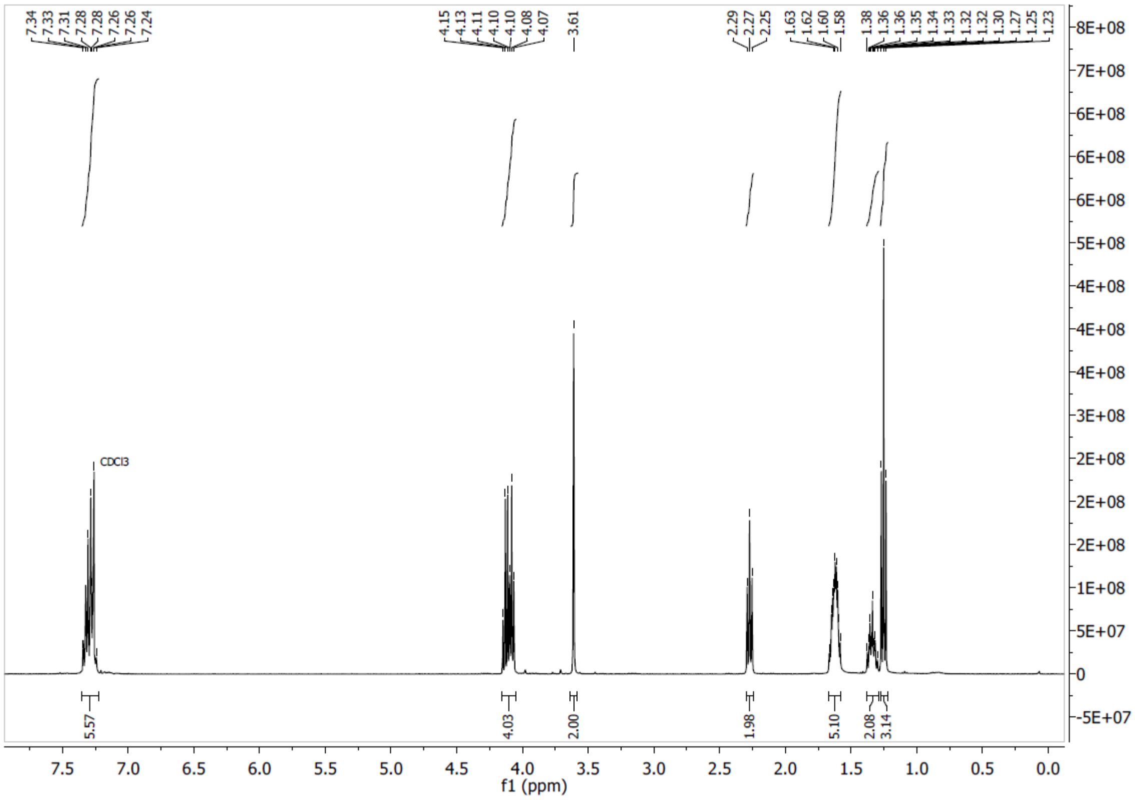

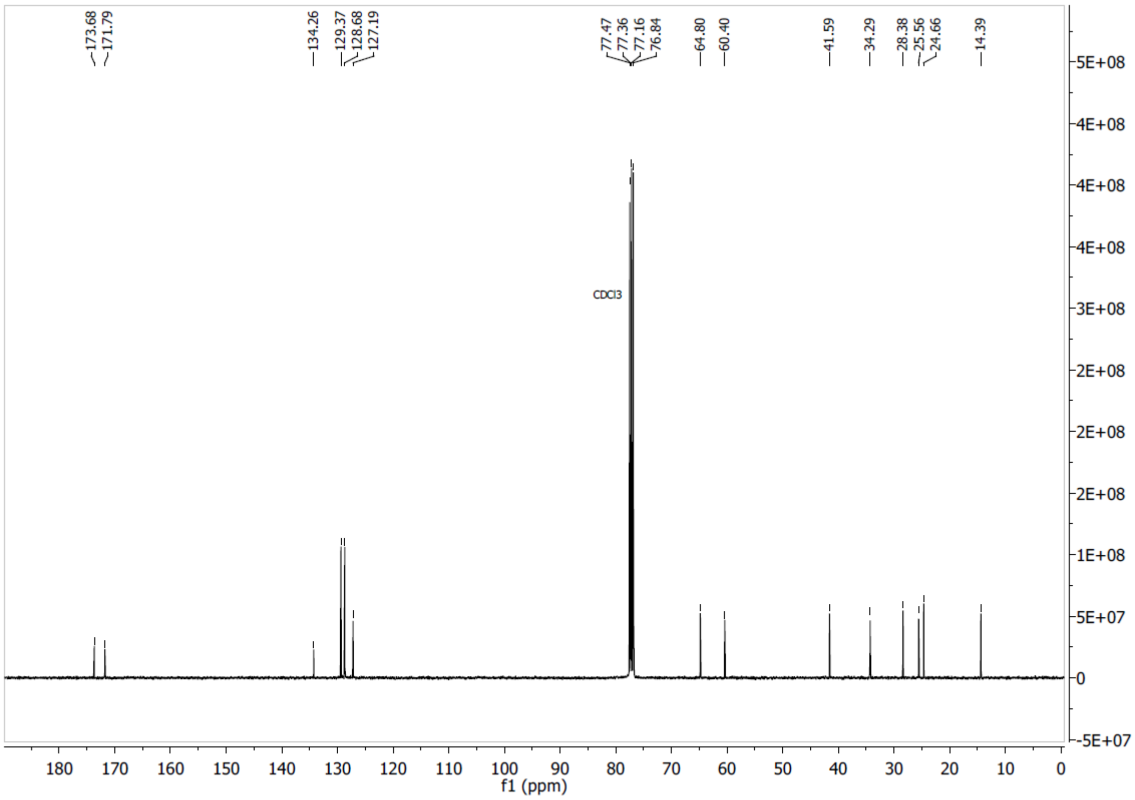

31. 6-Ethoxy-6-oxohexyl benzoate (39).

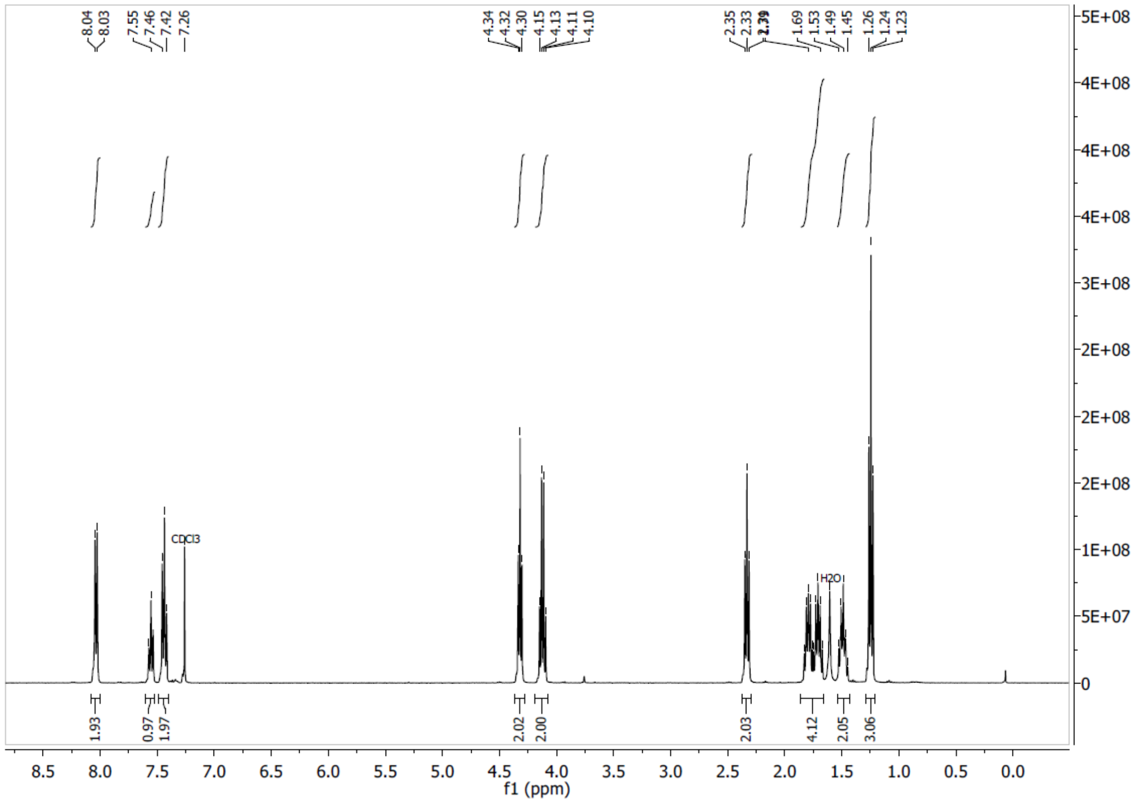

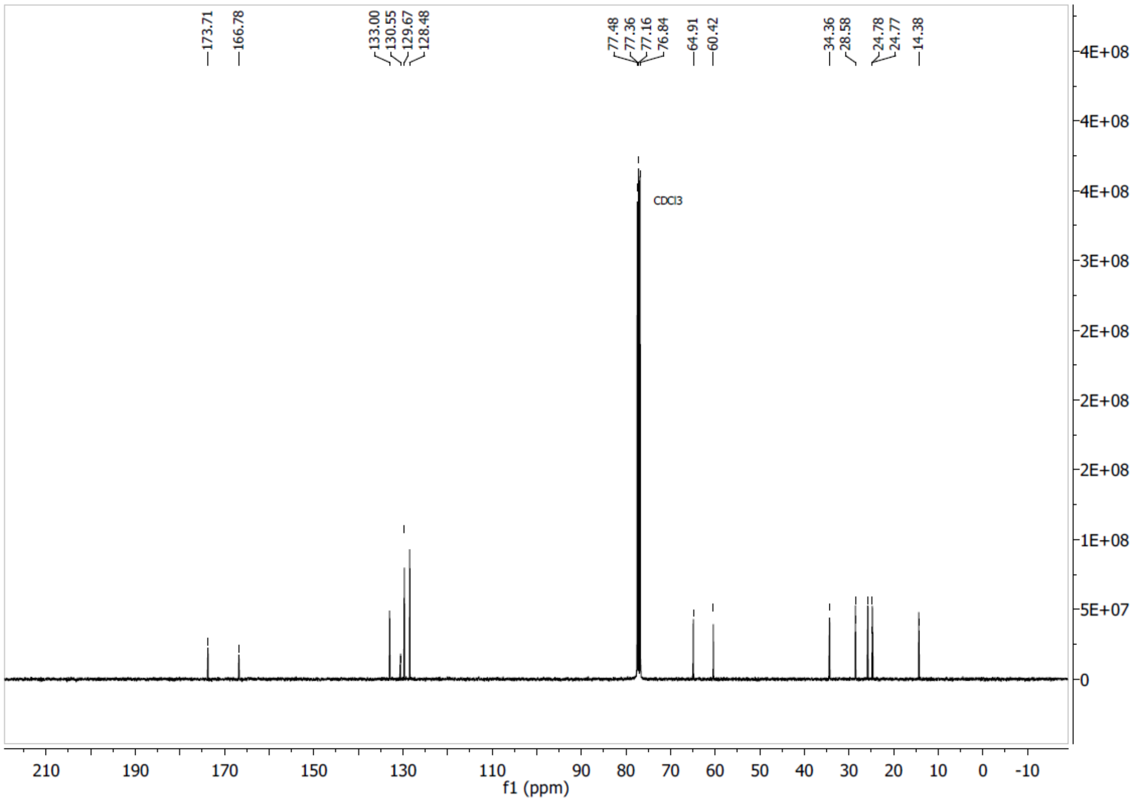

32. Ethyl 6-(acryloyloxy)hexanoate (40).

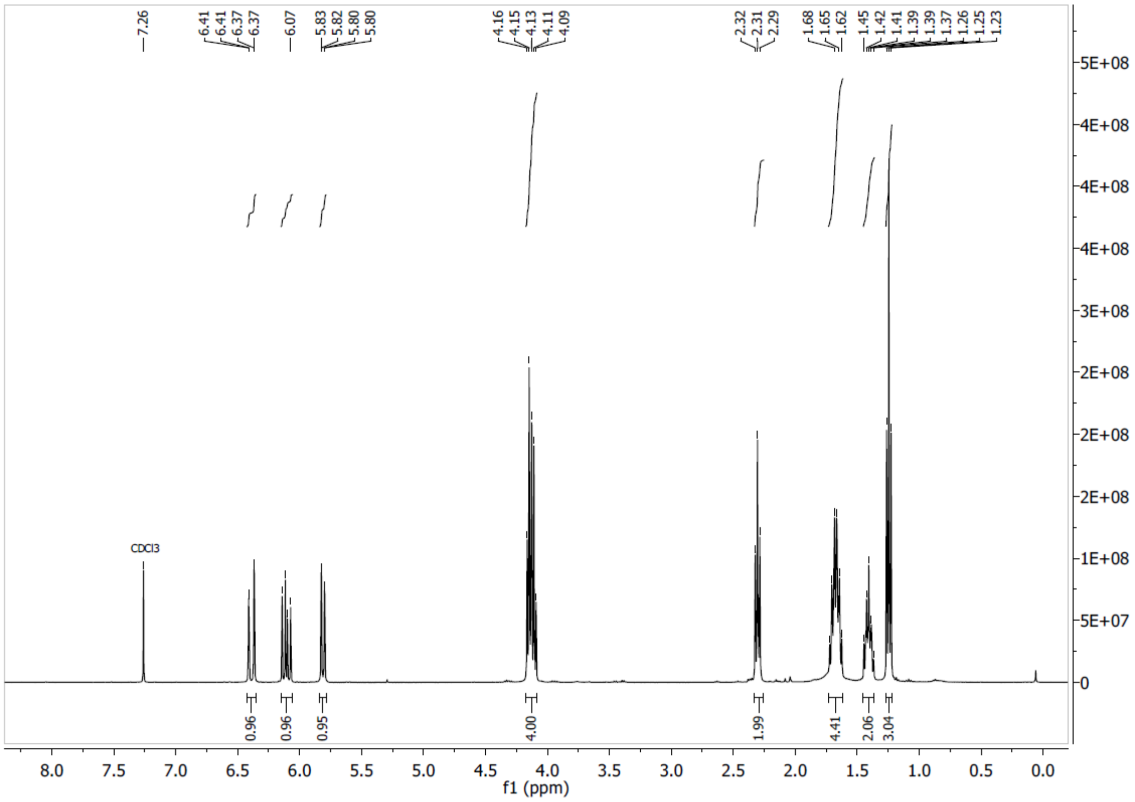

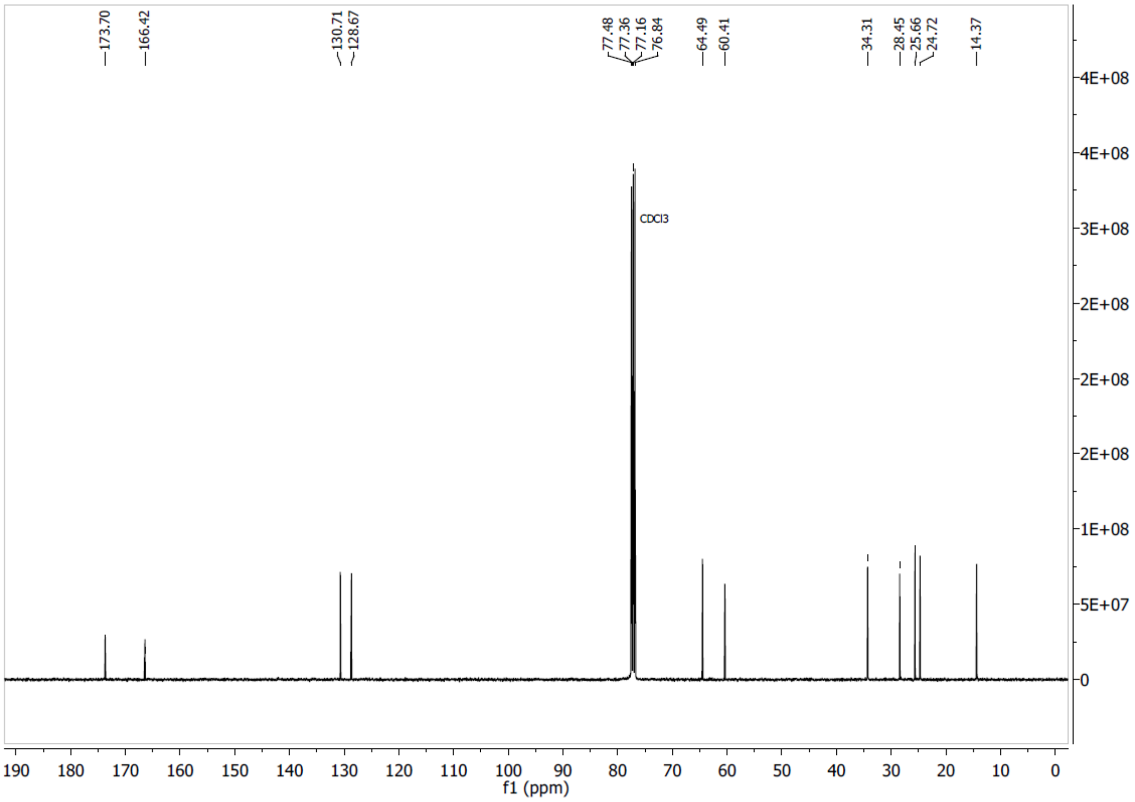

33. 4-(Benzyloxycarbonylamino)butyl acetate (42).

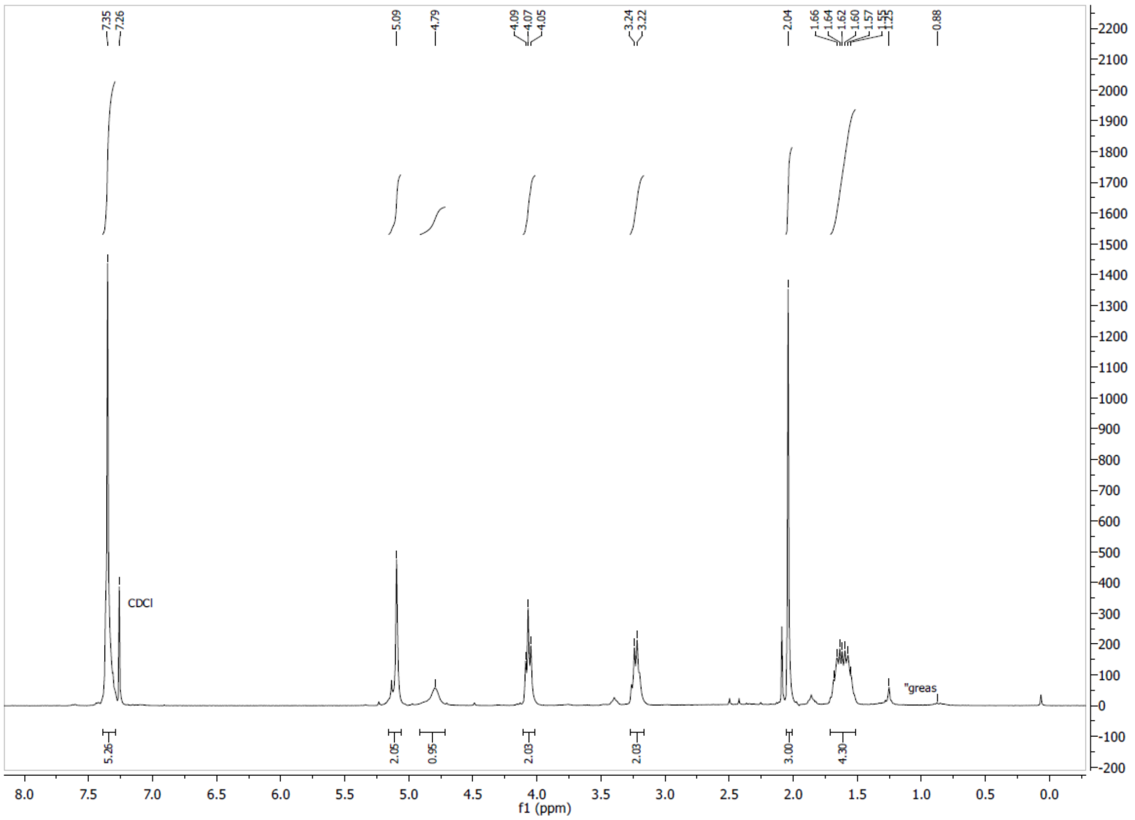

34. 4-(Benzyloxycarbonylamino)butyl phenylacetate (43).

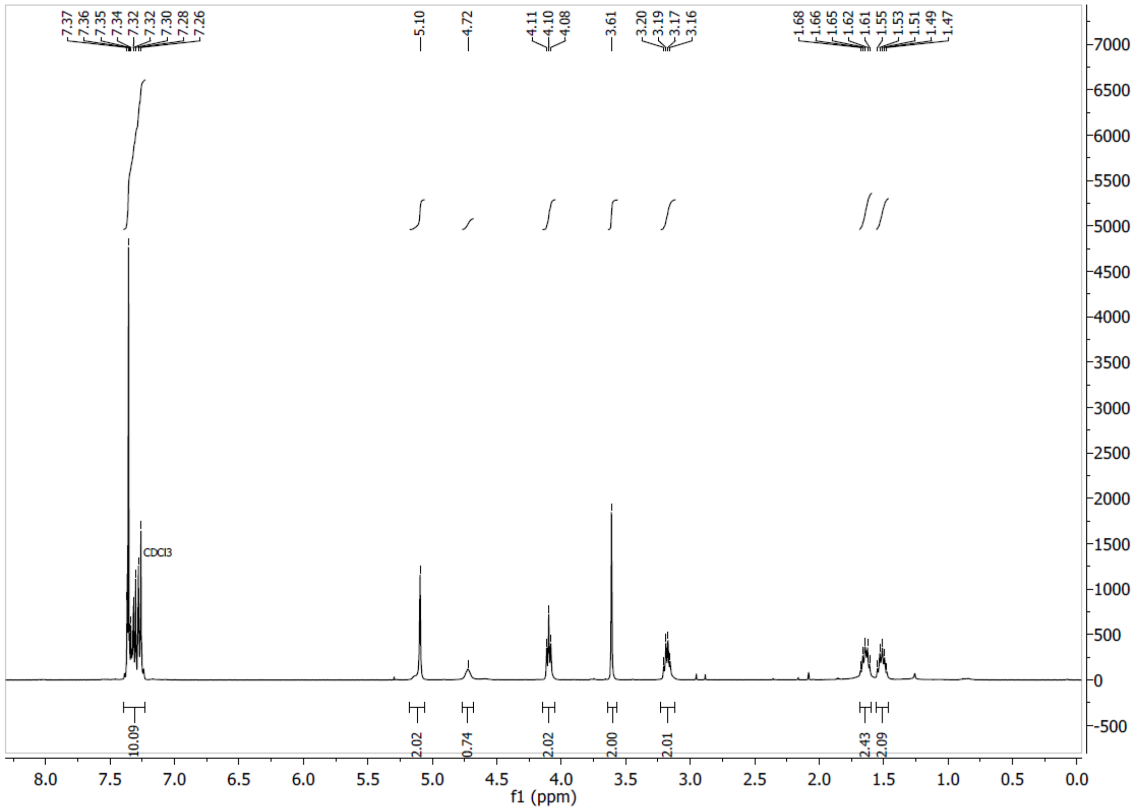

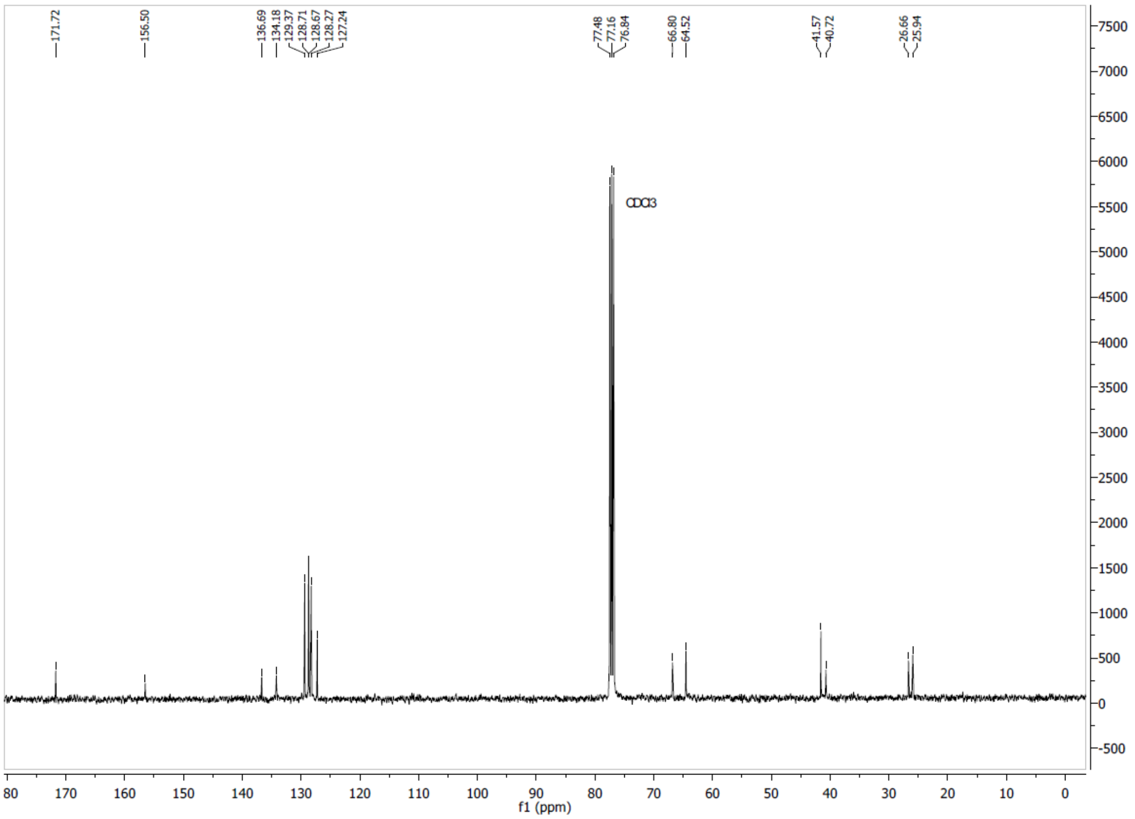

35. 4-(Benzyloxycarbonylamino)butyl acrylate (44).

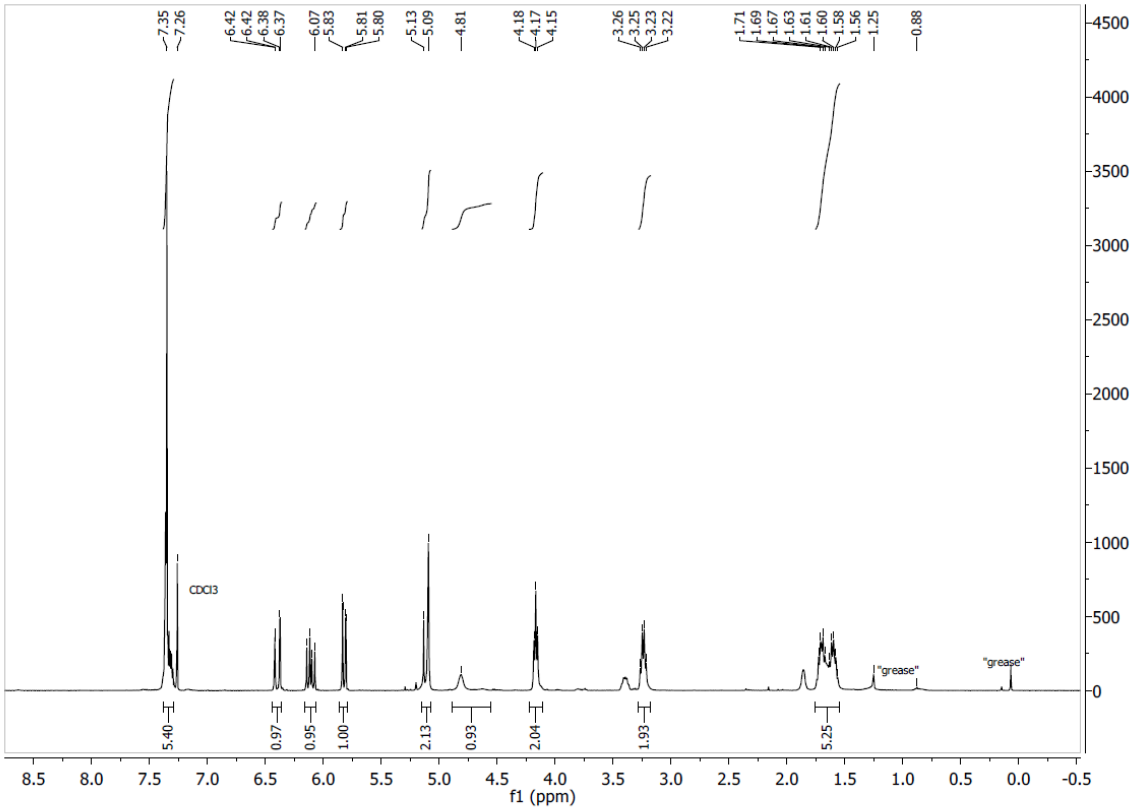

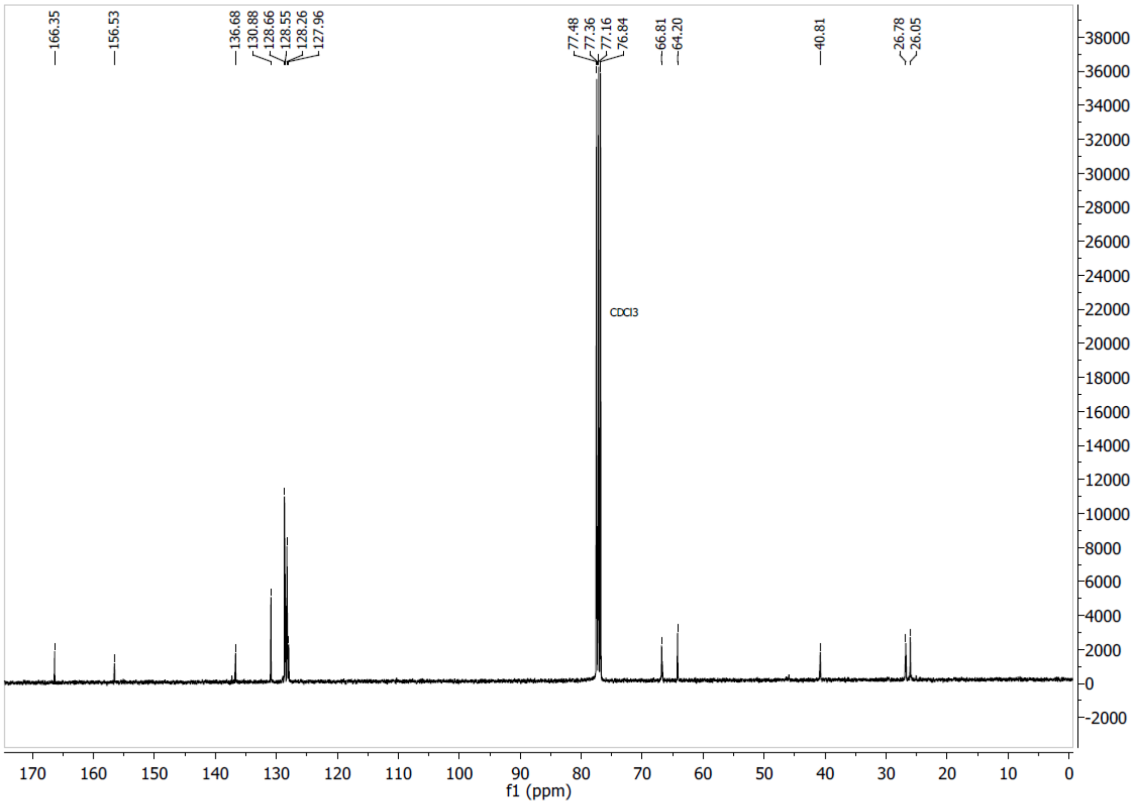

36. **Cyclohexyl acetate (46).**

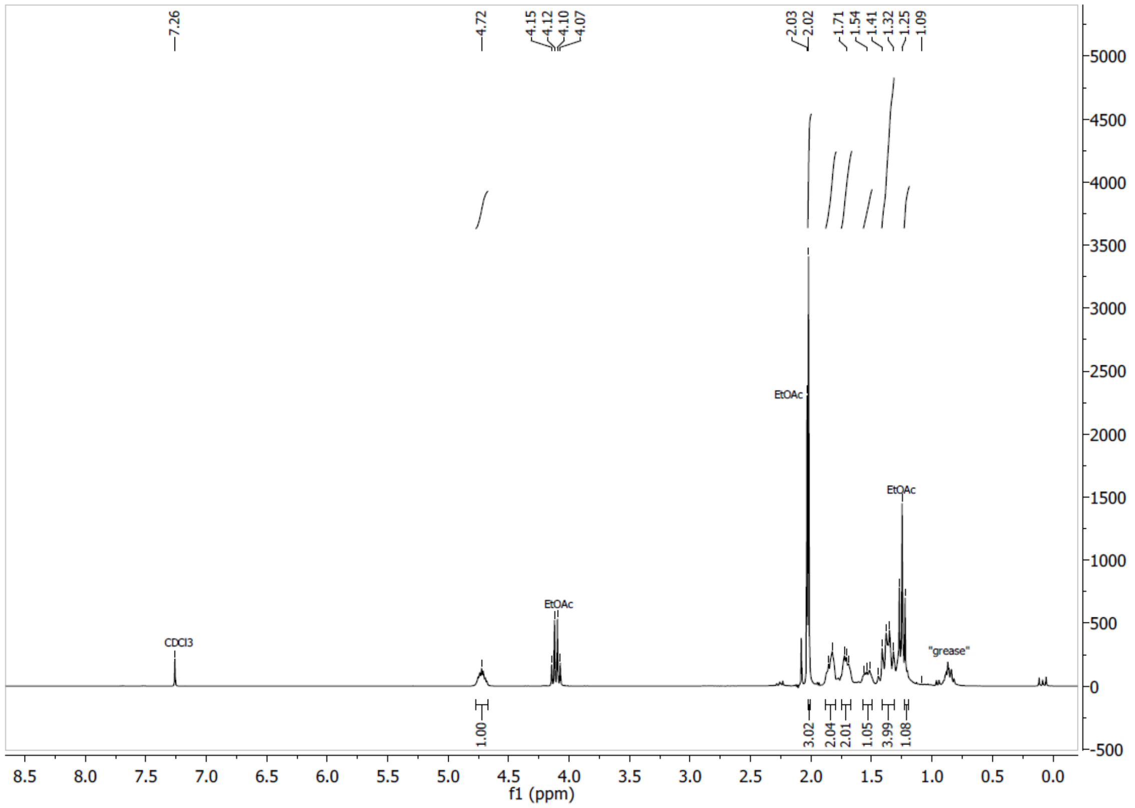

37. **Cyclohexyl phenylacetate (47).**

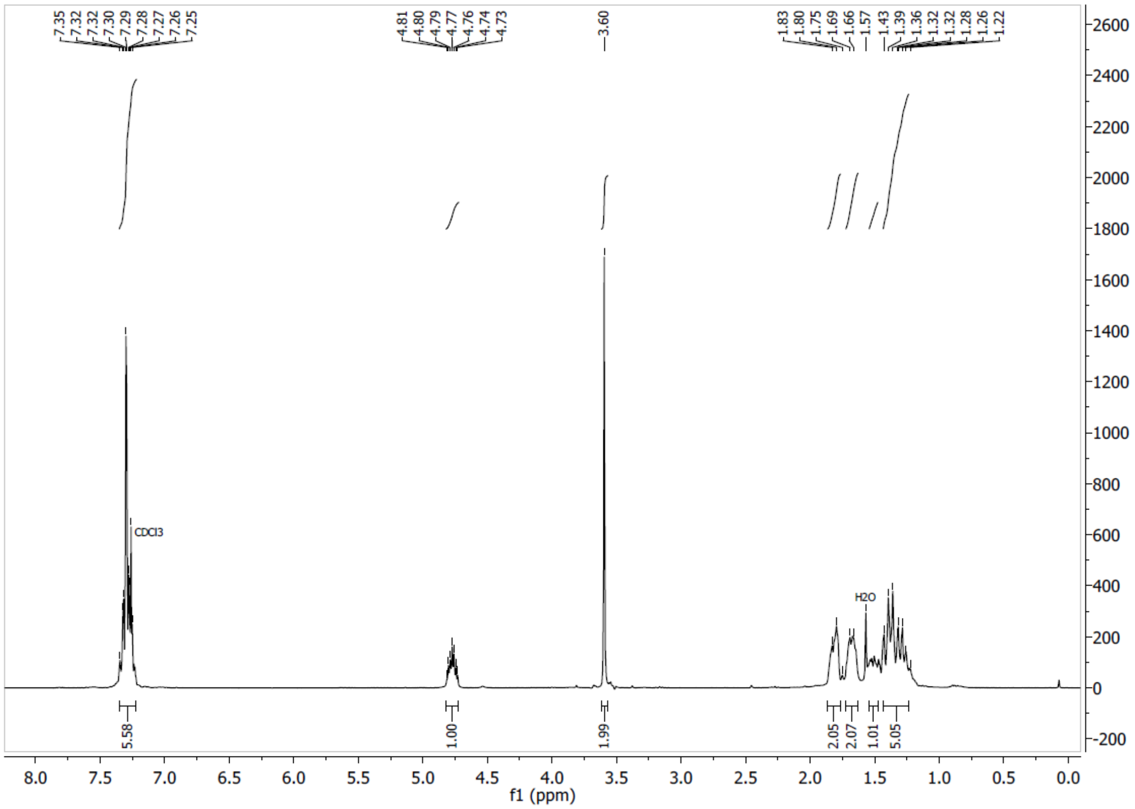

38. 2-(4-Hydroxyphenyl)ethyl acetate (54).

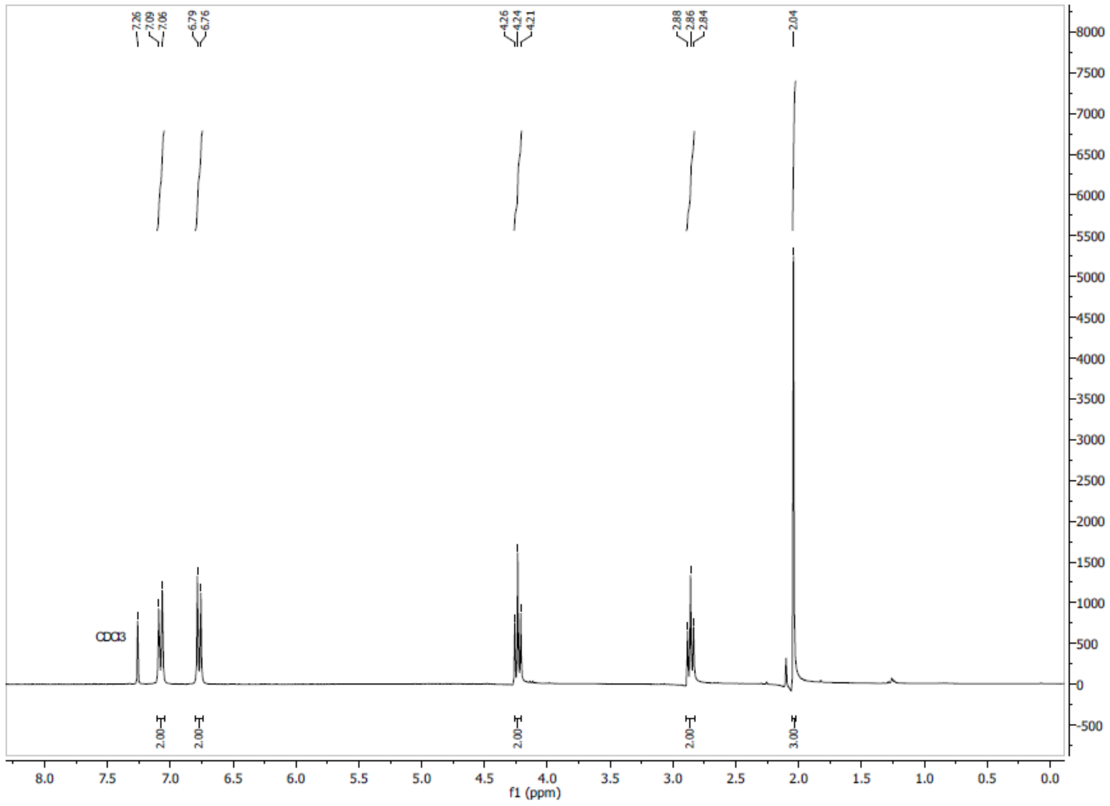

39. 2-(4-Hydroxyphenyl)ethyl phenylacetate, monaspilosin (55).

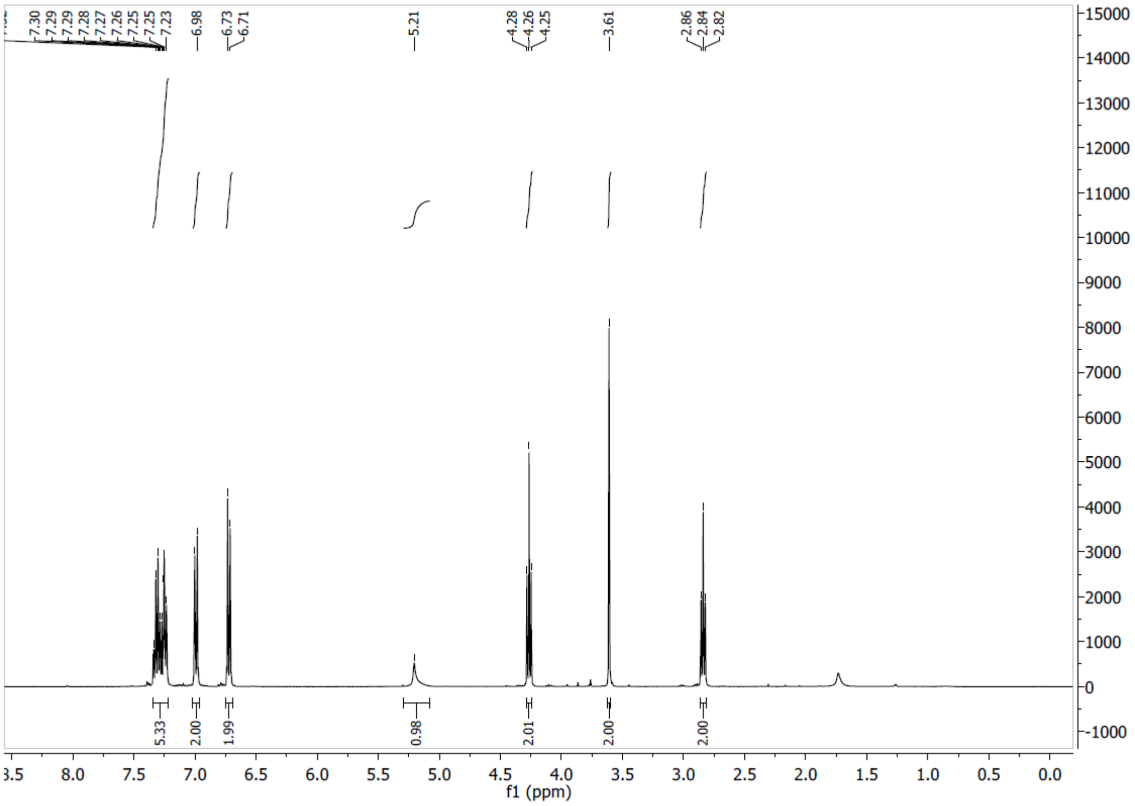

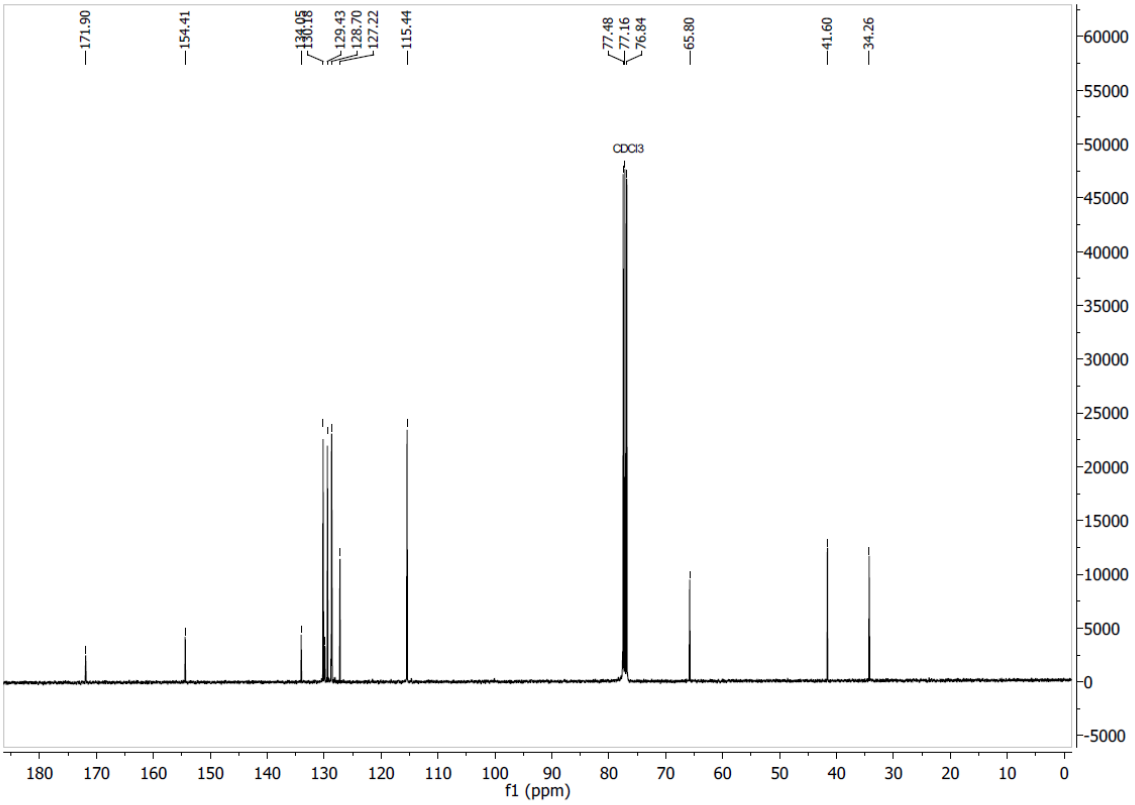

40. Benzyl acetate (57).

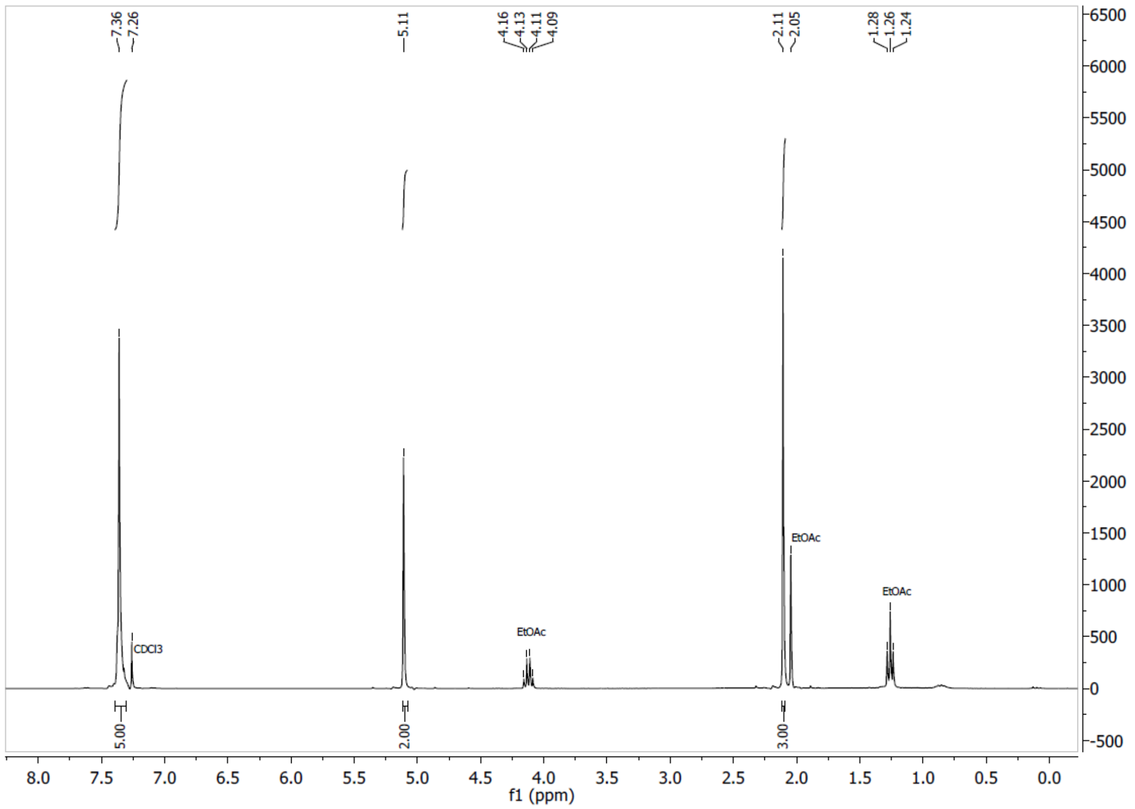

41. *N*-Benzylacetamide (58)

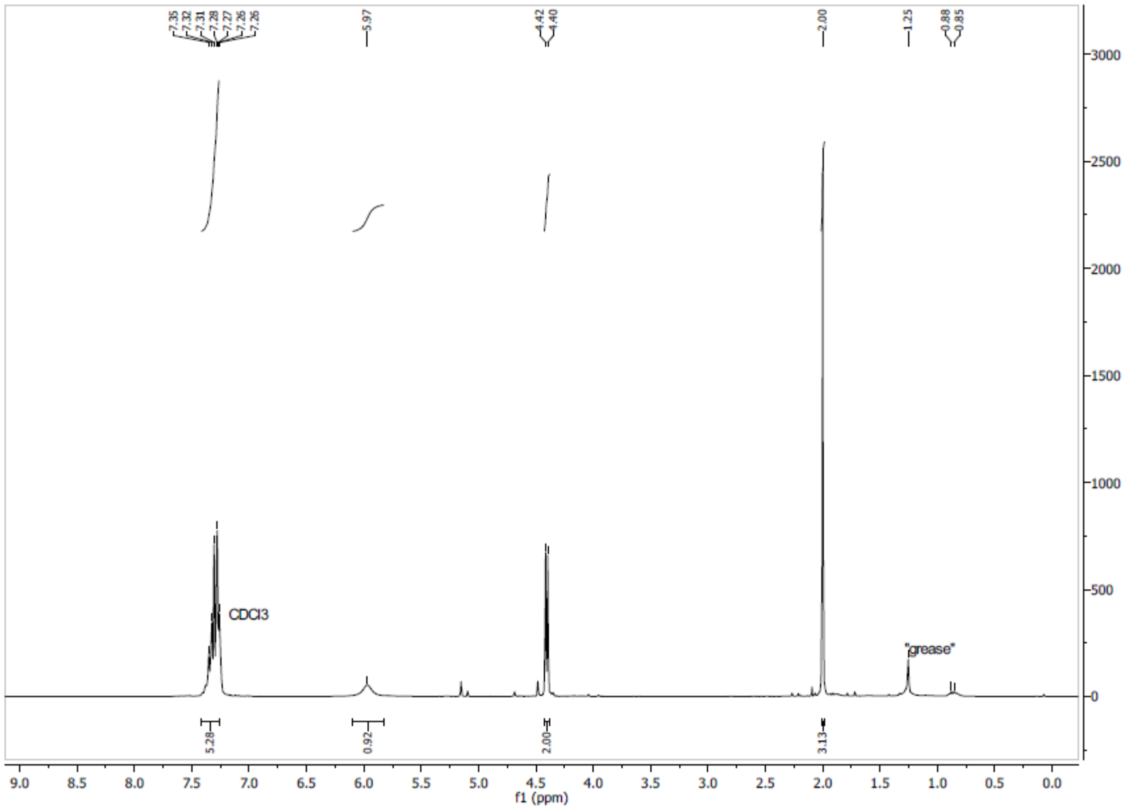

42. Benzyl phenylacetate (59).

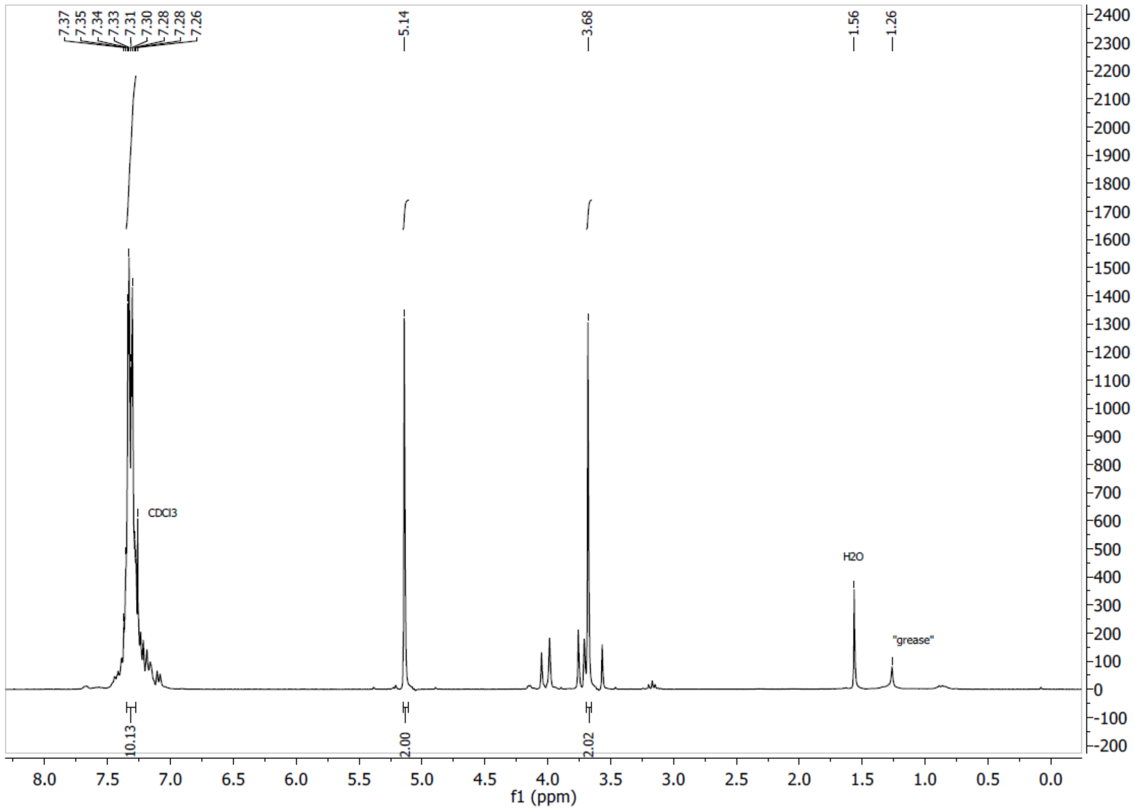

43. *N*-Benzyl-phenylacetamide (60).

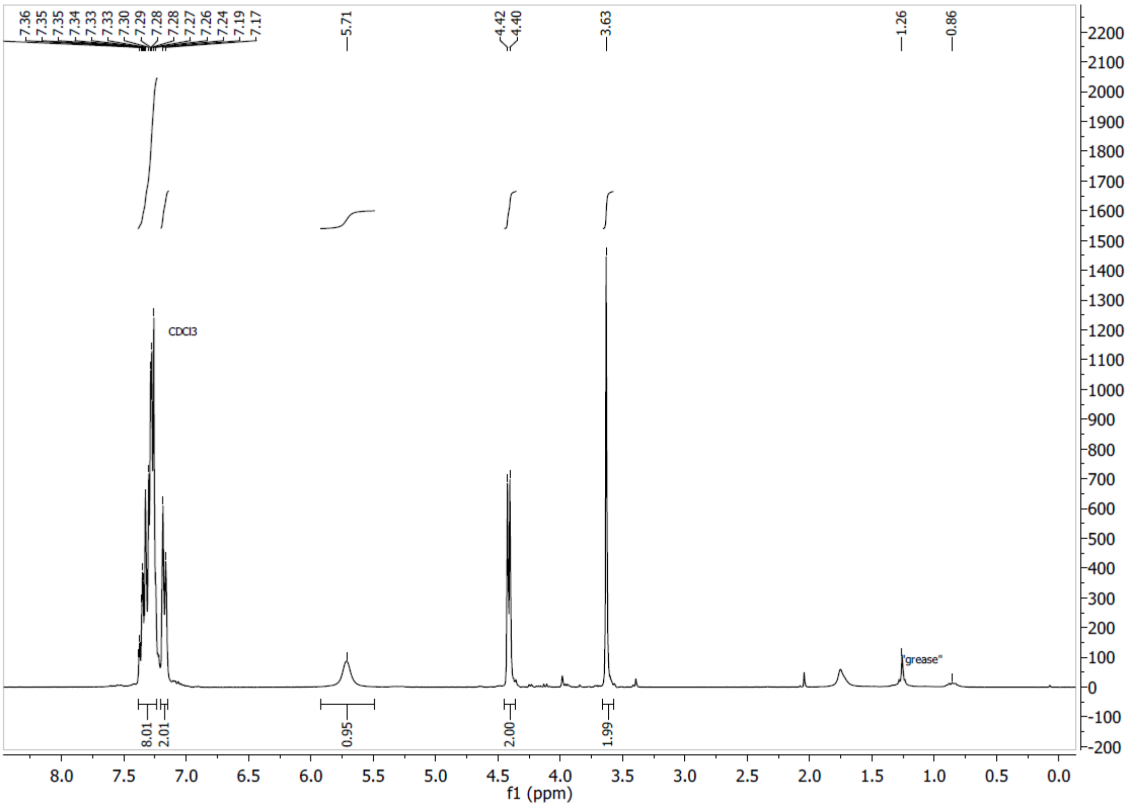

44. *N*-Benzylbenzamide (61).

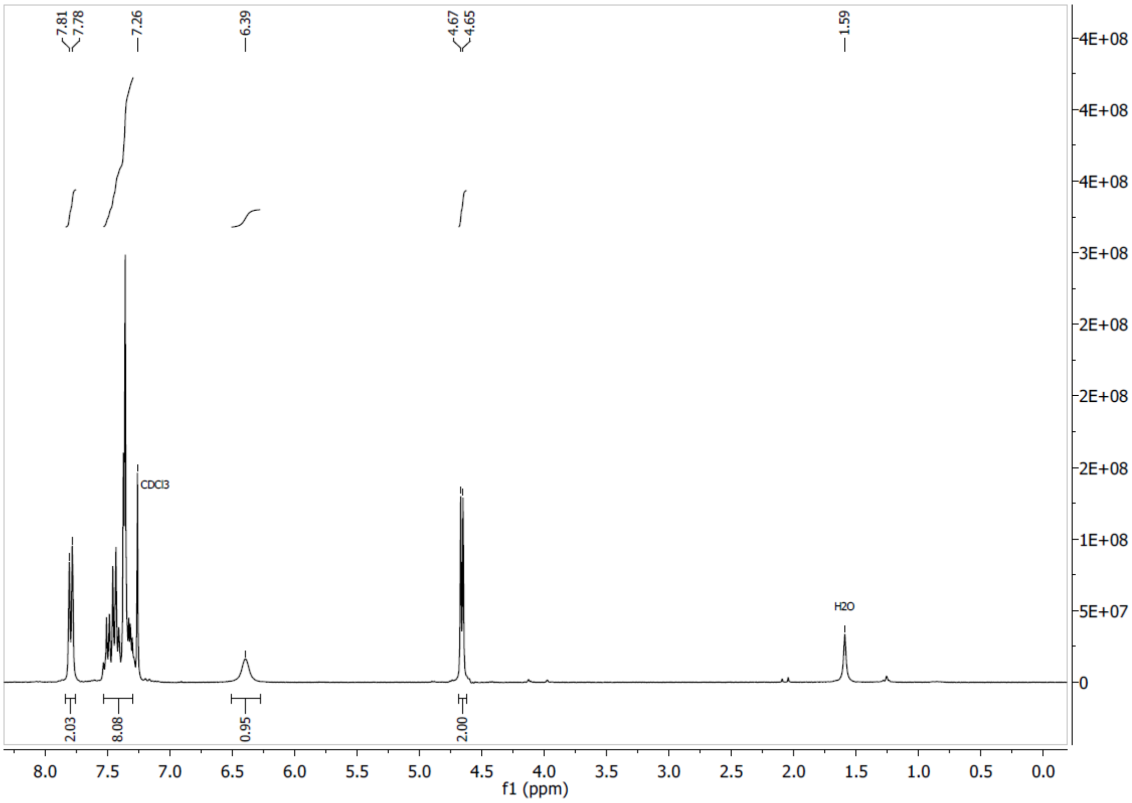

45. Benzyl acrylate (62).

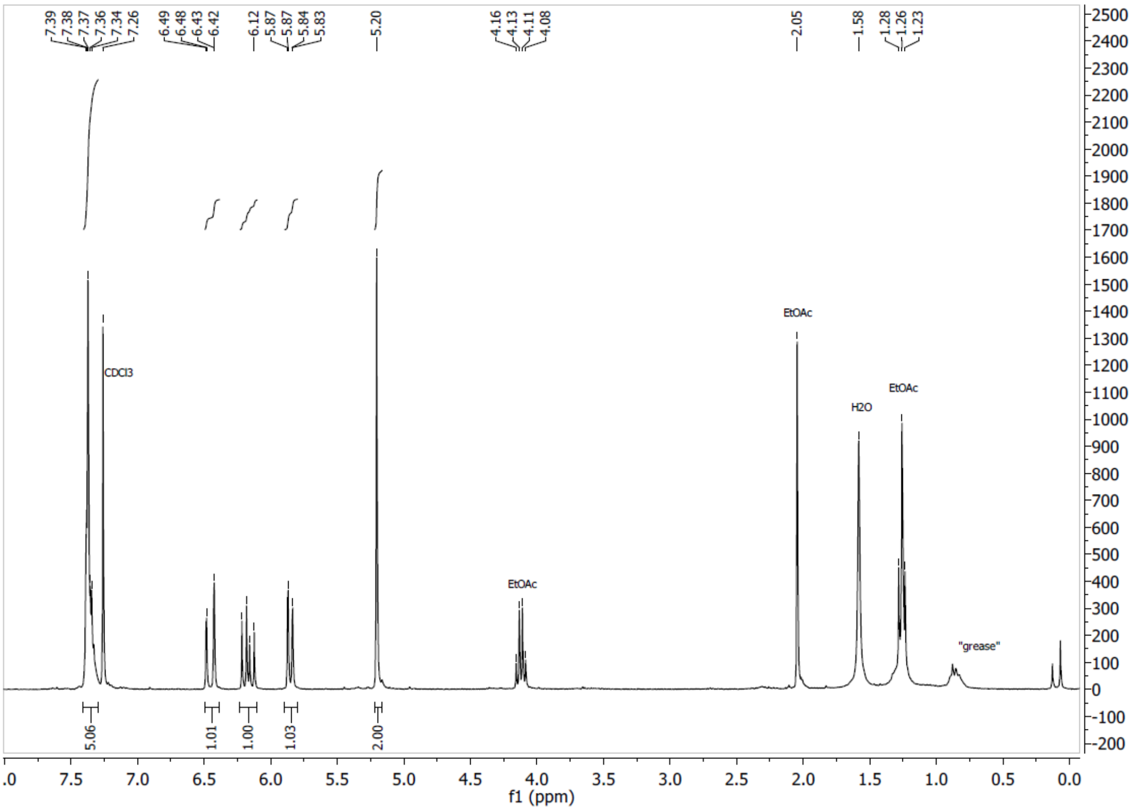

46. N-Benzylacrylamide (63)

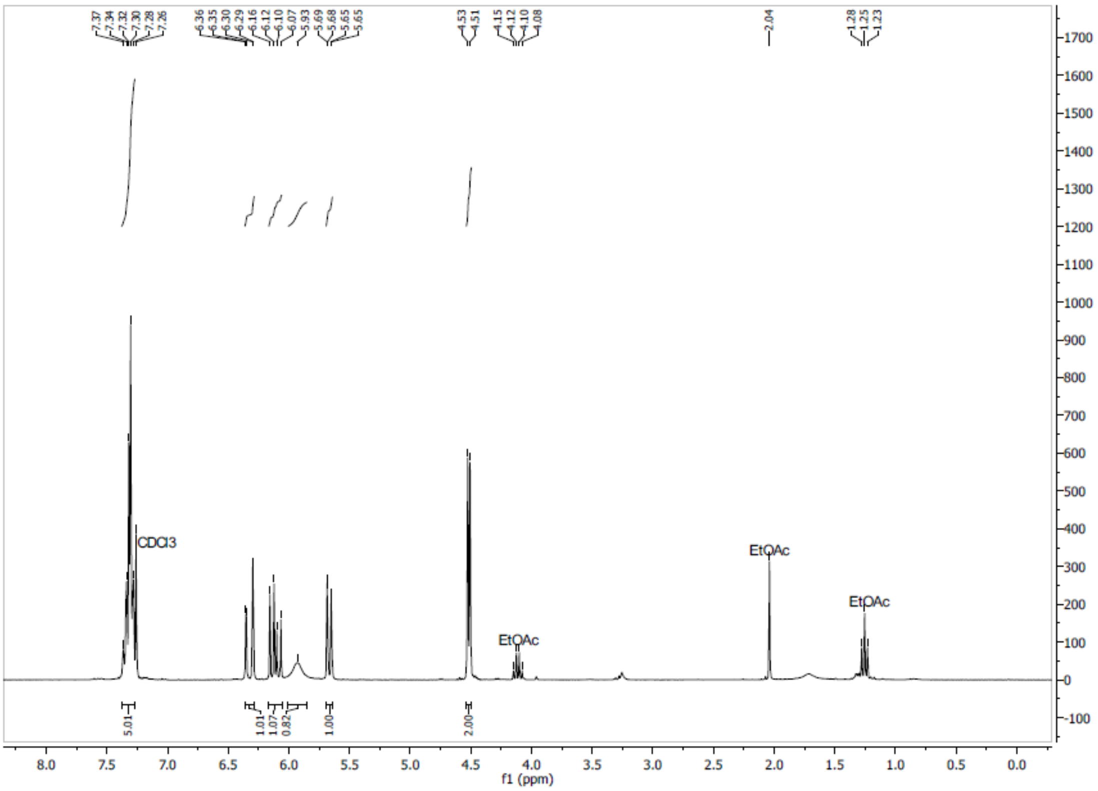

47. 4-Fluorobenzyl acetate (65).

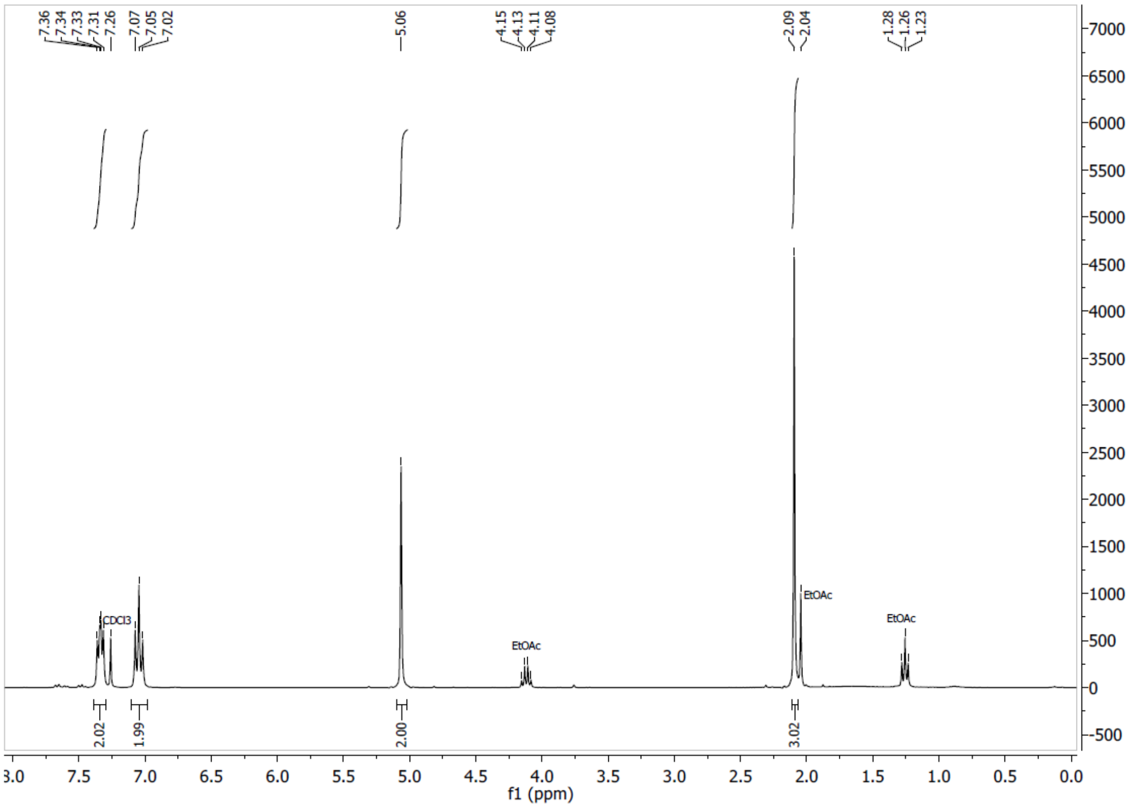

48. *N*-4-(Fluorobenzyl)acetamide (66).

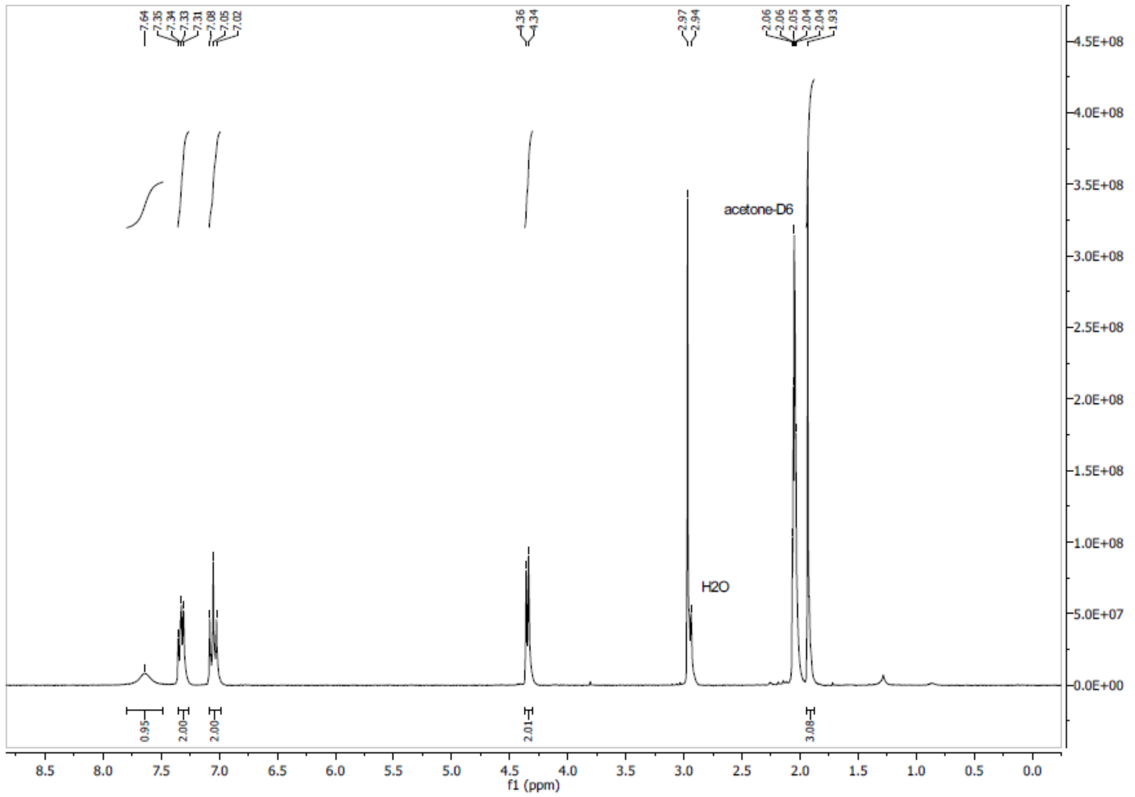

49. ***N*-(4-Fluorobenzyl)phenylacetamide (67).**

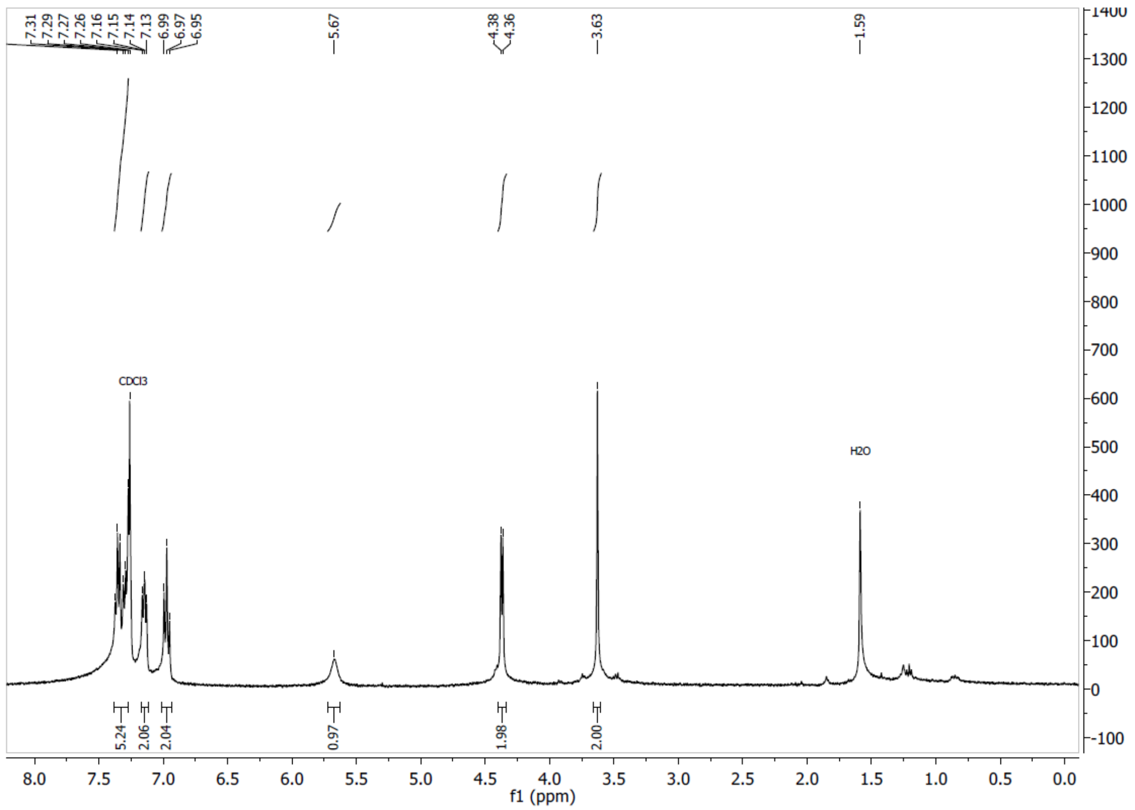

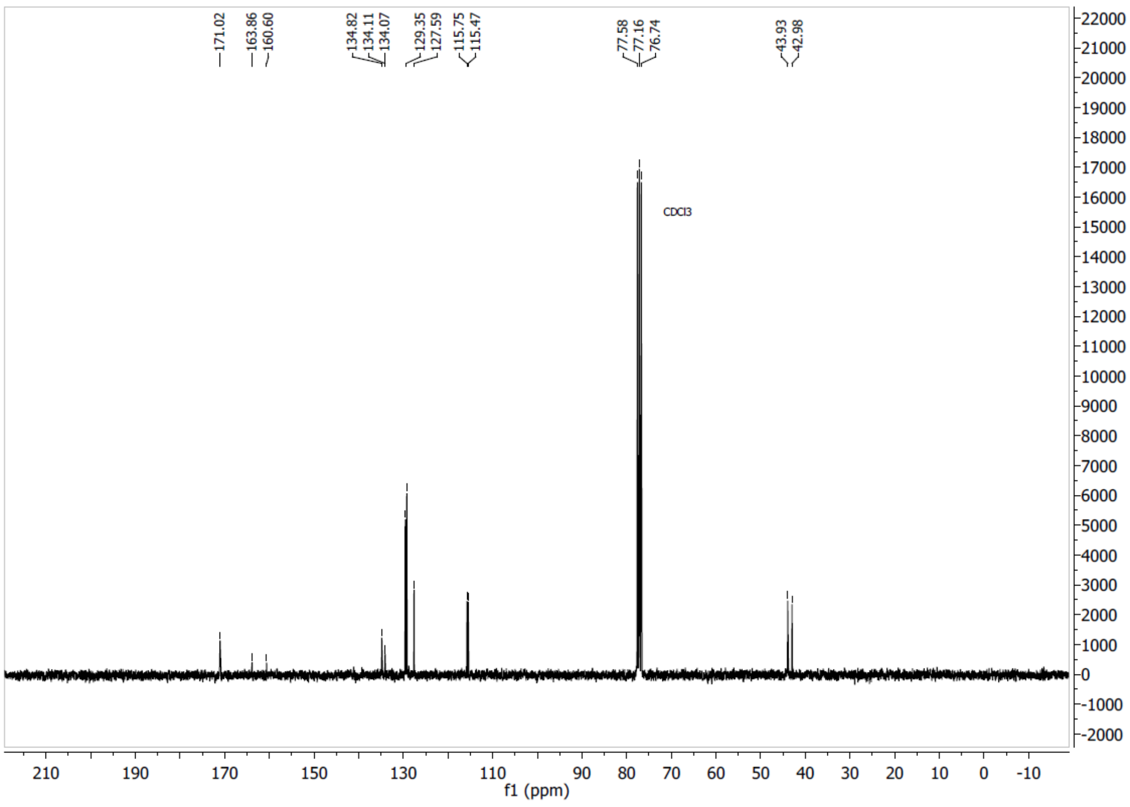

50. ***N*-(4-Fluorobenzyl)benzamide (68).**

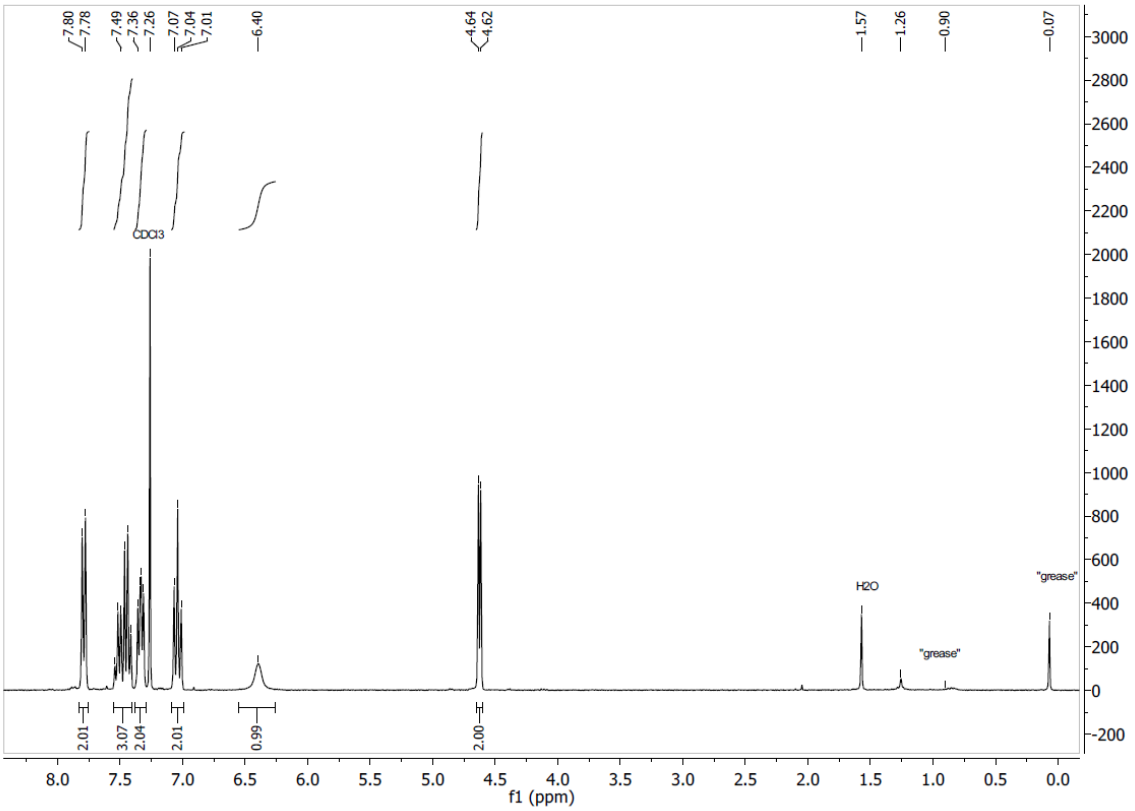

51. ***N*****-(4-Fluorobenzyl)acrylamide (69).**

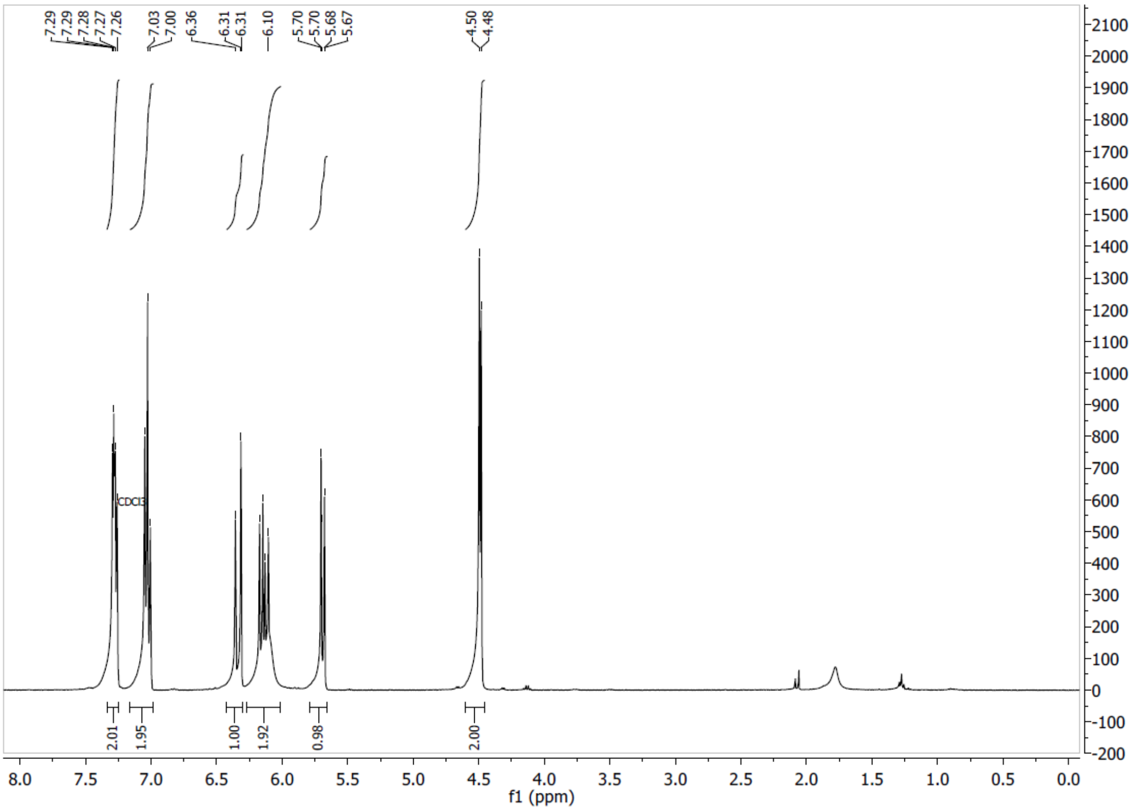

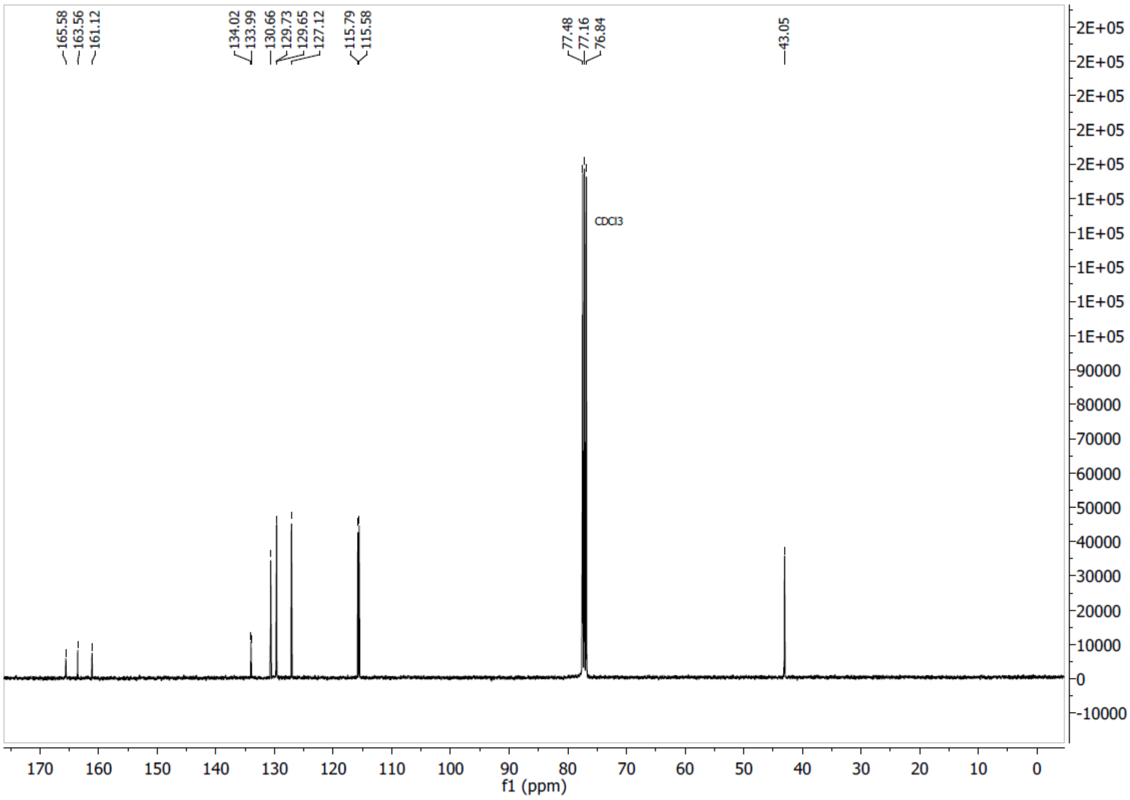

Supplement: File 1 — Experimental section and NMR spectra of all synthesized compounds. [file Beilstein_J_Org_Chem-09-1572-s001.pdf]
